# From Chains to Chromophores: Tailored Thermal and Linear/Nonlinear Optical Features of Asymmetric Pyrimidine—Coumarin Systems

**DOI:** 10.3390/molecules30214322

**Published:** 2025-11-06

**Authors:** Prescillia Nicolas, Stephania Abdallah, Dong Chen, Giorgia Rizzi, Olivier Jeannin, Koen Clays, Nathalie Bellec, Belkis Bilgin-Eran, Huriye Akdas-Kiliç, Jean-Pierre Malval, Stijn Van Cleuvenbergen, Franck Camerel

**Affiliations:** 1 Institut des Sciences Chimiques de Rennes, CNRS-UMR 6226, Université de Rennes, 35042 Rennes, France; prescillia.nicolas05565@gmail.com (P.N.); olivier.jeannin@univ-rennes.fr (O.J.); nathalie.bellec@univ-rennes.fr (N.B.); hakdas@yildiz.edu.tr (H.A.-K.); 2 Institut de Science des Matériaux de Mulhouse, CNRS-UMR 7361, Université de Haute Alsace, 68093 Mulhouse, France; stephania.abdallah@uha.fr; 3 Department of Chemistry, Katholieke Universiteit Leuven, 3000 Leuven, Belgium; dong.chen@kuleuven.be (D.C.); giorgia.rizzi@kuleuven.be (G.R.); koen.clays@kuleuven.be (K.C.); 4 Department of Chemistry, Yildiz Technical University, 34469 Istanbul, Turkey; bbilgin@yildiz.edu.tr

**Keywords:** asymmetric pyrimidine, thermal behavior, luminescence, two-photon absorption, second harmonic generation

## Abstract

Eleven novel asymmetric pyrimidine derivatives were synthesized. The pyrimidine core was functionalized with a coumarin chromophore and a pro-mesogenic fragment bearing either chiral or linear alkyl chains of variable length and substitution patterns. The thermal properties were investigated using polarized optical microscopy, differential scanning calorimetry, and small-angle X-ray scattering, revealing that only selected derivatives exhibited liquid crystalline phases with ordered columnar or smectic organizations. Linear and nonlinear optical properties were characterized by UV–Vis absorption, fluorescence spectroscopy, two-photon absorption, and second-harmonic generation. Optical responses were found to be highly sensitive to the substitution pattern: derivatives functionalized at the 4 and 3,4,5 positions exhibited enhanced 2PA cross-sections and pronounced SHG signals, whereas variations in alkyl chain length exerted only a minor influence. Notably, compounds forming highly ordered non-centrosymmetric mesophases produced robust SHG-active thin films. Importantly, strong SHG responses were obtained without the need for a chiral center, as the inherent asymmetry of the linear alkyl chain derivatives was sufficient to drive self-organization into non-centrosymmetric materials. These results demonstrate that asymmetric pyrimidine-based architectures combining π-conjugation and controlled supramolecular organization are promising candidates for nonlinear optical applications such as photonic devices, multiphoton imaging, and optical data storage.

## 1. Introduction

In recent years, pyrimidines have gained increasing importance in the development of materials for nonlinear optics due to their unique electronic structure and their ability to be integrated into complex molecular architectures. In particular, their use as a multipolar core has proven to be particularly relevant for the design of systems with efficient NLO responses [1,2,3,4,5,6,7,8]. Pyrimidine is a six-membered aromatic heterocycle containing two nitrogen atoms at positions 1 and 3. This structural arrangement imparts several key features to the heterocycle: (i) a reduced electron density relative to its benzene counterpart arising from the electron-withdrawing nature of the nitrogens; (ii) high planarity and rigidity which favor π delocalization and (iii) selective reactivity at the 4 and 6 positions, offering compatibility with cross-coupling strategies such as the Sonogashira reaction for the introduction of π-conjugated arms in a symmetric or asymmetric architectures. Pyrimidine is well known as a key motif in biological systems (nitrogenous bases of DNA/RNA, pharmaceutical derivatives), but it has also found its place in materials science, notably as a building block for functional liquid crystals. The intrinsic features of this core, i.e., structural rigidity, planarity, moderate polarity, and varied substitution possibilities, make it highly suitable for forming smectic, nematic, or columnar liquid crystal phases. Unlike other aromatic systems such as benzenes or terphenyls, pyrimidines allow for the lowering of the melting and mesomorphic transition temperatures, thus reducing the energetic constraints associated with the formation of organized phases. Thanks to these properties, pyrimidine can act as an acceptor center in conjugated systems. Depending on the nature of the grafted groups, it can form D–π–A–π–D architectures (A = acceptor, D = donor). In a D–π–A–π–D architecture, pyrimidine often plays the role of a central π-acceptor (A), flanked by donor-type conjugated arms (D). Symmetry can be readily disrupted through asymmetric functionalization of the pyrimidine, promoting the molecular non-centrosymmetry required for second-order nonlinear optical effects, while also enhancing thermal and optical stability [9]. The first studies on mesogenic pyrimidines focused on linear or discotic structures, where pyrimidine is often enriched with alkyl or alkoxy side chains to modulate fluidity and thermal stability. For example, Lai’s team [10] studied a series of pyrimidines substituted with three phenyl groups. Two of them were functionalized with octyl chains at position 3,4,5, and the third was functionalized with different groups (F, Cl, Br, I, NHSO_2_CF_3_, OCH_3_, CH_3_, and C_2_H_5_) at position 3 or 4. This study reveals that substitutions in position 4 favor higher phase transition temperatures compared to substitutions at position 3. Moreover, these studies also highlighted the importance of π–π interactions and electronic effects on the formation of columnar lamellar phases (Col_L_), typical of discotic molecules stacked in columns. However, beyond linear and discotic systems, recent research on pyrimidine-based bent-core structures has emerged. Rahman et al. [11] were among the first to study pyrimidine derivatives with bent cores integrating photocommutable azobenzene units, opening a new research path combining the mesomorphic properties of pyrimidine-based structures with photoreactive functionalities. These compounds, built around 2,6-pyrimidinediol as the central core, are decorated with azobenzene units terminated by polymerizable double bonds. Compounds with long side chains (*n* = 3–6) show stable banana B6 phases at room temperature, while short-chain homologs remain crystalline (*n* = 1–2). The presence of terminal double bonds, in addition to opening the way to polymerization reactions, does not compromise the stability of the phases, which is largely governed by the length of the carbon chains and the overall bent architecture.

**Scheme 1:**
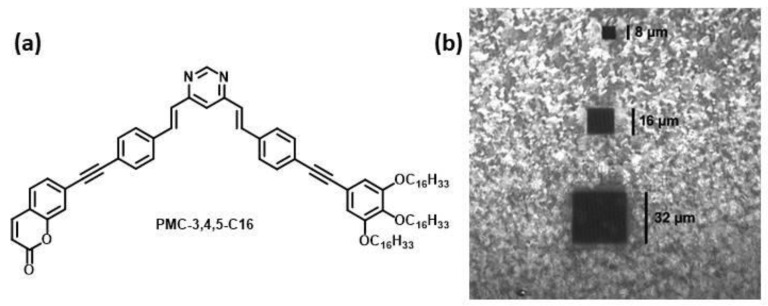
(**a**) Chemical structure of the highly asymmetric pyrimidine derivative **PMC-3,4,5-C16**, used as a reference, carrying a coumarin fragment on one side and C16 carbon chains on the other side in position 3,4,5; (**b**) optical photomicrograph recorded in the liquid crystalline phase with an SHG filter on which optical information was encrypted after three photons absorption under 1030 nm laser irradiation [12].

More recently, an asymmetric pyrimidine core with an alkynyl-styryl coumarin moiety on one side and an alkynyl-styryl hexadecyloxy moiety on the other has been designed (Scheme 1a) [12]. The coumarin fragments can undergo photocyclization under multiphoton absorption. This fragment was introduced in the molecular architecture to be able to locally disturb, under laser irradiation, the SHG signal of the liquid crystalline matrix for 3D optical data storage purposes [13,14]. This molecule can self-organize into an enantiotropic non-centrosymmetric smectic C phase between 120 and 140 °C, exhibiting strong second-harmonic generation (SHG) activity. Irradiation in the liquid crystalline phase enables rapid encoding of information by disrupting molecular organization into a non-SHG active isotropic phase after multiphoton absorption (Scheme 1b). Such dissymmetric multipolar molecules able to self-organize into SHG-active thin films hold great promise for 3D optical data storage, as they enable pinpointed multiphoton writing and pinpointed SHG reading. Additionally, SHG active materials are of great interest for a wide range of applications, including nonlinear microscopy for biomedical imaging, ultrafast photonics, optical communication, quantum information processing, frequency conversion in lasers, and surface/interface characterization in materials science [15,16].

**Figure 1:**
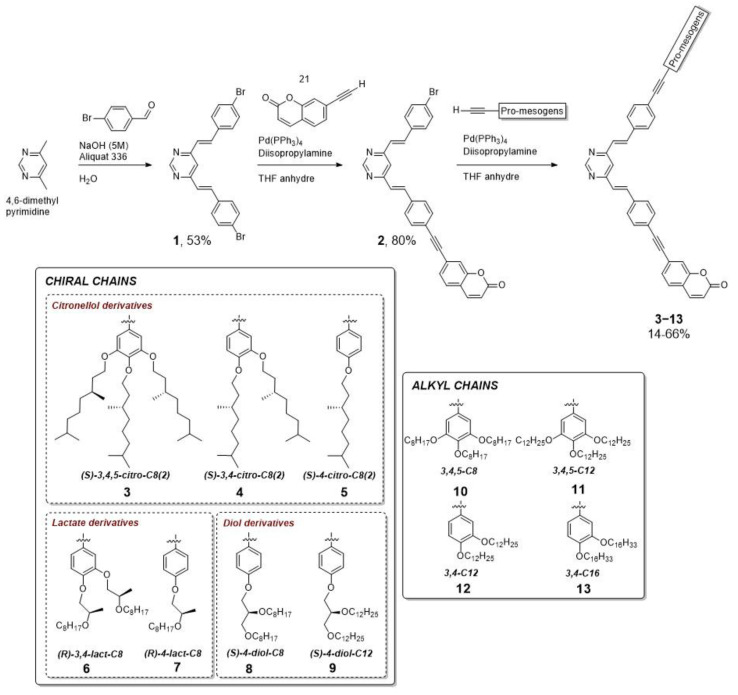
General synthesis route of the various dissymmetric pyrimidine derivatives together with the chemical structures of the 11 pro-mesogenic fragments used to functionalize the core.

Within this context, we have designed a large new series of asymmetric molecules with a pyrimidine core functionalized by two donor-conjugated units carrying a coumarin unit and a pro-mesogen unit (Figure 1). The coumarin fragment was initially introduced in molecule **PMC-3,4,5-C16** to enable a reversible writing–erasing functionality, but this behavior was not observed. Nonetheless, the design retained this feature due to its key role in tuning molecular polarity and electronic distribution. Various linear or chiral carbon chains have been introduced in various positions in order to evaluate the impact of the number and the position of the long carbon chains on the thermal behavior and on the linear and nonlinear optical properties. Compounds **3–5** correspond to the citronellol series, in which chiral citronellol-derived side chains were introduced at different substitution patterns of the aromatic ring (4, 3,4, or 3,4,5 positions). These derivatives were designed to probe the influence of bulky, flexible, and stereogenic terpenoid substituents on the physicochemical properties of the scaffold. Compounds **6–7** represent the lactate-derived series. In these molecules, a chiral lactate moiety carrying a C8 alkyl chain was attached at either the 4 or 3,4 positions of the aromatic system. This structural motif introduces both chirality and a polar ester functionality. Compounds **8–9** are diol derivatives incorporating terminal diols tethered through linear C8 or C12 carbon chains at the para position of the aromatic ring. Finally, compounds **10–13** comprise the series of simple alkyl-substituted analogs. These structures, bearing linear hydrocarbon chains of variable lengths (C8, C12, or C16) and introduced at the 3,4 or 3,4,5 positions, closely resemble the reference compound. The thermal properties of all these compounds were investigated using a combination of polarized light microscopy (POM), differential scanning calorimetry (DSC), and small-angle X-ray scattering (SAXS). In addition, their linear one-photon absorption (1PA), nonlinear two-photon absorption (2PA) in solution, and solid-state SHG properties were examined. The influence of the substitution pattern on both the thermal and optical behaviors is discussed.

## 2. Results and Discussion

### 2.1. Syntheses

The synthesis of the derivatives **3–13** was accomplished through a concise three-step strategy. First, 4,6-dimethylpyrimidine is functionalized by a Knoëvenagel reaction with 4-bromobenzaldehyde under strongly basic conditions (NaOH 5 M) (Figure 1). This reaction leads to the formation of intermediate **1**, characterized by the presence of a styryl connector on the pyrimidine core, with a yield of 53%. Next, compound **1** undergoes first Sonogashira coupling with a coumarin derivative which carries a terminal alkyne function. The product was obtained with a good yield of 80%. Compound **2** precipitates readily in common organic solvents, allowing convenient recovery by filtration. However, its limited solubility hindered complete characterization and it was directly used in the subsequent step without further purification. The formation of compound **2** was confirmed by IR spectroscopy, notably by the strong ν(C=O) vibration of the lactone at 1735 cm^−1^, and by the presence of the pyrimidine core, evidenced by two intense bands at 1581 cm^−1^ and 1508 cm^−1^ corresponding to the ν(C=N) and ν(C=C) vibrations of the pyrimidine ring (see Supplementary Materials). To finalize the synthesis of this new series of disubstituted pyrimidines, a second Sonogashira coupling is performed to graft a pro-mesogenic group onto the second brominated site of intermediate **2**. Fortunately, compound **2** gradually dissolves as the reaction progresses, allowing for the formation of the expected products without any particular difficulty. All eleven synthesized compounds **3–13** have been characterized using several techniques such as ^1^H and ^13^C NMR. These analyses allowed for the conformation of the chemical structure of the products by identifying the characteristic signals of the aromatic core, alkyl chains, and alkyne bonds. The NMR spectra of all the compounds exhibit a distinctive coupling constant of 16 Hz between the vinylic protons, clearly indicating a trans configuration around the double bonds. Moreover, elemental analyses were performed to verify the purity of each of the final compounds, **3–13**, thus confirming the successful formation of the products. The yields of this last synthesis step vary between 14 and 66%, highlighting a variable efficiency depending on the nature of the pro-mesogen groups introduced. This variability can be attributed to the differences in solubility of the reaction intermediates, thus influencing the conditions of the last Sonogashira coupling. The complete details of the experimental protocols associated with each compound as well as the complete characteristics are available in the Supplementary Materials.

The preparation of pro-mesogen precursors is based on a three-step synthesis route, aiming to functionalize the phenyl nucleus with alkoxy chains and an alkyne group that was used to perform the Sonogashira coupling. The first step consists of an alkylation reaction on a halogenophenol containing one or more alcohol functions in positions 3,4,5; 3,4; or 4, in the presence of an alkyl halide [17,18,19,20]. Different carbon chains have been considered for this new family of pro-mesogens to favor the emergence of liquid crystal phases, notably the use of branched or linear carbon chains [21,22,23]. This functionalization with alkoxy chains thus increases the solubility of the final compounds. The synthesis of chiral carbon chain precursors for the functionalization of different phenols is detailed in the Supplementary Materials. A Sonogashira coupling is then performed to introduce a trimethylsilyl-acetylene group into the aromatic cycle [24,25]. The last step is a selective deprotection performed using potassium fluoride (KF), [26] allowing for the receipt of a true alkyne function, necessary for subsequent Sonogashira coupling reactions. The structures of the eleven pro-mesogens synthesized are presented in Figure 1 and full synthetic details and characterizations can be found in the Supplementary Materials.

### 2.2. Thermal Properties

The thermal properties of this new series of asymmetric pyrimidines have been studied by polarized light microscopy (POM), differential scanning calorimetry (DSC), and small-angle X-ray scattering (SAXS). All the results are gathered in Table 1 and recorded POM images, DSC traces, and SAXS patterns can be found in the Supplementary Materials.

Except for compounds **7 ((*R*)-4-lact-C8)** and **13 (3,4-C16)**, all of the other compounds were found to be non-mesomorphic. Rather, they displayed crystal-to-crystal or crystal-to-isotropic phase transitions. The comparative study of these compounds highlights two key factors influencing the thermal behavior of the different compounds: the position of the substituents (3,4 or 3,4,5) and the length of the alkyl chains (C8, C12, C16) (Table 1). Regarding the position of the substituents, it is observed that the compounds functionalized in position 3,4,5 generally exhibit lower transition temperatures to the isotropic state than their homologues functionalized in position 3,4. For example, compound **11** presents an isotropic phase at 140 °C while compound **12** does not reach its isotropic phase until 200 °C. This trend mirrors that observed by the series of compounds bearing chiral carbon chains, i.e., the more substituents there are on the terminal phenyls, the lower the transition temperatures to the isotropic phase. Regarding the length of the chain, the progressive elongation from C8 to C12 and then to C16 also reveals a similar trend to the previous series: the melting temperatures decrease with the increase in the chain. For example, compound **10** with C8 chains in the 3,4,5 position shows an isotropic phase at 184 °C while the isotropic phase of compound **PMC-3,4,5-C16** with linear C16 chains in the 3,4,5 position is at 127 °C. The decrease in the isotropization temperature with increasing alkyl chain length or with the introduction of alkyl substituents is mainly attributed to the increased molecular flexibility and conformational freedom introduced by the alkyl segments. As the alkyl chains become longer, the proportion of the flexible part of the molecule increases relative to the rigid mesogenic core. This results in less efficient molecular packing and weaker intermolecular van der Waals and π–π interactions within the mesophase. Consequently, this leads to a lower transition temperature to the isotropic phase [27].

**Figure 2:**
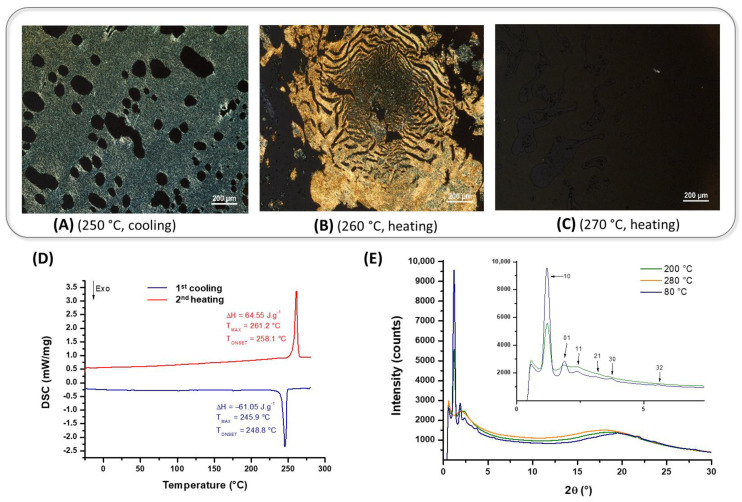
POM images of compound **7 ((*R*)-4-lact-C8)**: (**A**) at 250 °C during the first cooling (**B**) at 260 °C during the first heating, and (**C**) at 270 °C during the first heating. (**D**) DSC curve of compound **7** (heating rate: 10 °C/min), (**E**) SAXS diffraction diagram recorded on compound **7** at 80 °C (in blue), at 200 °C (in green), and at 280 °C (in orange).

Compound **7 (*R*)-4-lact-C8** presents thermal behavior characterized by a single reversible thermal transition centered at 253 °C (Figure 2D). However, an in-depth study of the SAXS patterns (Figure 2E) reveals that, below this temperature, the compound adopts a highly organized liquid crystal phase (blue and green curves), while beyond this temperature, it passes to an isotropic phase (orange curve). This transition between an isotropic phase and a liquid crystal phase is also confirmed by observation under polarized optical microscopy, which shows a passage from a soft birefringent solid to an amorphous liquid state (Figure 2A–C). This compound is therefore liquid crystal over the entire analysis range up to its isotropic phase observed at approximately 260 °C.

The analysis of the peaks of the diffraction diagram at 80 °C (blue) suggests that the observed liquid crystal phase adopts an oblique columnar organization (Col_o_) with asymmetric unit cell parameters (a ≠ b) and an angle β = 100°. To ensure non-centrosymmetry at the supramolecular level and thus explain the observed SHG signal (*vide infra*), it is necessary that all dipoles are aligned in the same direction, thus implying a ferroelectric columnar phase Col_OF_, where «F» stands for ferroelectric. Table 2 reports the values of 2θ at 80 °C, as well as the measured and theoretical interplanar distances corresponding to the observed liquid crystal phase.

**Figure 3:**
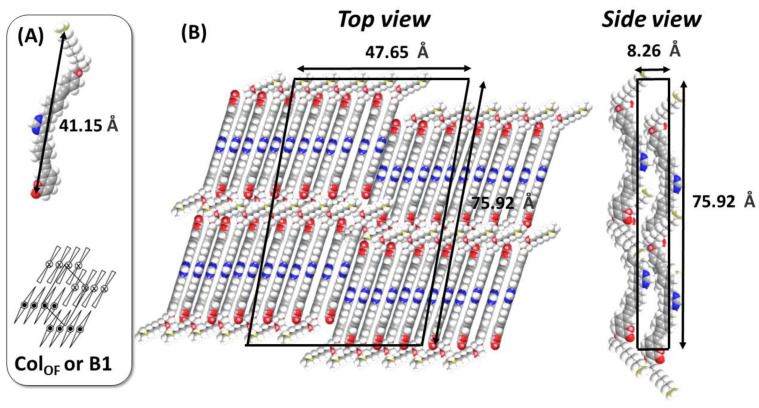
(**A**) Optimized structure of compound **7 ((*R*)-4-lact-C8)** and proposed organization in a phase of type Col_OF_ or B1 and (**B**) proposed structural model of the organization of the molecules in a phase Col_OF_ or B1 with the molecules head-to-tail (molecular geometry optimization: Chem3D 23.1.1 (**A**), scaled model illustration: Photoshop 6.0 (**B**)) (red balls correspond to the oxygen atoms of the coumarin fragment and the blue balls correspond to the nitrogen atoms of the pyrimidine).

The surface of the unit cell and the volume of the molecule have been estimated from the following relationships. The surface of the unit cell was determined with the formula L_base_ × L_height_, while the molecular volume was estimated using the relation V_molecule_ = M/0.6022 [28]. From the stacking distance of the aromatic nuclei measured at 80 °C by SAXS (4.09 Å), we estimated that approximately 12 molecules are present on the small dimension of the unit cell (b). The Col_OF_ unit cell modeling indicates a total of approximately 24 molecules per unit cell, thus allowing us to estimate the distance between the molecules in the stacking direction (c) at approximately 8.26 Å (V_cell_/Cell area). It is essential to note that this molecular model is based on the hypothesis that the indexing in an oblique columnar unit cell is correct. This proposal is based on the analysis of the diffraction data, but it could require additional investigations, notably through in-depth studies by molecular modeling, to confirm with precision the supramolecular arrangement adopted by this compound. Due to the bending of the molecule, the adopted architecture could correspond to a B1 phase, characterized by an arrangement of the molecules in blocks, themselves disposed in an oblique columnar network (Figure 3B) [29]. A head-to-tail (HT) arrangement of the molecules in the blocks allows for a better micro-segregation of the polar part and of the aliphatic chains.

**Figure 4:**
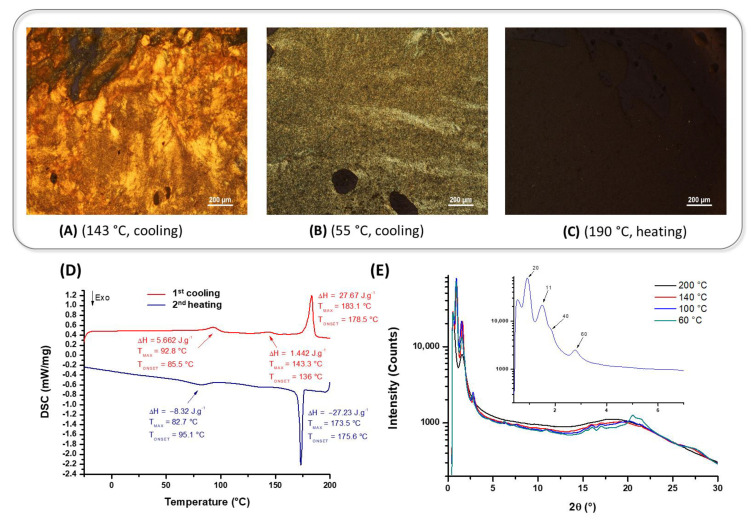
POM images of compound **13 (3,4-C16)** (**A**) at 143 °C during the first cooling, (**B**) at 55 °C during the first cooling, and (**C**) at 190 °C during the first heating. (**D**) DSC curve of compound **13** (heating rate: 10 °C/min), (**E**) SAXS diffraction diagrams recorded on compound **13** at 200 °C (in black), at 140 °C (in red), at 100 °C (in blue), and at 60 °C (in green) during cooling.

The DSC analysis of compound **13** revealed several thermal transitions during the heating/cooling cycles (Figure 4D). During the first cooling, a marked exothermic transition is observed at 173.5 °C, translating the progressive formation of a more ordered phase. This intermediate phase persists over a wide temperature range, approximately 90 °C, before a second, less-energetic transition which appears at 83 °C. The latter could correspond to the formation of a crystalline phase. The study by small-angle X-ray scattering (SAXS) allowed for a refinement of the understanding of the transitions observed during cooling (Figure 4E). At a high temperature (200 °C), the absence of diffraction peaks confirms that the compound is in an isotropic state, without supramolecular order. On the other hand, below 173.5 °C (red and blue curves), well-defined signals appear at small angles, while more diffuse signals emerge at short distances and intensify as the temperature decreases (from 140 °C to 100 °C). These observations are characteristic of a highly structured liquid crystal phase. Below 83 °C, a refined SAXS signal is observed over the entire 2θ range, suggesting that there is a well-defined crystalline organization. The interpretation of these transitions in SAXS and DSC is confirmed by the observation of the compound between crossed polarizers. At 190 °C, the compound appears non-birefringent, confirming the isotropic state (Figure 4C). On the other hand, at 143 °C, a highly birefringent and deformable texture under stress is observed, confirming the presence of a structured liquid crystal phase (Figure 4A). During heating, three distinct endothermic transitions are recorded. At 98 °C, the first weakly energetic transition is noted, analogous to that observed during cooling at 83 °C, probably indicating a passage from a crystalline state to a liquid crystal phase. At 143 °C, a second transition peak is detected, suggesting a structural reorganization within the liquid crystal phase. However, this transition does not significantly affect the SAXS and POM images, which leads us to think that it is a minor adjustment of the molecular order. Finally, at 183 °C, a more energetic transition is observed, corresponding to the melting of the liquid crystal phase to the isotropic state. The high enthalpy associated with this transition indicates a complete rupture of the supramolecular interactions that stabilized the ordered liquid crystal phase. The combined analyses of DSC, SAXS, and POM highlight an enantiotropic highly ordered liquid crystal phase for compound **13**. This phase is stable over a wide temperature range, approximately 90 °C (from 173.5 °C to 83 °C during cooling and from 93 °C to 183 °C during heating).

The analysis of the diffraction peaks recorded at 100 °C (blue curve) indicates that this phase adopts a rectangular columnar organization (Col_r_), characterized by asymmetric unit cell parameters (a ≠ b) and a right angle (β = 90°). To ensure non-centrosymmetry at the supramolecular level and thus guarantee a good SHG signal, it is essential that all molecular dipoles are aligned in the same direction. This type of organization then leads to the emergence of a ferroelectric Col_rF_ phase, where « F » stands for ferroelectric. This hypothesis is based on an analogy with the Col_OF_ phase previously described for compound **7**, which presents a similar structure. Table 3 presents the values of 2θ measured at 100 °C recorded on cooling, as well as the theoretical and measured interplanar distances associated with this ordered liquid crystal phase.

**Figure 5:**
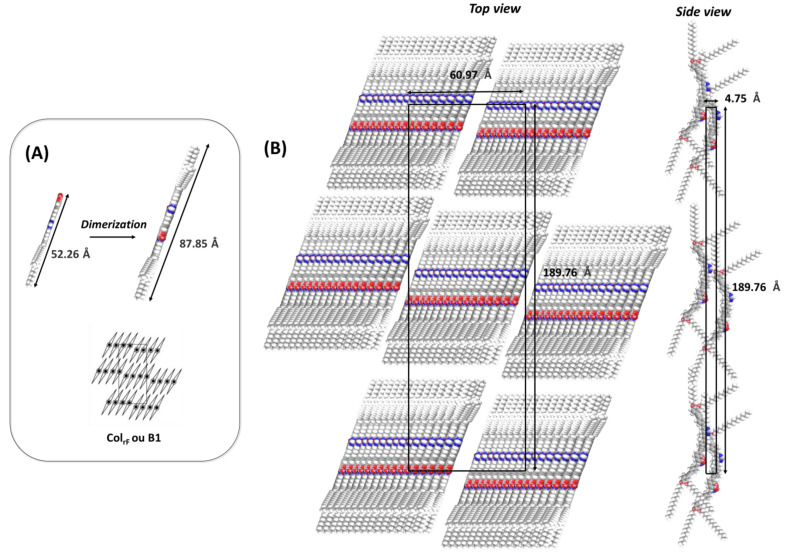
(**A**) Optimized structure of compound **13 (3,4-C16)** and its dimer and proposed organization in a phase of type Col_rF_ or B1; (**B**) structural model of the organization of the molecules in phase Col_rF_ or B1 with the molecules head-to-tail (molecular geometry optimization: Chem3D 23.1.1 (**A**), scaled model illustration: Photoshop 6.0 (**B**)) (red balls correspond to the oxygen atoms of the coumarin fragment and the blue balls correspond to the nitrogen atoms of the pyrimidine).

By applying the same methodology previously used for compound **7**, we estimated the surface of the unit cell and the volume of a molecule. The SAXS analysis carried out at 140 °C revealed a stacking distance of the aromatic nuclei of 4.12 Å, which allowed us to evaluate approximately 16 molecules occupying the small dimension of the unit cell (b). The modeling of the Col_rF_ structure allowed us to estimate approximately 32 molecules per unit cell, leading to an estimation of the intermolecular distance along the stacking axis of the molecules at approximately 4.75 Å (V_cell_/Cell area). A molecular organization model is then proposed in Figure 5. It is important to note that this hypothesis is based on the indexing in a rectangular columnar unit cell and is based on the diffraction data. Due to the curvature of the molecule, the adopted architecture could also be assimilated to a B1 phase (Figure 5A), characterized by a molecular arrangement in blocks, these being organized in a rectangular columnar network with a block positioned at each vertex and a central block (Figure 5B) [24]. Approximately three molecules disposed along their long axes would be necessary to fill the large dimension (a) of the rectangular unit cell, but the molecular length (52.26 Å) does not allow for it to reach an optimal filling. To resolve this structural constraint, we propose the formation of head-to-tail (HT) dimers (Figure 5A), which allow for an increase in the molecular length of a block to 87.85 Å, a value sufficient to adjust perfectly to the rectangular unit cell. These blocks of 16 HT molecules would then be disposed in a rectangular network where all the dipole moments are aligned in the same direction according to c, thus guaranteeing the non-centrosymmetry of the thin films, an essential criterion that would explain the observed SHG signal (*vide infra*).

Only compounds **7** and **13** exhibited mesomorphic behavior. In contrast to the reference compound **PMC-3,4,5-C16**, which forms a smectic mesophase, these two derivatives with a reduced number of long alkyl chains self-assemble into a highly ordered and viscous columnar mesophase. The study reveals that a high number of long alkyl chains is required for the emergence of liquid crystalline properties.

### 2.3. Linear One-Photon Absorption (1PA)

**Figure 6:**
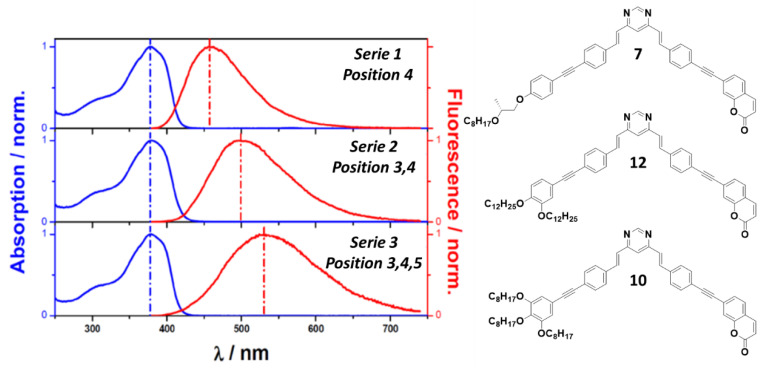
Examples of normalized absorption and fluorescence spectra in THF of three compounds with a different number of -OR chains (dashed lines correspond to the absorption and emission maxima).

First, the one-photon absorption properties on this series of compounds were carried out. For practical comparison, the 12 studied compounds, including **PMC-3,4,5-C16**, will be classified into three distinct series depending on the number of alkoxy substituents (-OR) present on the terminal phenyl group (series 1: position 4, series 2: position 3,4 and series 3: position 3,4,5). Figure 6 represents the normalized absorption and fluorescence spectra typical of each family of derivatives in THF. The optical properties of the studied compounds are presented in the table below (Table 4). The analysis of these data allows for a better understanding of the influence of structural modifications (length, nature, and positions of carbon chains) on the absorption, emission, and deexcitation characteristics of these molecules. The properties of compounds **5** and **13** could not be fully studied due to the low yield obtained during their synthesis. However, given the similar behavior of their analogs possessing -OR substituents in the same positions, it is reasonable to assume that these two compounds would follow the same trends and conclusions.

All derivatives globally present the same absorption spectrum regardless of the number of -OR substituents. As shown in Figure 6, the red energy part of each spectrum consists of a structureless band located in the 280–420 nm range with a maximum at ~380 nm. Note that a weak shoulder can also be observed at the blue edge of this main absorption band. All the derivatives display high molar extinction coefficients at λ_max_ with values between 80 and 117 × 10^3^ M^−1^ cm^−1^. This is a clear indication of the presence of strongly allowed π−π* type transitions. Such low energy transitions presumably imply a charge transfer (CT) character from the alkoxy substituted phenyl subunit to the pyrimidine core. It should be noted that the position of the absorption spectra remain insensitive to the number of -OR groups, whereas the corresponding fluorescence spectra undergo a significant band red shift ongoing from series 1 to series 3 (see Figure 6). This spectral effect should be attributed to the progressive energy stabilization of the relaxed emitting state whose CT character is gradually enhanced due to the introduction of additional alkoxy donor groups. The progressive enhancement of the emitting state CT character is also illustrated when considering the fluorescence bathochromic shift which clearly increases ongoing from series 1 to 3, whereas the corresponding absorption spectra remain globally invariant (see Figure 6). Such a gradual increase in the Stokes shift evidences an amplified S_0_-S_1_ electronic relaxation, presumably due to a significant enhancement of the relaxed S_1_ state dipole moment leading to a more significant polarity-induced stabilization of the emitting state in THF (medium polar solvent). As indicated in Table 4, the radiative rate constants (k_r_) remain globally constant within each series which confirms the electronic equivalency of each emitting state independently to the nature of the alkyl chain. It is also worth noting that the number of -OR substituents does not affect the dyes’ emissivity for series 1 and 2 which exhibit high fluorescence quantum yields (Φ_fluo_ > 0.7) but leads to a 2-fold decrease in Φ*_fluo_* for compounds of series 3. In this latter case, the decrease in the emissive singlet state’s energy should open additional non-radiative deactivation channels which compete with the radiative deactivate processes. This effect is nicely confirmed by comparing the ratios of the non-radiative (k_nr_) and radiative rate constants (k_r_) of series 3 with those of the other series. As indicated in Table 4, these k_nr_-to-k_r_ ratios are globally 6-fold larger than those measured for series 1 and 2. The analysis of the optical and photophysical parameters of the studied compounds then highlights the following trends:

- Series 1 is the most luminescent, with higher fluorescence quantum yields and lower non-radiative losses.
- Series 2 maintains good optical properties with fluorescence yields slightly lower than series 1, although the non-radiative constant is higher than series 1.
- Series 3 presents significant losses through non-radiative conversion, strongly reducing its luminescent efficiency.

These results underscore the importance of the chemical structure and the position of the carbon chains on the terminal phenyl groups in modulating the optical properties.

### 2.4. Nonlinear Two-Photon Absorption

**Figure 7:**
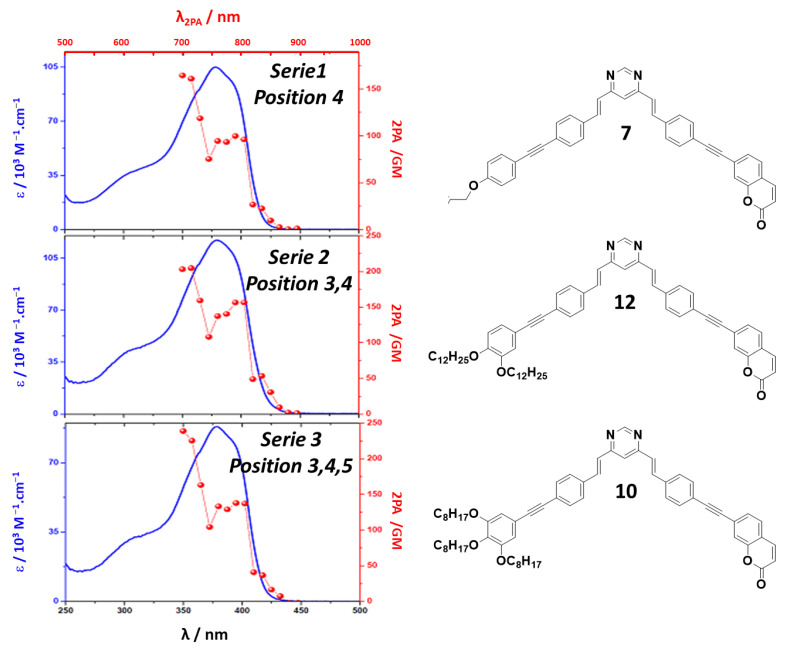
1PA (blue line) and 2PA spectra (red line) of the series in THF (2PA spectra are plotted against half of the excitation wavelength).

The molecules of this series present great variability in terms of their emissivity with Φ_fluo_, ranging from 0.36 to 0.82 in THF, these fluorescence quantum yields were sufficient to measure the two-photon absorption cross-sections (δ_λ_) and the corresponding spectra using the two-photon excited fluorescence (2PEF) technique. As for our analysis of the 1PA spectra, the alkyl chains of the -OR substituents have no influence on the 2PA properties, only the number of substituents matters. Figure 7 presents the 1PA and 2PA spectra of three compounds belonging, respectively, to the mono-, di-, and trisubstituted series. Linear absorption is represented by the blue curve on each spectrum and highlights the π-π* electronic transitions that occur within each molecular structure, and two-photon absorption is represented by the red curve. In the same way as for the symmetric tetrasubstituted bipyrimidines, [30] the 1PA and 2PA spectra of these compounds do not have the same appearance. The 2PA spectra show two distinct bands, a band of lower energy of average intensity in the red between 750 and 800 nm and a band of higher energy around 700 nm in the blue with stronger 2PA cross-sections. Note that the ratio between these two δ remains globally constant in each series, confirming the low electronic and structural variability of the molecules when changing the alkyl chain. Another trend that emerges by comparing the families of molecules is the gradual increase in δ_MAX_ with the number of -OR substituents. Thus, from mono- to trisubstituted derivatives, δ_MAX_ increases on average by 21% (148 to 179 GM, average value of each series). This increase is correlated with the increase in the polarity of the excited states by reinforcing the donor character through the addition of -OR substituents. This trend is also illustrated through the bathochromic shift observed on the fluorescence of these same derivatives. Finally, as these systems have the same number of π electrons participating in the nonlinear response, it is clear that the trisubstituted derivatives present the best performance in two-photon absorption. The table below (Table 5) presents the results of the two-photon absorption measurements for the series of compounds with a pyrimidine core.

The compounds of series 1 all present a maximum two-photon absorption at 700 nm, which indicates a certain stability in their electronic structure despite variations in the nature of the carbon chains at the periphery of the phenyls. Within series 1, compound **7**, has the highest value of 164 GM and the absorption cross-section varies between 123 and 164 GM in this series, which corresponds to a moderate efficiency in two-photon absorption. A slight modification of the nonlinear response due to the nature of the carbon chains is observed here. Series 3 is the one that presents the best performance in two-photon absorption. Compounds **3** and **10** reach a cross-section of 238 GM, which is the highest value observed in this study. These results suggest that the structure of this series is functionalized by carbon chains in position 3,4,5 and favors good multiphoton absorption. In series 2, two of the three compounds (**4** and **6**) show a slight shift in the maximum absorption towards 715 nm, while compound **12** retains a maximum absorption at 700 nm like for series 1. This shift towards longer wavelengths can be attributed to a better electron delocalization in the molecule due to the addition of a chain in position 3,4. The values of δ_2PA_ in this series are higher than those of series 1, with a range from 143 to 205 GM. Compound **12** shows the best cross-section (205 GM), indicating a notable improvement in two-photon absorption properties. Series 2 thus shows an intermediate behavior between series 1 and 3 in terms of NLO performances. The analysis of the two-photon absorption cross-sections of the different series highlights the impact of the number and position of the substituents. Indeed, the increase in the number of alkoxy groups seems to improve two-photon absorption, as shown by the progression of the δ_2PA_ values from series 1 to series 3. It appears that the presence of substituents in position 3,4,5 (series 3) is particularly beneficial for maximizing absorption efficiency. Series 3 stands out with its optimal performances, suggesting that these structures could be particularly suitable for applications in nonlinear optics. The two-photon absorption (2PA) properties of related pyrimidine-core compounds have previously been investigated by Dr. Robin-Le Guen’s group [31]. In this work, we present a comparative study of the linear and nonlinear optical properties of a bent-core bis(triphenylamine)–pyrimidine monomer together with a series of derived oligomers. The oligomers exhibit a pronounced cooperative effect in two-photon absorption. Specifically, the assembly of nine monomer units into a three-dimensional architecture results in a substantial enhancement of the 2PA cross-section, reaching a maximum of 5093 GM, compared to 360 GM for the isolated monomer. Notably, the cross-section of the monomer is of the same order of magnitude as those measured for series 1 with carbon chains at the 4 position, underscoring the strong two-photon absorption efficiency of this new family of asymmetric pyrimidine-core molecules.

## 3. Second-Harmonic Generation

The intensity of the SHG signal is strongly influenced not only by the electronic properties of the studied molecules, but also by their structural organization. In particular, a non-centrosymmetric arrangement of the molecules within the bulk is essential to generate a significant coherent SHG signal. The study of SHG properties carried out on the pyrimidine-core compounds allowed us to understand the influence of the nature, position, and length of the carbon chains grafted onto the terminal phenyls. The SHG images were captured for the most representative compounds of each series. For this purpose, each compound was immobilized in a liquid crystal cell with a controlled and uniform thickness of 4 μm, without surface treatment, specifically without indium-tin oxide (ITO) deposition on the glass surface (E.H.C Co, Ltd., Tokyo, Japan). Indeed, it is important to note that ITO can itself generate an SHG signal, which could interfere with the signals specific to the studied compounds. To prepare the samples, each compound was first introduced at the opening of the cells and then heated to reach the isotropic phase, thus enabling its infiltration by capillarity. Once the cells were filled, they were cooled to room temperature. The SHG spectra and images were recorded at room temperature, and additional SHG images were recorded at elevated temperatures. For this purpose, a heating plate was installed under the SHG microscope, allowing for precise regulation of the sample’s temperature. Images were obtained by epi-SHG microscopy in scanning mode with the incident laser light polarized in the horizontal direction of the images. SHG spectra were acquired in widefield mode, with the sample illuminated over an area of 650 μm × 650 μm [12]. Spectra were obtained by closing the imaging spectrometer slit to approximately 10 μm in the vertical direction and binning across the full 650 μm horizontal extent. This configuration enabled the averaging of local variations in the SHG signal across the illuminated area. To compare the intensities of the SHG signals between the different compounds, identical experimental settings were used for the detection. To account for variability in SHG response, the incident laser power (maximum 354 mW at the output of the laser) was varied using a half-wave plate polarizer combination. In this way, we avoided overloading the ICCD camera. The SHG response, which scales quadratically with the incident laser power, was then normalized to an irradiation power of 144.69 mW to allow for a comparison of the relative SHG response for all compounds. All measurements were repeated twice and the average response is shown.

### 3.1. Citronellol-Derived Series

**Figure 8:**
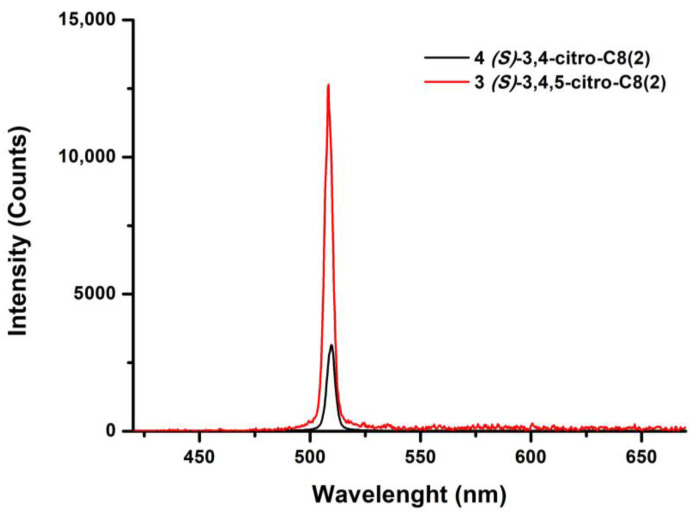
SHG spectra of the compounds of the citronellol-derived series normalized for an irradiation power after the camera of 144.69 mW at room temperature.

Compound **5** decomposed before reaching the isotropic phase and therefore could not be incorporated into the LC cells. Consequently, Figure 8 shows only the SHG spectra of compounds **3** and **4**. The highest SHG intensity is observed for compound **3**, **(S)-3,4,5-citro-C8(2)**, indicating that this substitution pattern enables an optimal alignment of the molecular dipoles, leading to non-centrosymmetry.

**Figure 9:**
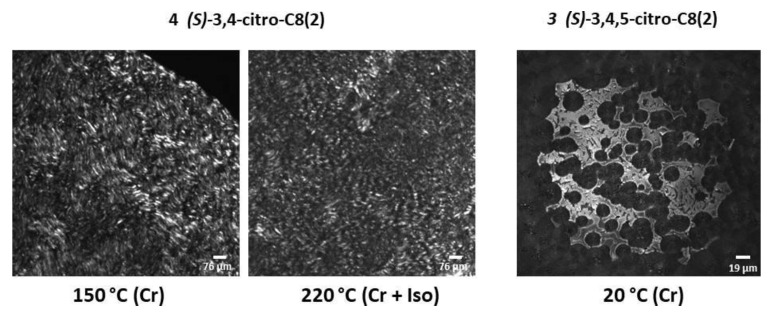
SHG images of compound **4 (*S*)-3,4-citro-C8(2)** at 150 °C with an input irradiation power of 246 mW and an irradiation power after the 20× objective of 4.07 mW (**on the left**) and at 220 °C with an input irradiation power of 246 mW and an irradiation power after the 20x objective of 4.07 mW (**on the right**); SHG image of the compound **3 (*S*)-3,4,5-citro-C8(2)** at room temperature with an input irradiation power of 246 mW and an irradiation power after the 20x objective of 2.88 mW.

To characterize the local structure, scanning SHG microscopy was performed for compounds **3** and **4** (Figure 9). Acquisition settings were optimized for the different images. For compound **4**, two images were captured on heating at different temperatures, thus allowing us to evaluate the thermal impact on the organization of the molecular films and their SHG response. The image taken at 150 °C shows the crystalline phase of the compound. The SHG image reveals microcrystalline domains with a relatively homogeneous response, indicating a well-defined non-centrosymmetric molecular organization in the thin film. A second image of the same compound was taken at 220 °C (at the melting point), revealing a marked increase in signal heterogeneity, with more numerous and more extensive non-SHG active zones. At identical irradiation power (4.07 mW), this evolution indicates an organizational transition from a structured crystalline state to a liquid isotropic state, characterized by the appearance of disorganized non-SHG active zones. The last image corresponds to compound **3** at room temperature after cooling from the melt. Compared to compound **4**, the SHG response here is much more homogeneous, with highly active SHG domains. This difference could be attributed to the presence of an additional citronellol-derived chain at position 5, which modifies the intermolecular interactions thus influencing the self-assembly of the molecules within the film.

### 3.2. Lactate-Derived Series

**Figure 10:**
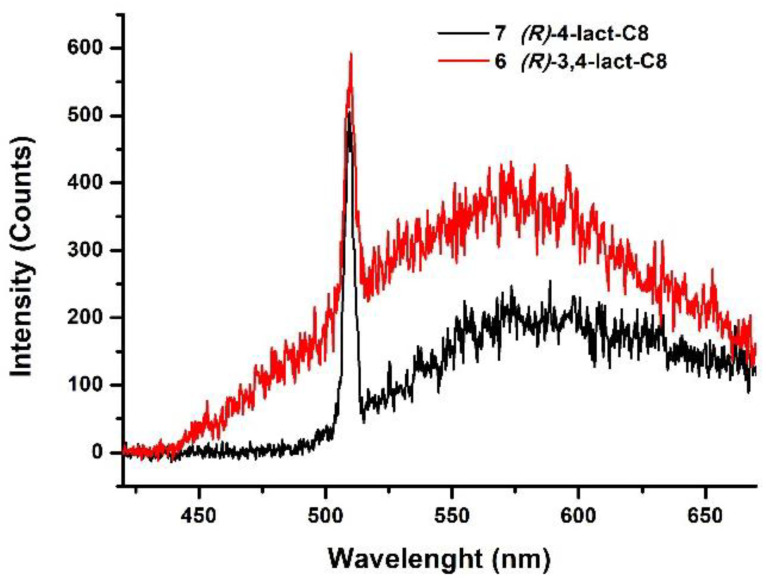
SHG spectra of compounds **6** and **7** of the lactate-derived series normalized for an irradiation power after the camera of 144.69 mW at room temperature.

Figure 10 presents the SHG spectra obtained for compounds **6 (*R*)-3,4-lact-C8** and **7 (*R*)-4-lact-C8** functionalized with lactate derivatives that differ in the position and number of alkyl chains on the terminal phenyl. A peak at 510 nm corresponding to the SHG signal is observed for both compounds. The two compounds exhibit nearly identical and weak SHG intensities. Unlike the corresponding compounds functionalized by chiral chains, a broad multiphoton fluorescence band centered at 575 nm can be attributed to the contribution of multiphoton absorption. Given the relatively weak SHG signals and the overlapping multiphoton fluorescence, no SHG images were acquired for these compounds.

### 3.3. Diol-Derived Series

**Figure 11:**
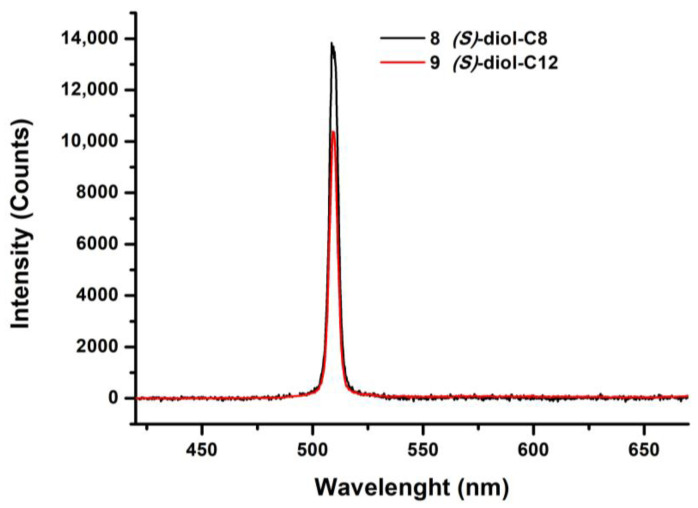
SHG spectra of compounds **8** and **9** of the diol-derived series normalized for an irradiation power after the camera of 144.69 mW at room temperature.

SHG spectra obtained for compounds **8 (*S*)-4-diol-C8** and **9 (*S*)-4-diol-C12** functionalized with diol derivatives differing only in the length of their alkyl chains are presented in Figure 11. The compounds exhibit strong SHG intensities, indicating a non-centrosymmetric bulk. Their SHG response is nearly identical, indicating that the elongation of the alkyl chain (from C8 to C12) has no significant effect on the bulk organization and the resulting SHG response.

**Figure 12:**
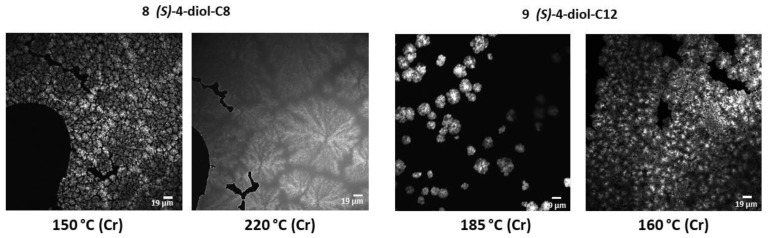
SHG images of compound **8 (*S*)-4-diol-C8** at 150 °C with an input irradiation power of 246 mW and an irradiation power after the 20x objective of 0.74 mW (**on the left**) and at 220 °C with an input irradiation power of 246 mW and an irradiation power after the 20× objective of 8.5 mW (**on the right**); SHG images of compound **9 (*S*)-4-diol-C12** at 185 °C with an input irradiation power of 246 mW and an irradiation power after the 20x objective of 6.15 mW (**on the left**) and at 160 °C with an input irradiation power of 246 mW and an irradiation power after the 20x objective of 10.16 mW (**on the right**).

The images shown below depict the SHG responses of compounds **8** and **9** obtained from thin films at various temperatures (Figure 12). The acquisition parameters were individually optimized for each image, preventing a direct comparison of SHG intensities. Accordingly, the discussion will focus solely on the molecular organization within the films rather than on the absolute intensity of the SHG signals. The first image of compound **8** was taken at 150 °C on heating in the crystalline phase. This image reveals micrometer domains presenting a moderate and heterogeneous SHG signal. Upon further heating to 220 °C, a new texture emerges, accompanied by a relatively homogeneous SHG signal across the film. Within this uniform background, large fractal-like structures exhibiting enhanced SHG intensity become apparent. These structures display motifs with a planar radial distribution characteristic of a crystalline texture, consistent with the POM observations within the same temperature range. Regions of reduced SHG activity are observed at the grain boundaries separating the crystalline domains. For compound **9,** with the longest carbon chains, the image taken at 185 °C on cooling from the isotropic phase shows the onset of crystallization characterized by the appearance of polycrystalline domains with intense SHG response confirming a non-centrosymmetric organization. The second image, taken at a lower temperature (160 °C), confirms the presence of this non-centrosymmetric crystalline phase with a texture similar to that observed at 185 °C, but with well-defined microdomains that cover the entire film’s surface. To summarize, for compound **8**, an increase in temperature from 150 °C to 220 °C led to a marked reorganization, passing from a dispersed microcrystalline structure to larger radial structures. For compound **9**, organization into homogeneous crystalline domains appears at 185 °C after which full crystallization takes place, which remains stable at lower temperatures.

### 3.4. Linear Chains Series

**Figure 13:**
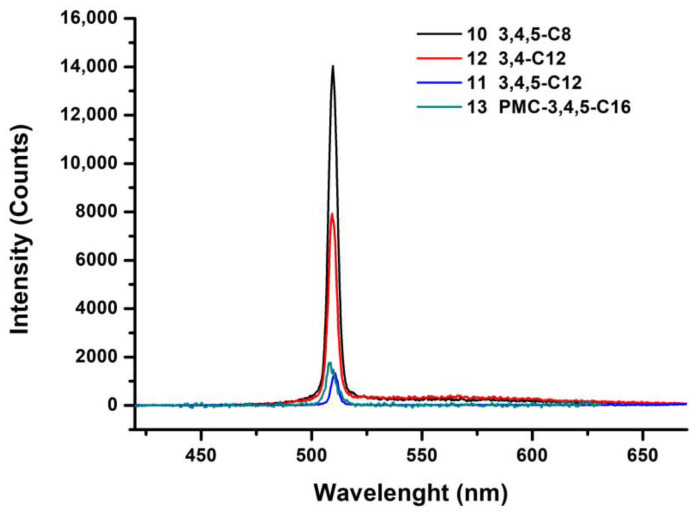
SHG spectra of the compounds of the series with linear carbon chains normalized for an irradiation power after the camera of 144.69 mW at room temperature.

SHG spectra of the series with linear carbon chains **10–13** are presented in Figure 13. The spectra reveal significant variations in SHG intensity depending on both the chain length and position of the alkyl chains. Compound **10 3,4,5-C8** presents the highest SHG intensity, with a sharp peak at 510 nm. This strong SHG response reveals an optimal non-centrosymmetric organization in this film. The SHG intensity of compound **12 3,4-C12** is attenuated by 44%, although the signal remains significant. This decrease can be attributed to a partial alteration of the non-centrosymmetric organization in the material due to the removal of a carbon chain. The spectra corresponding to compounds **11 3,4,5-C12** and **PMC-3,4,5-C16** show lower intensities than their homologues functionalized at position 3,4. In contrast to the trend observed for mesogenic phase formation, the elongation of the carbon chains grafted at the periphery of the conjugated core does not improve the SHG signal.

It is noted that for this series, functionalized by the pro-mesogens bearing the linear chains, the SHG intensities are of the same order of magnitude as those obtained in the series of compounds functionalized by chiral pro-mesogens. This shows that molecular asymmetry, even in the absence of chiral symmetry breaking, presents an alternative path for inducing non-centrosymmetry of the supramolecular state necessary for the generation of an SHG signal. In addition, it should be emphasized that the highest SHG signal in the entire series was obtained for the compound bearing an achiral side chain (**3,4,5-C8**).

**Figure 14:**
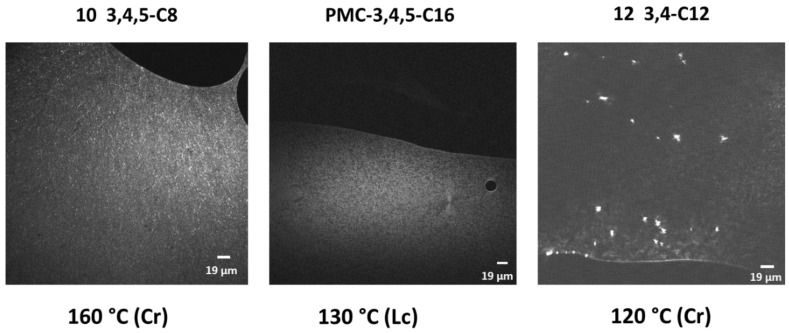
SHG image of compound **10 3,4,5-C8** at 160 °C with an input irradiation power of 246 mW and an irradiation power after the 20× objective of 6.15 mW (**on the left**); SHG image of the compound **PMC-3,4,5-C16 3,4,5-C16** at 130 °C with an input irradiation power of 246 mW, an irradiation power after the 20x objective of 23.86 mW (**in the middle**), and an SHG image of compound **12 3,4-C12** at 120 °C with an input irradiation power of 246 mW and an irradiation power after the 20× objective of 20.53 mW (**on the right**).

The images presented below illustrate the SHG response of three selected compounds of the family of linear chains (Figure 14) allowing us to evaluate the impact of the length and position of the grafted chain on the SHG response and to compare with the results obtained for the series of compounds functionalized with chiral carbon chains. All these images were recorded after cooling from the melt. The SHG image obtained from compound **10 3,4,5-C8** was taken at 160 °C in the crystalline phase and shows the presence of micrometer domains with relatively uniform SHG signals over the entire film surface. This confirms the non-centrosymmetric organization induced by the overall molecular asymmetry. The compound **PMC-3,4,5-C16** was analyzed at 130 °C in the liquid crystal phase. The obtained liquid crystalline phase shows a relatively uniform moderate SHG response and microdomain structure. Local variations in SHG intensity are likely related to differences in orientation of the microdomains. This shows that non-centrosymmetry is maintained at the supramolecular level despite the fluid character of the liquid crystal phase of the compound.

Compound **12 3,4-C12**, observed at 120 °C (crystalline phase), shows a different picture. Unlike the two other compounds, this image presents a very heterogeneous surface with local domains presenting high SHG intensity distributed throughout the otherwise moderately SHG-active thin film. This strong local intensity of the SHG signal suggests a local organization in crystalline monodomains, showing that this compound has more difficulty organizing itself than its homologues functionalized in position 3,4,5.

Comparison of the SHG spectra obtained on the two families bearing chiral and linear carbon chains (Figure 8, Figure 10, Figure 11 and Figure 13) reveals interesting structure–property relationships. All spectra show a sharp peak at the SHG wavelength. However, several interesting trends are noted. The first critical observation is that the addition of an asymmetric carbon to obtain a non-centrosymmetric thin film is not required for obtaining SHG activity. Indeed, the intensities of the SHG signals obtained for the family of compounds bearing linear chains are comparable to those obtained for the family of compounds bearing chiral carbon chains. This shows that the overall molecular asymmetry, due to the asymmetric functionalization of the pyrimidine core, is sufficient to generate non-centrosymmetry. It is also noted that the effect of the length of the alkyl chain is negligible for both families. Indeed, the transition from an octyl chain to a dodecyl chain in the diol series does not significantly modify SHG intensity like the transition from an octyl chain to a hexadecyl chain for the series with linear carbon chains, suggesting that the nonlinear response essentially depends on the position of these groups at the periphery of the pyrimidine and not on their length. The SHG images show that the SHG signal of each compound strongly depends on the temperature and the organization of the compound in the form of thin films. These results are particularly interesting because they demonstrate first that the molecular organization, modified at different temperatures, allows for an adjustment of the NLO response of the materials in a straightforward manner. Secondly, the position of the substituents plays a determining role in the organization of the molecular films, directly impacting their ability to generate an SHG signal. It then appears that positions 4 and 3,4,5 favor the best organization of the thin films for a better SHG response.

## 4. Conclusions

This work was devoted to the design, synthesis, and characterization of a new series of novel organic compounds with a disubstituted pyrimidine core. This study is part of a broader framework aimed at developing materials with optimized nonlinear optical properties, notably through the integration of a coumarin group and pro-mesogenic groups capable of promoting non-centrosymmetric supramolecular organization, essential for the appearance of NLO phenomena. The synthetic strategy is based on a Knoëvenagel condensation, followed by two Sonogashira couplings. This methodology allowed for the preparation of a pyrimidine core, functionalized first by a coumarin unit, then by different pro-mesogens bearing chiral or linear carbon chains. This modularity offered a great structural diversity, both in terms of the nature of the grafted chains and their positioning on the aromatic motif. Eleven new compounds were successfully synthesized with accurate yields, and their thermal and optical properties were thoroughly investigated. In the first family of molecules, chiral chains, derived from citronellol, methyl lactate, or (*S*)-3-chloropropane-1,2-diol were grafted to obtain the pro-mesogenic units. These modifications aimed to transfer molecular asymmetry to the supramolecular level in order to guarantee the non-centrosymmetry of the thin films required for the appearance of an SHG signal. However, the results showed that these chiral chains do not favor the appearance of fluid mesomorphic phases. Among the seven compounds synthesized with chiral chains, only compound **7 (*R*)-4-lact-C8** showed a stable liquid crystal phase presenting molecular organization in an oblique columnar lattice (Col_OF_). However, this intriguing behavior is constrained by the pronounced supramolecular organization, which significantly limits the system’s fluidity—a parameter that remains crucial for the optimal performance of NLO devices. The other compounds, notably those derived from citronellol or diol, mainly presented more classical crystalline behaviors, with crystal–crystal or crystal–isotropic transitions, without revealing interesting mesomorphic properties. A second family of pyrimidine-core molecules was therefore developed by replacing the chiral chains on the phenyls with linear carbon chains of variable lengths (C8, C12, C16) in different positions. The idea was to enhance the fluidity of the system and to favor a less rigid self-organization, more suitable for the generation of modifiable non-centrosymmetric films. This approach proved to be more fruitful: two compounds showed interesting liquid crystal transitions over a wide temperature range. Indeed, the new compound **13** functionalized by C16 chains in position 3,4 as well as the previously published compound **PMC-3,4,5-C16** with C16 chains in the 3,4,5 position both showed an interesting mesomorphic behavior with a very organized liquid crystal phase in a rectangular columnar network for the first and a smectic C phase for the second. The linear absorption, nonlinear two-photon absorption, and properties of these new compounds have been investigated and have allowed for an evaluation of the potential of this family of molecules for nonlinear optical applications, notably in relation to the molecular structure and the supramolecular organization induced by the grafted chains. The study of the 2PA spectra highlighted significant activity in nonlinear optics for certain compounds comparable to the literature. It was noted that to guarantee good cross-section values, position 4 must be functionalized. The compounds functionalized with chiral chains showed a slight increase in the 2PA signal compared to the compounds functionalized with linear carbon chains. SHG studies on thin films were carried out to evaluate the SHG efficiency of the materials in these series. Most of the compounds present a measurable SHG signal, proof of a non-centrosymmetric organization at the supramolecular scale, except for the compounds bearing the chains derived from methyl lactate, presenting only weak SHG on broad multiphoton fluorescence. The compounds bearing chiral chains derived from diol showed a more favorable organization in the solid state favored by the transfer of molecular chirality to the supramolecular scale. It should be emphasized that good SHG properties were achieved even in the absence of chirality, when using linear alkyl chains, as the molecular asymmetry proved sufficient to drive self-organization into non-centrosymmetric materials. The results obtained in this study of optical properties demonstrate that the dissymmetric compounds have real potential in nonlinear optics, but their efficiency strongly depends on the nature and position of the grafted chains and also on their ability to self-organize into non-centrosymmetric organized films. The strategy of working on asymmetric pyrimidine-core molecules proved advantageous since these compounds are synthetically more straightforward than bipyrimidines and they exhibit promising nonlinear optical properties. These results highlight the potential of these compounds in nonlinear optics, owing to their conjugated core and their ability to adopt non-centrosymmetric supramolecular organizations.

## 5. Experimental Section/Methods

Full synthetic details for the preparation of the compounds and their intermediates are given in Supplementary Materials. NMR spectra were recorded on a Bruker Avance 300 spectrometer at 300 (^1^H) and 75.5 MHz (^13^C) at room temperature using residual proton solvents as internal standards. Elemental analyses were performed by the Service de Microanalyse BioCIS, Faculté de pharmacie, Université Paris-Saclay, Orsay, France.

DSC was carried out by using an NETZSCH DSC 200 F3 instrument equipped with an intracooler. DSC traces were measured at 10 °C/min down to −25 °C.

Optical microscopy investigations were performed on a Nikon H600L polarizing microscope equipped with a Linkam “liquid crystal pro system” hotstage LTS420. The microscope was equipped with a UV irradiation source (Hg Lamp, λ = 340−380 nm) which was used for writing on the LC films. The microscope was also equipped with an Ocean Optic USB 2000 + UV–Vis-NIR spectrophotometer and a UV-2A filter cube for recording luminescence spectra under UV irradiation at 340–380 nm.

X-ray scattering experiments were performed using a XENOCS GeniX3D (Holyoke, MA, USA) low convergence copper micro source (50 W) equipped with FOX3D (Tallinn, Estonia) single reflection optics and point collimation. The patterns were collected with a Mar345 Image-Plate detector (Marresearch, Norderstedt, Germany). The samples were held in Lindeman glass capillaries (1 mm diameter). The capillaries were placed inside a Linkam HFX350-Capillary (Redhil, UK) X-Ray stage which allows measurements from −196 °C up to 350 °C with an accuracy of 0.1 °C.

The absorption measurements were carried out with a Perkin Elmer Lambda 2 spectrometer. Steady-state fluorescence spectra in solution were collected from a FluoroMax-4 spectrofluorometer (Piscataway, NJ, USA). Emission spectra were spectrally corrected and fluorescence quantum yields include the correction due to solvent refractive index and were determined relative to quinine bisulfate in 0.05 molar sulfuric acid (Φ = 0.52) [32].

The fluorescence lifetimes were measured using a Nano LED emitting at 372 nm as an excitation source with a nano led controller module, Fluorohub from IBH, operating at 1MHz. The detection was based on an R928P type photomultiplier from Hamamatsu with high sensitivity photon-counting mode. The decays were fitted with the iterative reconvolution method on the basis of the Marquardt/Levenberg algorithm [33]. Such a reconvolution technique allows for an overall time resolution down to 0.2 ns. The quality of the exponential fits was checked using the reduced χ2 (≤ 1.2).

The two-photon absorption (2PA) measurements were performed with a femtosecond mode-locked laser pulse using a Ti: Sapphire laser (Spectra-Physics, Mai Tai: pulse duration: ~100 fs; repetition rate: 80 MHz; wavelength range: 690–1040 nm). A relative two-photon excited fluorescence method [34] was employed to measure the two-photon absorption cross-sections, δ. The measurements of 2PA cross-sections were performed relative to reference molecules (r) such as fluorescein [35] in water at pH = 11. The value of δ for a sample (s) is given by:

$$\delta_{S}=\frac{S_{S}\Phi_{r}\eta_{r}c_{r}}{S_{r}\Phi_{S}\eta_{S}c_{S}}\cdot \delta_{r}$$

where S is the detected two-photon excited fluorescence integral area, c the concentration of the chromophores, and Φ is the fluorescence quantum yield of the chromophores. η is the collection efficiency of the experimental set-up and accounts for the wavelength dependence of the detectors and optics as well as the difference in refractive indices between the solvents in which the reference and sample compounds are dissolved. The measurements were conducted in a regime where the fluorescence signal showed a quadratic dependence on the intensity of the excitation beam, as expected for two-photon induced emission. For the calibration of the two-photon absorption spectra, the two-photon excited fluorescence signal of each compound was recorded at the same excitation wavelength as that used for standards. The laser intensity was in the range of 0.2−2 × 10^9^ W/cm^2^. The experimental error on the reported cross-section was 15%.

For SHG imaging, the sample was illuminated wide field under normal incidence with femtosecond pulsed infrared (IR) laser light at 1030 nm (Pharos, Light Conversion, Vilnius, Lithuania). The intensity and polarization of the incident IR light was varied by a combination of a zero-order half-wave plate for 1030 nm mounted in a computer-controlled rotation stage (Thorlabs, PRM-Z8, Newton, NJ, USA) and a Glan-Taylor polarizer selecting for S-polarized light. The sample was irradiated by a long focal length lens (f = 5 cm) which was focused above the sample, so that the incident fundamental light could be considered to a good approximation as a collimated beam and electric field components along the propagation direction (Z) can be neglected. Behind the sample, a 20x objective (Nikon, Tokyo, Japan, CFI Plan Fluor 20X CH) collects the light. In the infinity path an IR filter rejects the laser light and a filter wheel selects the transmitted wavelength for SHG (Bandpass, 515nm, Edmund Optics #65–153), MPF (Longpass, Cut-off wavelength 525nm, Edmund Optics #84–744, York, UK) or bright field (no filter). A 20 cm tube lens (Mitutoyo, Kawasaki, Japan) then images the light onto the slit of an imaging spectrometer (Andor, Kymera 328i, Oxford Instruments, Abingdon, UK), coupled to an I-CCD camera (Andor, iStar 340, Oxford Instruments, Abingdon, UK). By switching between a mirror and a grating (150 L/mm groove density; blaze = 500 NM), the spectrometer can be used for imaging and spectroscopy, respectively. The latter option requires closing the slit of the spectrometer to ensure adequate spectral resolution. For bright-field imaging, an LED source mounted above the sample was used. This LED source could be polarization selected by a broadband polarizing sheet, positioned perpendicular to a rotatable broadband polarizer in the detection path. In this manner, polarized optical microscopy images could be recorded. SHG images were obtained using the same laser line coupled to a commercial epi-SHG microscope in scanning mode (Thorlabs MM101, Newton, NJ, USA), with a 20× objective (Nikon (Tokyo, Japan), CFI Plan Fluor 20× CH).

## Figures and Tables

**Table 1:** Thermal behaviors of derivatives **3–13**.

Compounds	Grafting	Transition Temperatures T(°C) [ΔH, kJ/mol]
**3**	**(*S*)-3,4,5-citro-C8(2)**	**Heating** → Cr 156.7 [37.01] Iso **Cooling** → Iso 126.5 [−37.68] Cr
**4**	**(*S*)-3,4-citro-C8(2)**	**Heating** → Cr_1_ 61.8 [4.75] Cr_2_ 190.8 [32.23] Iso **Cooling** → Iso 180.2 [−0.31] Cr_3_ 172.6 [−32.49] Cr_2_ 14.8 [−4.17] Cr_1_
**5**	**(*S*)-4-citro-C8(2)**	**Heating** → Cr_1_ 150.9 [0.25] Cr_2_ 190.6 [0.57] Cr_3_ 250 Decomp **cooling** → Cr_3_ 185 [−1.12] Cr_1_
**6**	**(*R*)-3,4-lact-C8**	**Heating** → Cr 168.3 [26.6] Iso **Cooling** → Iso 158.2 [−26.22] Cr
**7**	**(*R*)-4-lact-C8**	**Heating** → LC 261.2 [47.7] Iso **Cooling** → Iso 245.9 [−45.11] LC
**8**	**(*S*)-4-diol-C8**	**Heating** → Cr_1_ 161.1 [−15.6] Cr_2_ 201.4 [36.24] Cr_3_ + Iso 250.1 [4.91] Iso **Cooling** → Iso 235.4 [−6.79] Cr_3_ 191.2 [−16.62] Cr_2_ 114.2 [−0.97] Cr_1_
**9**	**(*S*)-4-diol-C12**	**Heating** → Cr_1_ 42.9 [4.4] Cr_2_ 192 [47.08] Iso **Cooling** → Iso 192.9 [−1.56] Cr_3_ 179.7 [−43.71] Cr_2_ 39.3 [−6.79] Cr_1_
**10**	**3,4,5-C8**	**Heating** → Cr_1_ 73.1 [5.3] Cr_2_ 184 [50.69] Iso **Cooling** → Iso 157.3 [−47.39] Cr_2_ 36.8 [−3.61] Cr_1_
**11**	**3,4,5-C12**	**Heating** → Cr 139.9 [42.02] Iso **Cooling** → Iso 122.5 [−45.62] Cr
**12**	**3,4-C12**	**Heating** → Cr_1_ 93.9 [2.05] Cr_2_ 123 [1.04] Cr_3_ 196.6 [32.6] Iso **Cooling** → Iso 183.5 [−31.58] Cr_2_ 81.7 [−2.86] Cr_1_
**13**	**3,4-C16**	**Heating** → Cr_1_ 92.8 [5.85] LC143.3 [1.49] LC’ 183.1 [28.6] Iso **Cooling** → Iso 173.5 [−28.14] LC 82.7 [−8.6] Cr_1_
**PMC-3,4,5-C16** [12]	**3,4,5-C16**	**Heating** → Cr_1_ 128.6 [53.0] Sm 136.0 [-] Iso **Cooling** → Iso 135.4 [−0.32] Sm 82.7 [−57.81] Cr_1_

Cr = Crystal, Sm = smectic phase, Iso = isotropic phase, Decomp = decomposition, LC = liquid crystalline phase.

**Table 2:** Table of indexed diffraction peaks of the liquid crystalline phase at 80 °C for compound **7**.

2θ (°)	d(Å) Measured	d(Å) Calculated (Col_o_ Cell)	Δ (d)	Indexation	Cell Parameters
1.18	74.77	75.92	1.15	10	a = 75.92 Å
1.86	47.44	47.65	0.21	01	b = 47.65 Å
2.39	36.92	36.96	0.04	11	β = 100 °
3.15	28.01	27.04	−0.97	21	Cell area = 3563 Å^2^
3.67	24.05	24.92	0.87	30	V_molecule_ = 1227 Å^3^
5.59	15.79	15.77	−0.02	32	V_cell_ = 29,448 Å^3^
21.68 (halo)	4.09	/	/	/	M = 738.93 g·mol^−1^

**Table 3:** Table of indexed diffraction peaks of the liquid crystalline phase at 100 °C for compound **13**.

2θ (°)	d(Å) Measured	d(Å) Calculated (Col_r_ cell)	Δ (d)	Indexation	Cell Parameters
0.93 1.52 1.85 2.83 21.52 (halo)	94.78 58.05 47.7 31.18 4.12	94.88 58.05 47.44 31.62 /	0.1 0.0 −0.26 0.44 /	20 11 40 60 /	a = 189.76 Å b = 60.97 Å β = 90 ° Cell area = 11.570 Å^2^ V_molecule_ = 1717 Å^3^ V_cell_ = 54,944 Å^3^ M = 1033.49 g·mol^−1^

**Table 4:** 1PA optical properties of the series of molecules in THF.

	Compound	λ_abs_ (ε)/nm (10^3^ M^−1^·cm^−1^)	λ_fluo_ (nm)	Φ_fluo_	E_00_ ^(a)^/eV	τ_fluo_^(b)/^ns	k_r_ ^(c)^ /10^9^ s^−1^	k_nr_ ^(c)^ /10^9^ s^−1^	k_nr_/k_r_
SERIE 1	**5 (*S*)-4-citro-C8(2)**	/	/	/	/	/	/	/	/
	**7 (*R*)-4-lact-C8**	378 (104.8)	457	0.82	3.00	1.43	0.57	0.13	0.23
	**8 (*S*)-4-diol-C8**	378 (100.9)	456	0.78	3.00	1.38	0.57	0.16	0.28
	**9 (*S*)-4-diol-C12**	378 (105.6)	458	0.78	3.00	1.41	0.55	0.16	0.28
SERIE 2	**6 (*R*)-3,4-lact-C8**	379 (109.1)	497	0.80	2.88	2.09	0.38	0.10	0.25
	**4 (*S*)-3,4-citro-C8(2)**	379 (101.4)	498	0.82	2.88	2.13	0.38	0.08	0.22
	**12 3,4-C12**	379 (117.1)	499	0.76	2.88	2.14	0.36	0.11	0.31
	**13 3,4-C16**	/	/	/	/	/	/	/	/
SERIE 3	**3 (*S*)-3,4,5-citro-C8(2)**	377 (79.4)	524	0.46	2.83	1.56	0.29	0.35	1.17
	**10 3,4,5-C8**	378 (88.2)	532	0.35	2.81	1.53	0.23	0.42	1.82
	**11 3,4,5-C12**	378 (91.5)	533	0.35	2.81	1.49	0.23	0.44	1.86
	**PMC-3,4,5-C16 3,4,5-C16** [12]	378 (93.3)	532	0.39	2.81	1.48	0.26	0.41	1.58

^(a)^ E_00_ ≈ ½ hc(ν_abs_ + ν_fluo_). ^(b)^ Well-fitted as a mono-exponential decay ^(c)^ k_r_ = Φ_fluo_/τ_fluo_ and k_nr_ = (1 − Φ_fluo_)/τ_fluo._

**Table 5:** 2PA spectroscopic data of the molecules recorded in THF (uncertainties on the values of δ is 15%).

		High-Energy Band		Low-Energy Band		
	Compound	δ_2PA_/GM	λ_2PA_/nm	δ_2PA_/GM	λ_2PA_/nm	Ratio δ_2PA_
SERIE 1	**5 (*S*)-4-citro-C8(2)**	/	/	/	/	/
	**7 (*R*)-4-lact-C8**	164	700	99	790	1.66
	**8 (*S*)-4-diol-C8**	123	700	77	790	1.60
	**9 (*S*)-4-diol-C12**	157	700	102	790	1.54
SERIE 2	**6 (*R*)-3,4-lact-C8**	143	715	110	805	1.30
	**4 (*S*)-3,4-citro-C8(2)**	175	715	136	805	1.29
	**12 3,4-C12**	205	700	156	805	1.31
	**13 3,4-C16**	/	/	/	/	/
SERIE 3	**3 (*S*)-3,4,5-citro-C8(2)**	238	715	150	775	1.59
	**10 3,4,5-C8**	238	700	136	805	1.75
	**11 3,4,5-C12**	118	700	73	790	1.62
	**PMC-3,4,5-C16** [12]	121	715	76	790	1.59

## Data Availability

The data presented in this study are available on request from the corresponding authors.

## Supplementary Materials

The following supporting information can be downloaded at: https://www.mdpi.com/article/10.3390/molecules30214322/s1, Figure S1: POM images, DSC and SAXS of compound **3 ((*S*)-3,4,5-citro-C8(2))**; Figure S2: POM images, DSC and SAXS of compound **4 ((*S*)-3,4-citro-C8(2))**; Figure S3: POM images, DSC and SAXS of the compound **5 ((*S*)-4-citro-C8(2))**; Figure S4: POM images, DSC and SAXS of compound **6 ((R)-3,4-lact-C8)**; Figure S5: POM images, DSC and SAXS of compound **8 ((*S*)-4-diol-C8)**; Figure S6: POM images, DSC and SAXS of compound **9 ((*S*)-4-diol-C12)**; Figure S7: POM images, DSC and SAXS of compound **10 (3,4,5-C8)**; Figure S8: POM images, DSC and SAXS of compound **11 (3,4,5-C12)**; Figure S9: POM images of compound **12 (3,4-C12)**. Supplementary Materials with full synthetic details and additional POM, DSC, and SAXS analyses are available online or from the author. References [17,18,19,20,21,23,24,25,26,36] are cited in the Supplementary Materials.
