# Supplementary material for: From Chains to Chromophores: Tailored Thermal and Linear/Nonlinear Optical Features of Asymmetric Pyrimidine—Coumarin Systems
Source: Molecules. 2025 Nov 6;30(21):4322. doi: 10.3390/molecules30214322 (PMC12608648; doi:10.3390/molecules30214322)
Supplement: Supplementary file 1 [file molecules-30-04322-s001.zip › molecules-3947022-supplementary.pdf]

Supporting information

Syntheses.

Synthesis of compound 1:

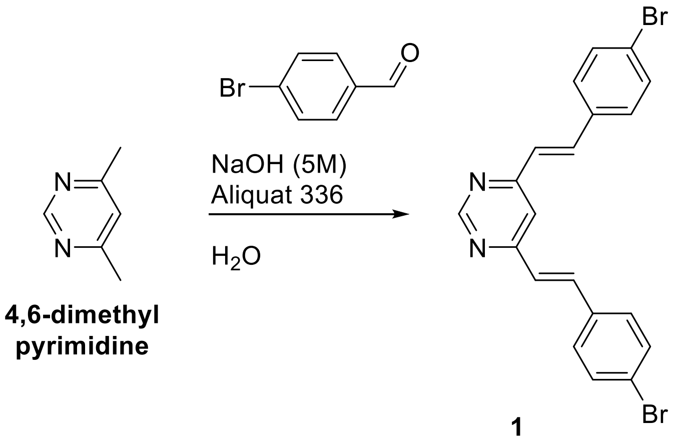

1

4,6-dimethylpyridine (23.8 mmol, 2.57 g) and 4-bromobenzaldehyde (71.4 mmol, 13.21 g) were added in an aqueous solution of NaOH (5M) (90ml). Aliquat 336 (10% mol, 0.962 g) was added. The solution was refluxed for 24h. After cooling the solution, the precipitate was filtrated and washed consecutively with water, methanol, petroleum ether and dichloromethane. The white solid was dried under vaccum affording the desired product without purification. Compound **1** was obtained as white powder (5.62 g, 53%).

^{1}H NMR (300 MHz, THF) δ 9.00 (d, J = 1.2 Hz, 1H, $CH_{aromatic}$), 7.95 (d, J = 15.9 Hz, 2H, $CH_{ethylenic}$), 7.58 (d, J = 3.4 Hz, 8H, $CH_{aromatic}$), 7.44 (d, J = 1.3 Hz, 1H, $CH_{aromatic}$), 7.22 (d, J = 15.9 Hz, 2H, $CH_{ethylenic}$).

^{13}C NMR (75 MHz, THF) δ 163.73 (C_{quat}), 159.68 (CH), 136.44 (C_{quat}), 136.28 (C_{quat}), 133.00 (CH), 130.30 (CH), 128.01 (CH), 123.96 (CH), 117.77 (CH).

Synthesis of the coumarin fragment (7-ethynyl-2H-chromen-2-one)

7-hydroxycoumarin (6.2 mmol, 1 g) was dissolved in dry pyridine (20 mL). The solution was cooled in an ice bath and then trifluoromethanesulfonic anhydride (6.2 mmol, 1 mL) was added. The mixture was stirred for 4h at 0 °C. Diethyl ether (100 mL) was added to the solution and the precipitate was filtered and discarded. An aqueous solution of chlorhydric acid (1M, 5 mL) was added to the filtrate and after stirring the solution for 1h, organic solvents were removed under reduced pressure. An aqueous solution of chlorhydric acid (1M, 75 mL) was added and the product was extracted with DCM, washed with water, dried over $MgSO_4$ and concentrated under reduced pressure. The product was purified by silica gel chromatography (PE – PE:DCM (5:5) gradient in 20 minutes). The compound **2-oxo-2H-chromen-7-yl trifluoromethanesulfonate** was obtained as a white solid (1.55 g, 88 %).
^{1}H NMR (300 MHz, $CDCl_3$) δ 7.74 (dd, J = 9.6, 0.7 Hz, 1H, $CH_{ethylenic}$), 7.60 (d, J = 8.5 Hz, 1H, $CH_{aromatic}$), 7.26 (d, J = 2.4 Hz, 1H, $CH_{aromatic}$), 7.21 (dd, J = 8.5, 2.4 Hz, 1H, $CH_{aromatic}$), 6.49 (d, J = 9.6 Hz, 1H, $CH_{ethylenic}$).
^{13}C NMR (75 MHz, $CDCl_3$) δ 159.21 (C=O), 154.51 (C_{quat}), 150.75 (C_{quat}), 142.27 (CH), 129.65 (CH), 120.76 (C_{quat}), 118.87 (C_{quat}), 117.58 (CH), 116.50 (C_{quat}), 110.29 (CH).

Under nitrogen in a Schenk, compound **2-oxo-2H-chromen-7-yl trifluoromethanesulfonate** (2.0 mmol, 0.59 g) was dissolved in dry trimethylamine (9 mL). $Pd(PPh_3)_2Cl_2$ (0.2 mmol, 0.14 g) and CuI (0.2 mmol,

0.039 g) were added and the solution was degassed during 30 minutes. Trimethylsilylacetylene (5.8 mmol, 0.72 mL) was syringed and the mixture was stirred at 90 °C for 24 h. After cooling the solution, the mixture was diluted with DCM and filtered through celite column. Solvents were removed by reduced pressure and the product was extracted with DCM, washed with water, dried over $MgSO_4$ and concentrated under reduced pressure. The product was purified by silica gel chromatography (PE – PE:AcOEt (8:2) gradient in 20 minutes). The compound **7-((trimethylsilyl)ethynyl)-2H-chromen-2-one** was obtained as a yellow pale solid (0.40 g, 82 %).
^{1}H NMR (300 MHz, $CDCl_3$) δ 7.67 (d, *J* = 9.5 Hz, 1H, $CH_{ethylenic}$), 7.43 – 7.37 (m, 2H, $CH_{aromatic}$), 7.34 (dd, *J* = 8.1, 1.3 Hz, 1H, $CH_{aromatic}$), 6.42 (d, *J* = 9.5 Hz, 1H, $CH_{ethylenic}$), 0.27 (s, 9H, CH_3-TMS).
^{13}C NMR (75 MHz, $CDCl_3$) δ 160.39 (C=O), 153.79 (C_{quat}), 142.80 (CH), 128.08 (CH), 127.72 (CH), 126.89 (C_{quat}), 119.99 (CH), 118.94 (C_{quat}), 117.09 (CH), 103.38 (C_{quat}), 98.68 (C_{quat}), -0.12 (CH_3-TMS).

Compound **7-((trimethylsilyl)ethynyl)-2H-chromen-2-one** (3.5 mmol, 0.854 g) and KF (35 mmol, 2.034 g) were dissolved in a mixed of THF/MeOH (1/1) (12mL). The mixture was stirred overnight at room temperature. Solvents were removed under reduced pressure. The product obtained was dissolved in DCM, washed with water, dried over $MgSO_4$ and concentrated under reduced pressure. The product was purified by silica gel chromatography (100% DCM). The compound **(7-ethynyl-2H-chromen-2-one)** was obtained as a white solid (0.374 g, 63 %).
^{1}H NMR (300 MHz, $CDCl_3$) δ 7.68 (dd, *J* = 9.6, 0.7 Hz, 1H, $CH_{ethylenic}$), 7.45 – 7.35 (m, 3H, $CH_{aromatic}$), 6.43 (d, *J* = 9.5 Hz, 1H, $CH_{ethylenic}$), 3.26 (s, 1H, CH_{alkyne}).
^{13}C NMR (75 MHz, $CDCl_3$) δ 160.31 (C=O), 153.85 (C_{quat}), 142.76 (CH), 128.22 (CH), 127.88 (CH), 125.89 (C_{quat}), 120.48 (CH), 119.32 ($C_{quat-alkyne}$), 117.48 (CH_{alkyne}), 82.28 (CH), 80.78 (CH).

Synthesis of compound 2:

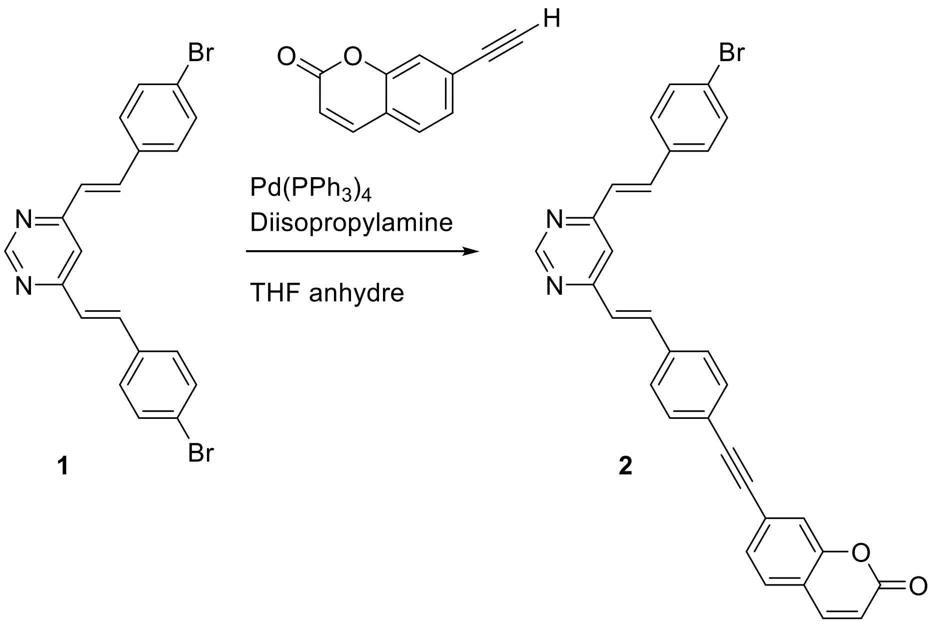

Compound **1** (1.2 mmol, 0.531 g) and **7-((trimethylsilyl)ethynyl)-2H-chromen-2-one** (1.2 mmol, 0.208 g) (full synthetic details given at the end of this syntheses part) were dissolved in dry THF (20 ml) and diisopropylamine (4 ml). The solution was degassed during 30 minutes. $Pd(PPh_3)_4$ (0,12 mmol, 0.139 g) is added and the mixture was heated at 60°C during 48h. The precipitate obtained was filtrated under reduce pressure and washed with THF. Because of solubility troubles, compound **26** is obtained without purification as a bright yellow powder (0.593, 93%). The compound was found insoluble in all organics deutered solvents.

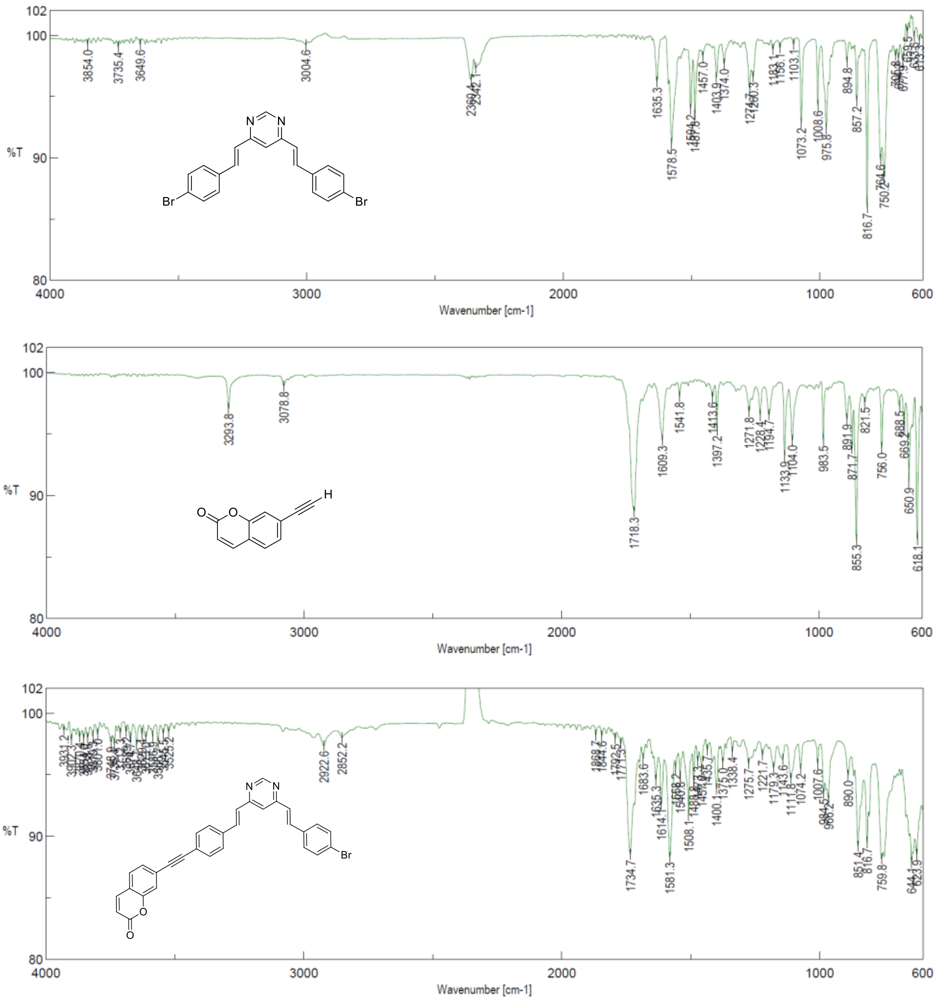

IR spectra of compounds **1**, **7-((trimethylsilyl)ethynyl)-2H-chromen-2-one** and **2** (FT-IR spectra were recorded using a JASCO FT/IR-4600 spectrometer equipped with an ATR apparatus).

Synthesis of the pro-mesogens:

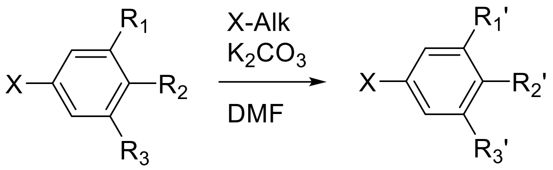

Alkylation
23-88%

14-25

Position 3,4,5: (R_1 = OH, R_2 = OH, R_3 = OH)
Position 3,4: (R_1 = OH, R_2 = OH, R_3 = H)
Position 4: (R_1 = H, R_2 = OH, R_3 = H)

Position 3,4,5: R_1' = O-Alk, R_2' = O-Alk, R_3' = O-Alk, X = Br
Position 3,4: R_1' = O-Alk, R_2' = O-Alk, R_3' = H, X = Br
Position 4: R_1' = H, R_2' = O-Alk, R_3' = H, X = I

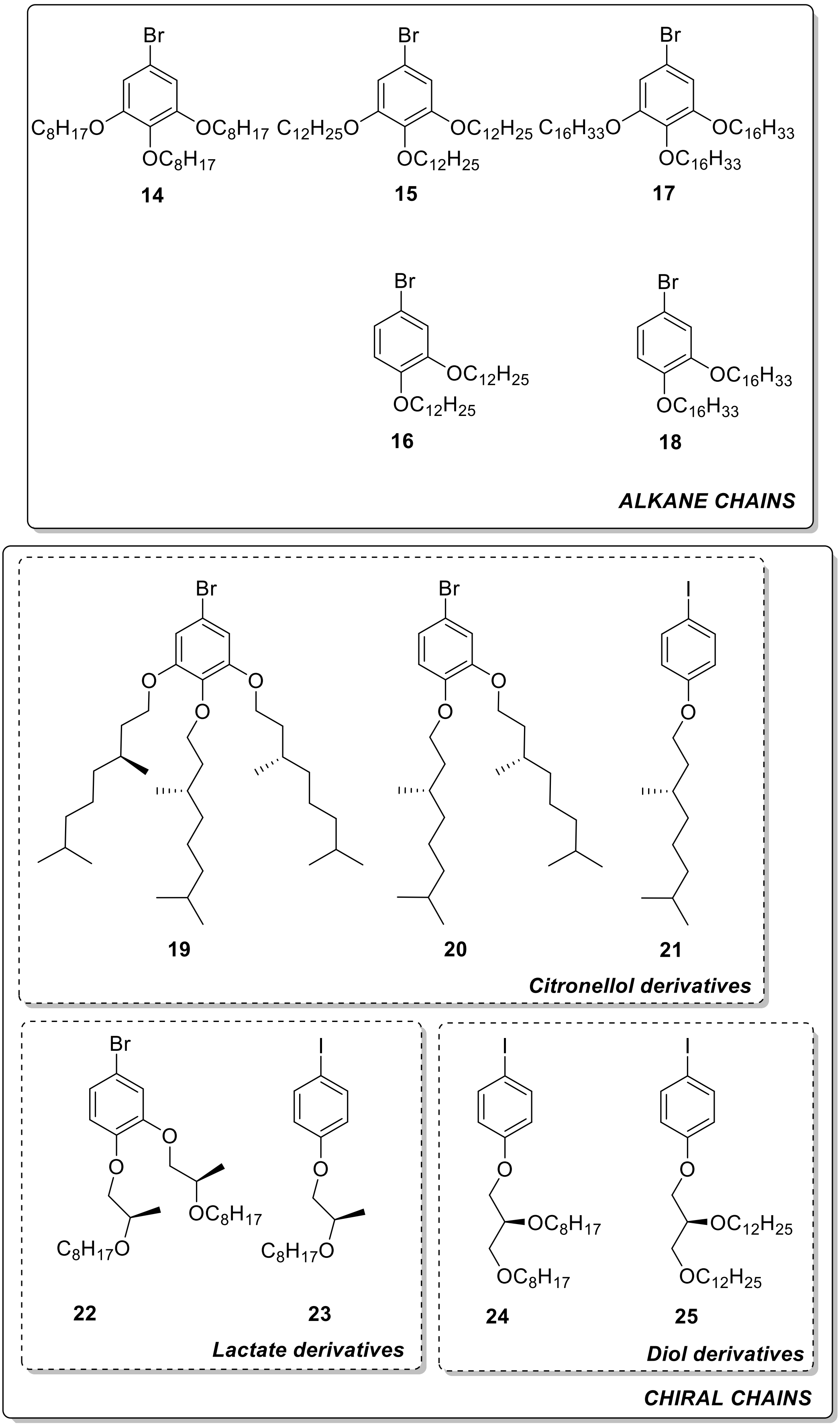
Br
$C_8H_{17}O$
OC_8H_{17}
OC_8H_{17}
14
Br
$C_{12}H_{25}O$
$OC_{12}H_{25}$
$OC_{12}H_{25}$
15
Br
$C_{16}H_{33}O$
$OC_{16}H_{33}$
$OC_{16}H_{33}$
17
Br
$OC_{12}H_{25}$
$OC_{12}H_{25}$
16
Br
$OC_{16}H_{33}$
$OC_{16}H_{33}$
18
ALKANE CHAINS
Br
O
O
O
19
Br
O
O
20
I
O
21
Citronellol derivatives
Br
O
O
OC_8H_{17}
$C_8H_{17}O$
22
I
O
$C_8H_{17}O$
23
Lactate derivatives
I
O
OC_8H_{17}
OC_8H_{17}
24
I
O
$OC_{12}H_{25}$
$OC_{12}H_{25}$
25
Diol derivatives
CHIRAL CHAINS

General procedure A:

Compounds **14-25** were prepared according General Procedure A. Alcohol derivatives (1 eq.) was dissolved in DMF (30 mL/g(OH)), K_2CO_3 (3 eq./OH) was added under nitrogen and the alkane chain (2 eq./OH) was slowly added. The reaction mixture was stirred for 72 h at 90 °C. After cooling to room temperature, DMF was removed under reduced pressure. The product was solubilized in DCM, washed with water, dried over $MgSO_4$ and concentrated under reduced pressure. The product was purified by silica gel chromatography.

***Synthesis of compound 14**:*[1]
Compound **14** was prepared from 5-bromobenzene-1,2,3-triol and 1-bromooctane. The product was purified by silica gel chromatography (PE:DCM (5:5)) and recrystallized by slow evaporation (CH_2Cl_2/MeOH) to give **14** as white powder (54 %).
^{1}H NMR (300 MHz, $CDCl_3$) δ 6.67 (s, 2H, $CH_{aromatic}$), 3.95 – 3.90 (m, 6H, OCH_2), 1.86 – 1.60 (m, 6H, CH_2), 1.52 – 1.41 (m, 6H, CH_2), 1.30 (broad s, 24H, CH_2), 1.02 – 0.79 (m, 9H, CH_3). The analytical results are in good agreement with the data published in the literature. [1]

***Synthesis of compound 15**:*[1]
Compound **15** was prepared from 5-bromobenzene-1,2,3-triol and 1-bromododecane. The product was purified by silica gel chromatography (PE:DCM (5:5)) and recrystallized by slow evaporation (CH_2Cl_2/MeOH) to give **15** as white powder (23 %).
^{1}H NMR (300 MHz, $CDCl_3$) δ 6.68 (s, 2H, $CH_{aromatic}$), 3.92 (q, *J* = 6.6 Hz, 6H, OCH_2), 1.86 – 1.64 (m, 6H, CH_2), 1.51 – 1.41 (m, 6H, CH_2), 1.27 (broad s, 48H, CH_2), 0.93 – 0.83 (m, 9H, CH_3). The analytical results are in good agreement with the data published in the literature. [1]

Synthesis of compound 16:[2]
Compound **28** was prepared from 4-bromobenzene-1,2-diol and 1-bromododecane. The product was purified by silica gel chromatography (PE:DCM (5:5)) and recrystallized by slow evaporation (CH_2Cl_2/MeOH) to give **16** as white powder (65 %).
^{1}H NMR (300 MHz, $CDCl_3$) δ 7.06 – 6.90 (m, 2H, $CH_{aromatic}$), 6.78 – 6.69 (m, 1H, $CH_{aromatic}$), 3.96 (t, *J* = 6.6 Hz, 2H, OCH_2), 3.95 (t, *J* = 6.6 Hz, 2H, OCH_2), 1.88 – 1.71 (m, 4H, CH_2), 1.49 – 1.40 (s, 4H, CH_2), 1.27 (broad s, 32H, CH_2), 0.94 – 0.80 (m, 6H, CH_3). The analytical results are in good agreement with the data published in the literature. [2]

Synthesis of compound 17:[3]
Compound **17** was prepared from 5-bromobenzene-1,2,3-triol and *1*-bromohexadecane. The product was purified by silica gel chromatography (PE:DCM (5:5)) and recrystallized by slow evaporation (CH_2Cl_2/MeOH) to give **17** as white powder (64 %).
^{1}H NMR (300 MHz, $CDCl_3$) δ 6.67 (s, 2H, $CH_{aromatic}$), 3.92 (q, *J* = 6.6 Hz, 6H, OCH_2), 1.86 – 1.64 (m, 6H, CH_2), 1.50 – 1.40 (m, 6H, CH_2), 1.26 (s, 72H, CH_2), 0.93 – 0.83 (m, 9H, CH_3). The analytical results are in good agreement with the data published in the literature. [3]

Synthesis of compound 18: [4]

Compound **18** was prepared from 4-bromobenzene-1,2-diol and 1-bromohexadecane. The product was purified by silica gel chromatography (PE:DCM (5:5)) and recrystallized by slow evaporation (CH_2Cl_2/MeOH) to give **18** as white powder (88 %).

^{1}H NMR (300 MHz, $CDCl_3$) δ 7.03 – 6.93 (m, 2H, $CH_{aromatic}$), 6.81 – 6.67 (m, 1H, $CH_{aromatic}$), 3.96 (t, *J* = 6.6 Hz, 2H, OCH_2), 3.95 (t, *J* = 6.6 Hz, 2H, OCH_2), 1.84 – 1.74 (m, 4H, CH_2), 1.50 – 1.40 (m, 4H, CH_2), 1.26 (s, 48H, CH_2), 0.93 – 0.82 (m, 6H, CH_3). The analytical results are in good agreement with the data published in the literature. [4]

Synthesis of (S)-1-bromo-3,7-dimethyloctane: [5]

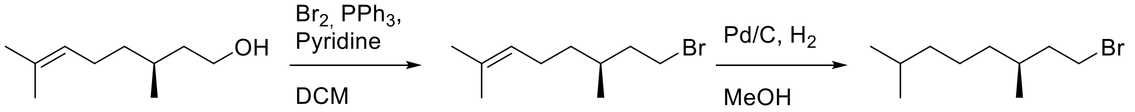

(S)-3,7-dimethyloct-6-en-1-ol ***(S)-8-bromo-2,6-dimethyloct-2-ene*** ***(S)-1-bromo-3,7-dimethyloctane***

(S)-8-bromo-2,6-dimethyloct-2-ene:

PPh_3 (60 mmol, 15.72 g) was dissolved in DCM (50 mL) and then the solution was cooled to 0 °C. Br_2 (55 mmol, 2.82 mL) was syringed drop by drop and the solution was stirred at 0 °C for 1 h. *(S)*-3,7-dimethyloct-6-en-1-ol (50 mmol, 7.8 g) was dissolved in DCM (25 mL) and added dropwise to the solution at 0 °C. Pyridine (55 mmol, 4.5 mL) was also added and the mixture was stirred at room temperature for 24 h. The mixture was filtered and the solvent was removed under reduced pressure. The product was dissolved in PE and filtered over celite. The filtrate was concentrated under reduced pressure and purified by alumina gel chromatography (PE) to give *(S)*-8-bromo-2,6-dimethyloct-2-ene as a colorless oil (9.62 g, 88 %).

^{1}H NMR (300 MHz, $CDCl_3$) δ 5.09 (s, 1H, $CH_{ethylenic}$), 3.50 – 3.36 (m, 2H, CH_2-Br), 2.08 – 1.83 (m, 3H, CH2, C*CH), 1.83 – 1.57 (m, 8H, 2 CH_3, CH_2), 1.45 – 1.07 (m, 2H, CH_2), 0.91 (d, *J* = 6.5 Hz, 3H, C*CH_3). The analytical results are in good agreement with the data published in the literature. [5]

(S)-1-bromo-3,7-dimethyloctane:

(S)-8-bromo-2,6-dimethyloct-2-ene (44.5 mmol, 9.62 g) was dissolved in MeOH (50 mL). Palladium on carbon (9.5 mmol, 1 g) was added to the solution and then the solution was degassed with H_2 during 30 minutes. Then the solution was stirred under H_2 atmosphere for 72 h. The mixture was filtered over celite. The filtrate was concentrated under reduced pressure and purified by silica gel chromatography (PE) to give *(S)*-1-bromo-3,7-dimethyloctane as a colorless oil (9.84 g, 99 %).

^{1}H NMR (300 MHz, $CDCl_3$) δ 3.53 – 3.33 (m, 2H, BrCH_2), 1.97 – 1.78 (m, 1H, C*CH), 1.75 – 1.44 (m, 2H, CH_2), 1.37 – 1.07 (m, 7H, 3 CH_2, CH), 0.88 (t, *J* = 6.1 Hz, 9H, 3 CH_3). The analytical results are in good agreement with the data published in the literature. [5]

Synthesis of compound 19:

Compound **19** was prepared from 5-bromobenzene-1,2,3-triol and *(S)*-1-bromo-3,7-dimethyloctane. The product was purified by silica gel chromatography (PE:DCM (5:5)) to give **19** as a colorless oil (64 %).

^{1}H NMR (300 MHz, $CDCl_3$) δ 6.70 (s, 2H, $CH_{aromatic}$), 4.05 – 3.87 (m, 6H, OCH_2), 1.93 – 1.77 (m, 3H, C*-CH), 1.75 – 1.68 (m, 3H, CH), 1.64 – 1.45 (m, 6H, CH_2), 1.42 – 1.08 (m, 18H, CH_2), 0.95 (d, *J* = 6.6 Hz, 6H, C*-CH_3), 0.94 (d, *J* = 6.6 Hz, 3H, C*-CH_3), 0.89 (d, *J* = 6.6 Hz, 18H, CH_3).

^{13}C NMR (75 MHz, $CDCl_3$) δ 153.96 (C_{quat}), 137.65 (C_{quat}), 115.70 (C_{quat}), 110.30 (CH), 71.73 (OCH2), 67.71 (2 OCH2), 39.50 (CH_2), 39.40 (2 CH_2), 37.64 (CH_2), 37.45 (2 CH_2), 37.44 (3 CH_2), 36.42 (2 C*CH), 29.92

(C*CH), 29.79 (3 CH), 28.10 (2 C*CH_2), 24.85 (2 C*CH_2), 24.83 (C*CH_2), 22.82 (3 CH_3), 22.72 (2 C*CH_3), 19.70 (C*CH_3), 19.68 (3 CH_3).

Synthesis of compound 20:
Compound **20** was prepared from 4-bromobenzene-1,2-diol and *(S)*-1-bromo-3,7-dimethyloctane. The product was purified by silica gel chromatography (PE:DCM (5:5)) to give **20** as a colorless oil (42 %).
^{1}H NMR (300 MHz, $CDCl_3$) δ 7.03 – 6.94 (m, 2H, $CH_{aromatic}$), 6.78 – 6.69 (m, 1H, $CH_{aromatic}$), 4.05 – 3.92 (m, 4H, OCH_2), 1.91 – 1.77 (m, 2H, C*-CH), 1.74 – 1.44 (m, 6H, C*-CH, CH_2), 1.41 – 1.09 (m, 12H, CH_2), 0.95 (d, *J* = 2.7 Hz, 3H, C*-CH_3), 0.93 (d, *J* = 2.7 Hz, 3H, C*-CH_3), 0.88 (d, *J* = 1.2 Hz, 6H, CH_3), 0.86 (d, *J* = 1.2 Hz, 6H, CH_3).
^{13}C NMR (75 MHz, $CDCl_3$) δ 150.23 (C_{quat}), 148.56 (C_{quat}), 123.57 (CH), 117.07 (CH), 115.25 (CH), 112.94 (C_{quat}), 67.99 (OCH_2), 67.86 (OCH_2), 39.39 (2 CH_2), 37.47 (CH_2), 37.45 (CH_2), 36.34 (CH_2), 36.26 (CH_2), 30.03 (2 C*CH), 28.10 (2 CH), 24.84 (2 C*CH_2), 22.83 (2 CH_3), 22.73 (2 C*CH_3), 19.80 (2 CH_3).

Synthesis of compound 21:
Compound **21** was prepared from 4-iodophenol and *(S)*-1-bromo-3,7-dimethyloctane. The product was purified by silica gel chromatography (PE:DCM (5:5)) to give **21** as a colorless oil (87 %).
^{1}H NMR (300 MHz, $CDCl_3$) δ 7.56 (d, *J* = 9.1 Hz, 2H, $CH_{aromatic}$), 6.69 (d, *J* = 9.1 Hz, 2H, $CH_{aromatic}$), 4.03 – 3.91 (m, 2H, OCH_2), 1.90 – 1.79 (m, 1H, CH), 1.76 – 1.67 (m, 1H, C*-CH), 1.66 – 1.52 (m, 2H, CH_2), 1.42 – 1.14 (m, 6H, CH_2), 0.98 (d, *J* = 6.4 Hz, 3H, C*CH_3), 0.93 (d, *J* = 6.6 Hz, 6H, CH_3).
^{13}C NMR (75 MHz, $CDCl_3$) δ 159.08 (C_{quat}), 138.21 (2 CH), 117.04 (2 CH), 82.52 (C_{quat}), 66.50 (OCH_2), 39.34 (CH_2), 37.38 (CH_2), 36.19 (CH_2), 29.94 (C*CH), 28.06 (CH), 24.76 (C*CH_2), 22.85 (CH_3), 22.75 (C*CH_3), 19.79 (CH_3).

Synthesis of ((R)-1-((1-iodopropan-2-yl)oxy)octane):[6]

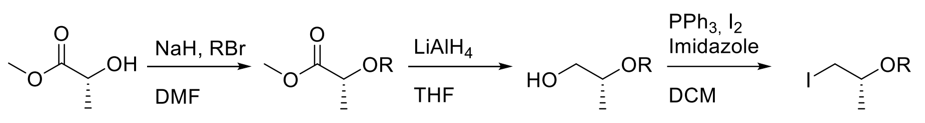

methyl (R)-2-hydroxypropanoate | ***R = C_8H_{17} (methyl (R)-2-(octyloxy)propanoate)*** | ***R = C_8H_{17} ((R)-2-(octyloxy)propan-1-ol)*** | ***R = C_8H_{17} ((R)-1-((1-iodopropan-2-yl)oxy)octane)***

Methyl (R)-2-(octyloxy)propanoate:
Under nitrogen, methyl *(R)*-2-hydroxypropanoate (38.4 mmol, 4 g) and bromooctane (57.6 mmol, 10 mL) were dissolved in dry DMF (50 mL). The solution was cooled with an ice bath to 0 °C and NaH 60 % dispersed in oil (64 mmol, 1.536 g) was slowly added. The mixture was stirring overnight at room temperature. Water was added to quench the reaction and the solvent was removed under reduced pressure. The product obtained was dissolved in DCM, washed with water, dried over $MgSO_4$ and concentrated under reduced pressure. The product was purified by silica gel chromatography (PE – (PE:DCM (5:5) gradient in 30 minutes) (the product is not UV visible so ethanol/phosphomolibdic acid solution is used to reveal TLC). Compound methyl *(R)*-2-(octyloxy)propanoate was obtained as a colorless liquid (1.86 g, 22 %).
^{1}H NMR (300 MHz, $CDCl_3$)**)** δ 3.95 (q, J = 6.8 Hz, 1H, C*H), 3.73 (s, 3H, OCH_3), 3.57 – 3.50 (m, 1H, OCH_2), 3.38 – 3.31 (m, 1H, OCH_2), 1.65 – 1.53 (m, 2H, CH_2), 1.39 (d, J = 6.9 Hz, 3H, C*CH_3), 1.36 – 1.23 (m, 10H, CH_2), 0.92 – 0.81 (m, 3H, CH_3).
^{13}C NMR (75 MHz, $CDCl_3$) δ 174.16 (C=O), 75.10 (OCH_3), 70.64 (OCH_2), 51.95 (C*CH), 31.96 (CH_2), 30.24 (CH_2), 29.86 (CH_2), 29.52 (CH_2), 26.15 (CH_2), 22.78 (CH_2), 18.02 (C*CH_3), 14.20 (CH_3).

(R)-2-(octyloxy)propan-1-ol:

Under nitrogen, compound methyl *(R)*-2-(octyloxy)propanoate (8.6 mmol, 1.86 g) was dissolved in dry THF (20 mL). The solution was cooled with an ice bath to 0 °C and $LiAlH_4$ (1M in THF) (17.2 mmol, 17.2 mL) was syringed. The mixture was stirring overnight at room temperature. Water is added to quench the reaction and the solvent was removed under reduced pressure. The product obtained was dissolved in DCM, washed with an aqueous solution of hydrochloric acid (1M) and water, dried over $MgSO_4$ and concentrated under reduced pressure. Compounds *(R)*-2-(octyloxy)propan-1-ol was obtained without further purification as a colorless liquid (1.48 g, 91 %).

^{1}H NMR (300 MHz, $CDCl_3$) δ 3.60 – 3.33 (m, 5H, C*H, OCH_2, C*CH_2), 2.10 (s, 1H, OH), 1.62 – 1.52 (m, 2H, CH_2), 1.26 (s, 10H, CH_2), 1.10 (d, J = 6.0 Hz, 3H, C*CH_3), 0.90 – 0.85 (m, 3H, CH_3).

^{13}C NMR (75 MHz, $CDCl_3$) δ 75.86 (C*H), 69.08 (OCH_2), 66.53 (C*CH_2), 31.97 (CH_2), 30.24 (CH_2), 29.59 (CH_2), 29.40 (CH_2), 26.36 (CH_2), 22.79 (CH_2), 16.02 (C*CH_3), 14.20 (CH_3).

(R)-1-((1-iodopropan-2-yl)oxy)octane:

PPh_3 (9.48 mmol, 2.49 g) was dissolved with dry DCM (100 mL). Iodine (10.27 mmol, 2.61 g) and imidazole (11.85 mmol, 0.81 g) were added to the solution. This solution was stirred for 10 minutes at room temperature. Methyl *(R)*-2-(octyloxy)propan-1-ol (7.9 mmol, 1.48 g) was added to the suspension and the reaction mixture was stirred overnight at room temperature. The solution was washed with an aqueous solution of $Na_2S_2O_3$, dried over $MgSO_4$ and concentrated under reduced pressure. The product is diluted in PE and filtered over silica column. Compound *(R)*-1-((1-iodopropan-2-yl)oxy)octane) was obtained as a yellow pale liquid (1.55 g, 66 %).

^{1}H NMR (300 MHz, $CDCl_3$) δ 3.50 – 3.31 (m, 3H, C*CH, OCH_2), 3.25 (dd, *J* = 10.1, 4.7 Hz, 1H, C*-CH_2), 3.18 (dd, *J* = 10.1, 5.9 Hz, 1H, C*-CH_2), 1.65 – 1.54 (m, 2H, CH_2), 1.40 – 1.26 (m, 13H, 5 CH2, C*CH_3), 0.94 – 0.87 (m, 3H, CH_3).

^{13}C NMR (75 MHz, $CDCl_3$) δ 74.80 (C*H), 69.35 (OCH_2), 31.98 (CH_2), 30.14 (CH_2), 29.55 (CH_2), 29.40 (CH_2), 26.33 (CH_2), 22.79 (CH_2), 20.47 (C*CH_3), 14.22 (CH_3), 11.66 (C*CH_2).

Synthesis of compound 22:

Compound **22** was prepared from 4-bromobenzene-1,2-diol and (R)-1-((1-iodopropan-2-yl)oxy)octane. The product was purified by silica gel chromatography (PE – (PE:DCM (5:5) gradient in 30 minutes) to give **22** as a colorless oil (11 %).

^{1}H NMR (300 MHz, $CDCl_3$) δ 7.04 – 6.94 (m, 2H, $CH_{aromatic}$), 6.75 (d, *J* = 7.8 Hz, 1H, $CH_{aromatic}$), 4.02 – 3.94 (m, 2H, C*CH), 3.89 – 3.71 (m, 4H, C*CH_2), 3.62 – 3.49 (m, 4H, OCH_2), 1.61 – 1.51 (s, 4H, CH_2), 1.35 – 1.22 (m, 28H, CH_2, C*CH_3), 0.96 – 0.80 (m, 6H, CH_3).

^{13}C NMR (75 MHz, $CDCl_3$) δ 150.09 (C_{quat}), 150.06 (C_{quat}), 148.52 (C_{quat}), 148.50 (C_{quat}), 124.03 (CH), 124.00 (CH), 117.73 (CH), 117.62 (CH), 115.82 (CH), 115.69 (CH), 113.29 (CH), 113.26 (CH), 73.98 (C*CH), 73.96 (C*CH), 73.93 (C*CH), 73.91 (C*CH), 73.43 (OCH_2), 73.39 (OCH_2), 73.33 (OCH_2), 73.27 (OCH_2), 69.92 (C*CH_2), 69.91 (C*CH_2), 69.87 (C*CH_2), 69.85 (C*CH_2), 31.98 (CH_2), 30.32 (CH_2), 29.61(CH_2), 29.42 (CH_2), 26.32 (CH_2), 22.79 (CH_2), 17.60 (C*CH_3), 14.21 (CH_3).

Synthesis of compound 23:

Compound **23** was prepared from 4-iodophenol and (R)-1-((1-iodopropan-2-yl)oxy)octane. The product was purified by silica gel chromatography (PE:DCM (5:5) to give **23** as a colorless oil (26 %).

^{1}H NMR (300 MHz, $CDCl_3$) δ 7.58 – 7.49 (m, 2H, $CH_{aromatic}$), 6.73 – 6.64 (m, 2H, $CH_{aromatic}$), 3.94 (dd, J = 9.4, 5.7 Hz, 1H, C*CH_2), 3.85 – 3.70 (m, 2H, C*CH_2, C*H), 3.60 – 3.45 (m, 2H, OCH_2), 1.63 – 1.50 (m, 2H, CH_2), 1.38 – 1.20 (m, 13H, 5 CH_2, C*CH_3), 0.92 – 0.84 (m, 3H, CH_3).

^{13}C NMR (75 MHz, $CDCl_3$) δ 158.94 (C_{quat}-O), 138.25 (CH), 117.15 (CH), 82.88 (C_{quat}-I), 73.71 (C*H), 71.83 (C*CH_2), 69.73 (OCH_2), 31.95 (CH_2), 30.19 (CH_2), 29.54 (CH_2), 29.40 (CH_2), 26.24 (CH_2), 22.78 (CH_2), 17.42 (C*CH_3), 14.24 (CH_3).

Synthesis of ((R)-1-((1-iodopropan-2-yl)oxy)octane) alkane chain:[7]

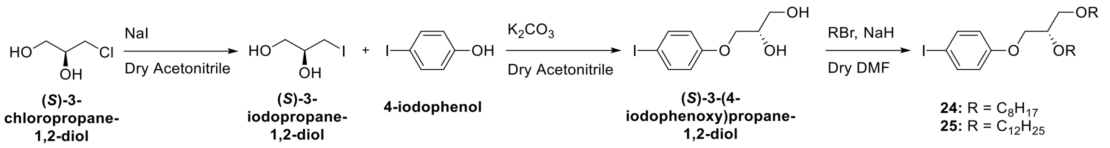

(S)-3-(4-iodophenoxy)propane-1,2-diol:

(S)-3-chloropropane-1,2-diol (4.5 mmol, 0.5 g) and NaI (45 mmol, 6.75 g) were dissolved in dry acetonitrile (40 mL). The reaction mixture was refluxed for 12 h. After cooling to room temperature, the mixture was filtered and wash with dry acetonitrile (60 mL). The product was filtered using a silica column and concentrated and reduced pressure. The compound *(S)*-3-iodopropane-1,2-diol was used without further purification.

K_2CO_3 (91 mmol, 12.58 g) and 4-iodophenol (9.1 mmol, 2 g) were dissolved in dry acetonitrile (100 mL). The mixture reaction was refluxed during 30 minutes. (S)-3-iodopropane-1,2-diol (21 mmol, 3.636 g) was added to the mixture and the reaction was refluxed for 4 days. After cooling to room temperature, the mixture was filtered and wash with acetonitrile. The filtrate was concentrated under reduced pressure. The product was purified by silica gel chromatography (DCM:AcOEt (8:2) – DCM:AcOEt (5:5) gradient in 30 minutes). The compound (S)-3-(4-iodophenoxy)propane-1,2-diol was obtained as yellow oil (0.428 g, 16 %).

^{1}H NMR (300 MHz, CD_3CN) δ 7.61 – 7.56 (m, 2H, $CH_{aromatic}$), 6.79 – 6.73 (m, 2H, $CH_{aromatic}$), 4.02 – 3.94 (m, 1H, C*CH), 3.96 – 3.84 (m, 2H, OCH_2), 3.64 – 3.47 (m, 2H, OCH_2), 3.26 – 3.20 (m, 1H, OH), 2.89 – 2.82 (m, 1H, OH).

^{13}C NMR (75 MHz, CD_3CN) δ 159.98 (C_{quat}), 139.24 (CH), 118.13 (CH), 83.08 (C_{quat}), 71.14 (C*CH), 70.36 (OCH_2), 63.80 (OCH_2).

Synthesis of compound 24:

Compound **24** was prepared from *(S)*-3-(4-iodophenoxy)propane-1,2-diol and 1-bromooctane. The base (K_2CO_3) has been changed for this reaction by NaH 60 % oil dispersion (6 eq.). The product was purified by silica gel chromatography (PE:DCM (5:5) to give **24** as a yellow pale oil (58 %).

^{1}H NMR (300 MHz, $CDCl_3$) δ 7.54 (d, *J* = 9.1 Hz, 2H, $CH_{aromatic}$), 6.70 (d, *J* = 9.2 Hz, 2H, $CH_{aromatic}$), 4.12 – 3.92 (m, 2H, OCH_2), 3.75 (p, *J* = 5.4 Hz, 1H, C*CH), 3.60 (t, *J* = 6.6 Hz, 2H, OCH_2), 3.56 (d, *J* = 5.7 Hz, 2H, OCH_2), 3.45 (t, *J* = 6.6 Hz, 2H, OCH_2), 1.62 – 1.50 (m, 4H, CH_2), 1.26 (broad s, 20H, CH_2), 0.88 (t, *J* = 6.8 Hz, 6H, CH_3).

^{13}C NMR (75 MHz, $CDCl_3$) δ 159.03 (C_{quat}), 138.31 (CH), 117.28 (CH), 82.94 (C_{quat}), 71.94 (OCH_2), 71.01 (OCH_2), 70.26 (OCH_2), 68.50 (OCH_2), 31.99 (2 CH_2), 30.19 (2 CH_2), 29.78 (2 CH_2), 29.57 (CH_2), 29.43 (CH_2), 26.28 (CH_2), 26.20 (CH_2), 22.81 (2 CH_2), 14.24 (CH_3).

Synthesis of compound 25:

Compound **25** was prepared from *(S)*-3-(4-iodophenoxy)propane-1,2-diol and 1-bromododecane. The base (K_2CO_3) has been changed for this reaction by NaH 60% oil dispersion (6 eq.). The product was purified by silica gel chromatography (PE:DCM (5:5) to give **25** as a yellow pale oil (67 %).

^{1}H NMR (300 MHz, $CDCl_3$) δ 7.54 (d, *J* = 9.1 Hz, 2H, $CH_{aromatic}$), 6.70 (d, *J* = 9.0 Hz, 2H, $CH_{aromatic}$), 4.12 – 3.91 (m, 2H, OCH_2), 3.75 (p, *J* = 5.4 Hz, 1H, C*CH), 3.60 (t, *J* = 6.7 Hz, 2H, OCH_2), 3.56 (d, *J* = 5.9 Hz, 2H, OCH_2), 3.45 (t, *J* = 6.6 Hz, 2H, OCH_2), 1.61 – 1.50 (s, 4H, CH_2), 1.25 (broad s, 36H, CH_2), 0.94 – 0.82 (m, 9H, CH_3).

^{13}C NMR (75 MHz, $CDCl_3$) δ 158.98 (C_{quat}), 138.28 (CH), 117.23 (CH), 82.93 (C_{quat}), 71.92 (OCH_2), 71.00 (OCH_2), 70.21 (OCH_2), 68.42 (OCH_2), 32.08 (2 CH_2), 30.17 (2 CH_2), 29.84 (2 CH_2), 29.80 (2 CH_2), 29.78 (6 CH_2), 29.62 (CH_2), 29.52 (CH_2), 26.27 (CH_2), 26.19 (CH_2), 22.85 (2 CH_2), 14.28 (2 CH_3).

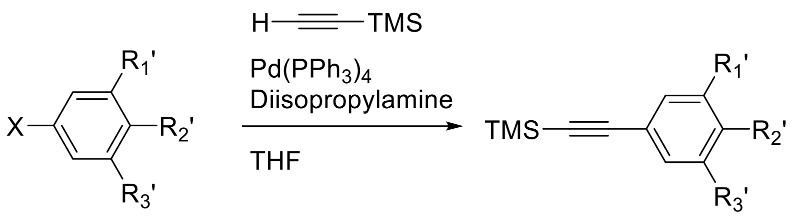

14-25 **26-37**

Position 3,4,5: R_1' = O-Alk, R_2' = O-Alk, R_3' = O-Alk, X = Br

Position 3,4: R_1' = O-Alk, R_2' = O-Alk, R_3' = H, X = Br

Position 4: R_1' = H, R_2' = O-Alk, R_3' = H, X = I

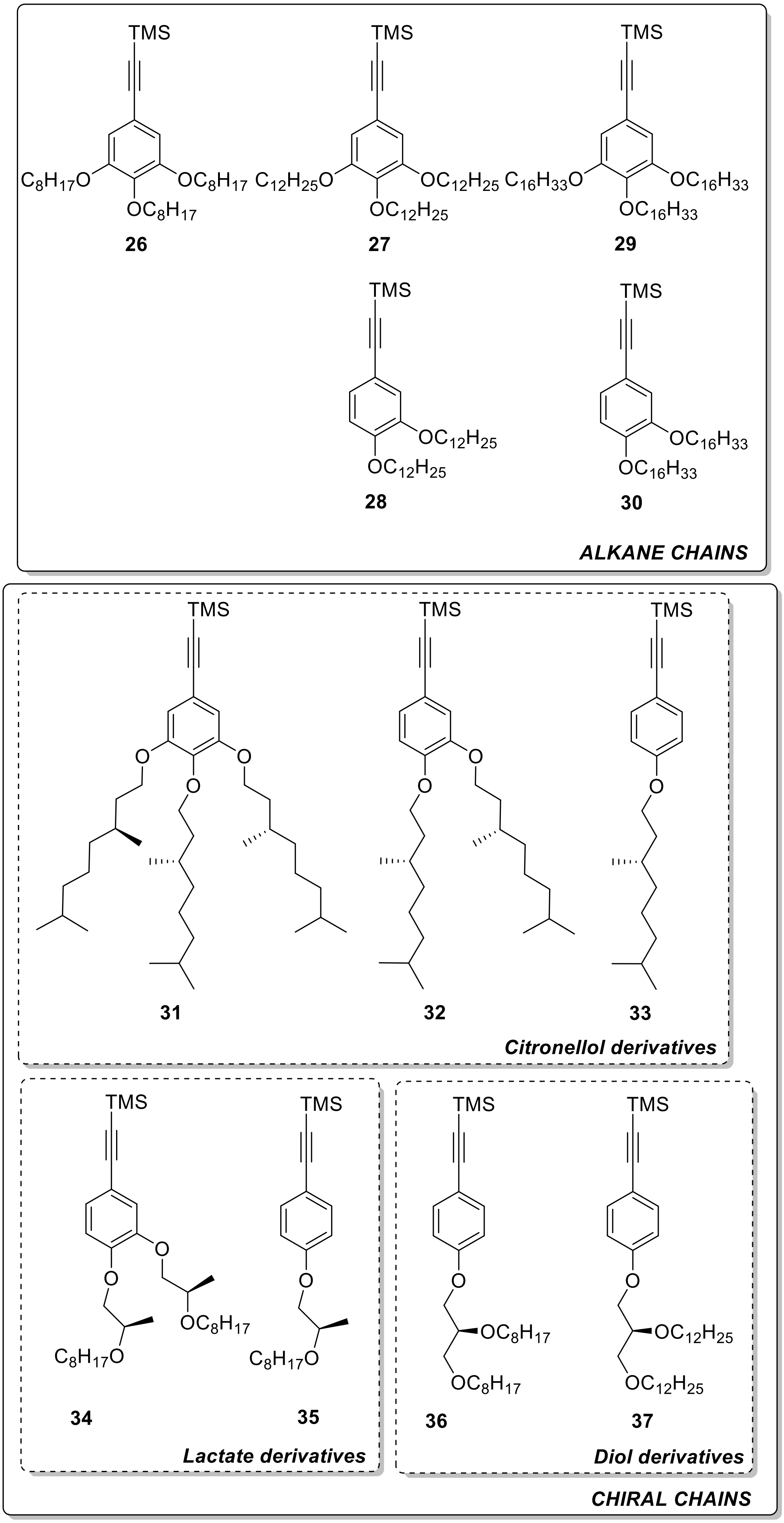

TMS
$C_8H_{17}O$
OC_8H_{17}
OC_8H_{17}
26
$C_{12}H_{25}O$
$OC_{12}H_{25}$
$OC_{12}H_{25}$
27
$C_{16}H_{33}O$
$OC_{16}H_{33}$
$OC_{16}H_{33}$
29
$OC_{12}H_{25}$
$OC_{12}H_{25}$
28
$OC_{16}H_{33}$
$OC_{16}H_{33}$
30
ALKANE CHAINS
31
32
33
Citronellol derivatives
OC_8H_{17}
$C_8H_{17}O$
34
35
Lactate derivatives
OC_8H_{17}
OC_8H_{17}
36
$OC_{12}H_{25}$
$OC_{12}H_{25}$
37
Diol derivatives
CHIRAL CHAINS

General procedure B:

Compounds **26-37** were prepared according General Procedure B. In a schlenk, compound **26-37** (1 eq.) were dissolved in propylamine (30 mL/g). The solution was degassed with nitrogen during 30 minutes. $Pd(PPh_3)_4$ (0.1 eq.) and TMS-acetylene (5 eq.) was added and the mixture was heated to 60 °C for 48 h. After cooling to room temperature, the solvent was removed under reduced pressure and the product was solubilized in DCM, washed with water, dried over $MgSO_4$ and concentrated under reduced pressure. The product was purified by silica gel chromatography.

Synthesis of compound 26:[8]
Compound **26** was prepared from 5-bromo-1,2,3-tris(octyloxy)benzene **14** and trimethysilylacetylene. The product was purified by silica gel chromatography (PE:DCM (5:5) and recrystallized by slow evaporation (CH_2Cl_2/MeOH) to give **26** as a white solid (78 %).
^{1}H NMR (300 MHz, $CDCl_3$) δ 6.66 (s, 2H, $CH_{aromatic}$), 3.95 (t, *J* = 6.5 Hz, 6H, OCH_2), 1.86 – 1.64 (m, 6H, CH_2), 1.48 – 1.29 (m, 6H, CH_2), 1.36 – 1.24 (m, 24H, CH_2), 0.94 – 0.83 (m, 9H, CH_3), 0.24 (s, 9H, $CH_{3\text{-TMS}}$). The analytical results are in good agreement with the data published in the literature. [8]

Synthesis of compound 27:[9]
Compound **27** was prepared from 5-bromo-1,2,3-tris(dodecyloxy)benzene **15** and trimethysilylacetylene. The product was purified by silica gel chromatography (PE – PE:DCM (5:5) gradient in 30 minutes) and recrystallized by slow evaporation (CH_2Cl_2/MeOH) to give **27** as a white solid (91 %).
^{1}H NMR (300 MHz, $CDCl_3$) δ 6.66 (s, 2H, $CH_{aromatic}$), 3.94 (t, *J* = 6.5 Hz, 6H, OCH_2), 1.86 – 1.64 (m, 6H, CH_2), 1.50 – 1.40 (m, 6H, CH_2), 1.26 (broad s, 48H, CH_2), 0.93 – 0.82 (m, 9H, CH_3), 0.24 (s, 9H, $CH_{3\text{-TMS}}$). The analytical results are in good agreement with the data published in the literature. [9]

Synthesis of compound 28:[6]
Compound **28** was prepared from 4-bromo-1,2-bis(dodecyloxy)benzene **16** and trimethysilylacetylene. The product was purified by silica gel chromatography (PE – PE:DCM (8:2) gradient in 30 minutes) and recrystallized by slow evaporation (CH_2Cl_2/MeOH) to give **28** as a white solid (79 %).
^{1}H NMR (300 MHz, $CDCl_3$) δ 7.03 (dd, *J* = 8.3, 1.9 Hz, 1H, $CH_{aromatic}$), 6.96 (d, *J* = 1.9 Hz, 1H, $CH_{aromatic}$), 6.76 (d, *J* = 8.3 Hz, 1H, $CH_{aromatic}$), 3.98 (t, *J* = 6.7 Hz, 2H, OCH_2), 3.98 (t, *J* = 6.7 Hz, 2H, OCH_2), 1.88 – 1.72 (m, 4H, CH_2), 1.51 – 1.39 (m, 4H, CH_2), 1.27 (broad s, 32H, CH_2), 0.94 – 0.79 (m, 6H, CH_3), 0.24 (s, 9H, $CH_{3\text{-TMS}}$).
^{13}C NMR (75 MHz, $CDCl_3$) δ 150.10 (C_{quat}), 148.80 (C_{quat}), 125.62 (CH), 117.26 (C_{quat}), 115.40 (CH), 113.35 (CH), 105.67 ($C_{quat\text{-}alkyne}$), 92.22 ($C_{quat\text{-}alkyne}$), 69.44 (OCH_2), 69.33 (OCH_2), 32.08 (CH_2), 29.85 (CH_2), 29.81 (CH_2), 29.78 (2 CH_2), 29.76 (CH2), 29.56 (CH_2), 29.52 (CH_2), 29.40 (CH_2), 29.36 (CH_2), 26.15 (CH_2), 22.84 (CH_2), 14.25 (CH_3), 0.23 ($CH_{3\text{-TMS}}$).

Synthesis of compound 29:[3]
Compound **29** was prepared from 5-bromo-1,2,3-tris(hexadecyloxy)benzene **17** and trimethysilylacetylene. The product was purified by silica gel chromatography (PE – PE:DCM (5:5) gradient in 30 minutes) and recrystallized by slow evaporation (CH_2Cl_2/MeOH) to give **29** as a white solid (64 %).
^{1}H NMR (300 MHz, $CDCl_3$) δ 6.66 (s, 2H, $CH_{aromatic}$), 3.95 (t, *J* = 6.5 Hz, 6H, OCH_2), 1.86 – 1.64 (m, 6H, CH_2), 1.50 – 1.40 (m, 6H, CH_2), 1.26 (broad s, 72H), 0.93 – 0.83 (m, 9H, CH_3), 0.24 (s, 9H, $CH_{3\text{-TMS}}$). The analytical results are in good agreement with the data published in the literature. [3]

Synthesis of compound 30:
Compound **30** was prepared from 4-bromo-1,2-bis(hexadecyloxy)benzene **18** and trimethysilylacetylene. The product was purified by silica gel chromatography (PE – PE:DCM (8:2)

gradient in 30 minutes) and recrystallized by slow evaporation (CH_2Cl_2/MeOH) to give **30** as a white solid (53 %).

^{1}H NMR (300 MHz, $CDCl_3$) δ 7.03 (dd, *J* = 8.3, 1.9 Hz, 1H, $CH_{aromatic}$), 6.96 (d, *J* = 1.9 Hz, 1H, $CH_{aromatic}$), 6.76 (d, *J* = 8.3 Hz, 1H, $CH_{aromatic}$), 3.98 (t, *J* = 6.6 Hz, 2H, OCH_2), 3.97 (t, *J* = 6.6 Hz, 2H, OCH_2), 1.80 (p, *J* = 6.6 Hz, 4H, CH_2), 1.50 – 1.40 (m, 4H, CH_2), 1.26 (broad s, 48H, CH_2), 0.93 – 0.83 (m, 6H, CH_3), 0.24 (s, 9H, $CH_{3\text{-TMS}}$).

^{13}C NMR (75 MHz, $CDCl_3$) δ 150.09 (C_{quat}), 148.80 (C_{quat}), 125.61 (CH), 117.26 (CH), 115.40 (C_{quat}), 113.34 (CH), 105.67 ($C_{quat\text{-}alkyne}$), 92.21 ($C_{quat\text{-}alkyne}$), 69.43 (OCH_2), 69.33 (OCH_2), 32.09 (CH_2), 29.87 (4 CH_2), 29.82 (CH_2), 29.78 (CH_2), 29.56 (CH_2), 29.52 (CH_2), 29.40 (CH_2), 29.36 (CH_2), 26.17 (CH_2), 26.15 (CH_2), 22.84 (CH_2), 14.25 (CH_3), 0.23 ($CH_{3\text{-TMS}}$).

Synthesis of compound 31:

Compound **31** was prepared from 5-bromo-1,2,3-tris(((S)-3,7-dimethyloctyl)oxy)benzene **19** and trimethysilylacetylene. The product was purified by silica gel chromatography (PE – PE:DCM (8:2) gradient in 30 minutes) to give **31** as a white solid (39 %).

^{1}H NMR (300 MHz, $CDCl_3$) δ 6.67 (s, 2H, CH), 4.07 – 3.90 (m, 6H, OCH_2), 1.91 – 1.76 (m, 3H, C*CH), 1.74 – 1.65 (m, 3H, C*CH), 1.65 – 1.43 (m, 6H, CH_2), 1.39 – 1.08 (m, 18H, CH_2), 0.93 (d, *J* = 6.5 Hz, 6H, C*-CH_3), 0.87 (d, *J* = 6.6 Hz, 3H, C*-CH_3), 0.86 (d, *J* = 6.6 Hz, 18H, CH_3), 0.24 (s, 9H, CH_3).

^{13}C NMR (75 MHz, $CDCl_3$) δ 153.04 (C_{quat}), 139.53 (C_{quat}), 117.66 (C_{quat}), 110.86 (CH), 105.67 (C_{quat}), 92.61 (C_{quat}), 71.79 (OCH_2), 67.55 (2 OCH_2), 39.52 (CH_2), 39.42 (2 CH_2), 37.66 (CH_2), 37.48 (2 CH_2), 36.51 (3 CH_2), 29.95 (2 C*H), 29.79 (C*H), 28.12 (3 CH), 24.87 (2 C*CH_2), 24.85 (C*CH_2), 22.84 (3 CH_3), 22.75 (2 C*CH_3), 22.74 (C*CH_3), 19.71 (3 CH_3), 0.17 (3 $CH_{3\text{-TMS}}$).

Synthesis of compound 32:

Compound **32** was prepared from 4-bromo-1,2-bis(((S)-3,7-dimethyloctyl)oxy)benzene **20** and trimethysilylacetylene. The product was purified by silica gel chromatography (PE – PE:DCM (8:2) gradient in 30 minutes) to give **32** as a white solid (77 %).

^{1}H NMR (300 MHz, $CDCl_3$) δ 7.03 (dd, *J* = 8.2, 1.9 Hz, 1H, $CH_{aromatic}$), 6.97 (d, *J* = 1.9 Hz, 1H, $CH_{aromatic}$), 6.77 (d, *J* = 8.3 Hz, 1H, $CH_{aromatic}$), 4.05 – 3.98 (m, 4H, OCH_2), 1.91 – 1.80 (m, 2H, C*-CH), 1.73 – 1.47 (m, 6H, C*-CH, CH_2), 1.41 – 1.09 (m, 12H, CH_2), 0.95 (d, *J* = 1.5 Hz, 3H, C*-CH_3), 0.93 (d, *J* = 1.5 Hz, 3H, C*-CH_3), 0.88 (d, *J* = 1.3 Hz, 6H, CH_3), 0.86 (d, *J* = 1.3 Hz, 6H, CH_3), 0.24 (s, 9H, $CH_{3\text{-TMS}}$).

^{13}C NMR (75 MHz, $CDCl_3$) δ 150.01 (C_{quat}), 148.74 (C_{quat}), 125.56 (CH), 116.97 (CH), 115.31 (C_{quat}), 113.11 (CH), 105.65 ($C_{quat\text{-}alkyne}$), 92.23 ($C_{quat\text{-}alkyne}$), 67.70 (OCH_2), 67.63 (OCH_2), 39.41 (2 CH_2), 37.50 (CH_2), 37.48 (CH_2), 36.33 (CH_2), 36.27 (CH_2), 30.08 (2 C*H), 28.14 (2 CH), 24.86 (2 C*CH_2), 22.85 (2 CH_3), 22.75 (2 C*CH_3), 19.86 (2 CH_3), 0.23 ($CH_{3\text{-TMS}}$).

Synthesis of compound 33:

Compound **33** was prepared from (S)-1-bromo-4-((3,7-dimethyloctyl)oxy)benzene **21** and trimethysilylacetylene. The product was purified by silica gel chromatography (PE – PE:DCM (8:2) gradient in 30 minutes) to give **33** as a white solid (37 %).

^{1}H NMR (300 MHz, $CDCl_3$) δ 7.40 (d, *J* = 8.9 Hz, 2H, $CH_{aromatic}$), 6.81 (d, *J* = 8.9 Hz, 2H, $CH_{aromatic}$), 4.04 – 3.93 (m, 2H, OCH_2), 1.91 – 1.75 (m, 1H, C*H), 1.74 – 1.44 (m, 3H, C*-CH_2), 1.41 – 1.10 (m, 6H, CH_2), 0.95 (d, *J* = 6.4 Hz, 3H, C*-CH_3), 0.89 (d, *J* = 6.6 Hz, 6H, CH_3), 0.26 (s, 9H, $CH_{3\text{-TMS}}$).

^{13}C NMR (75 MHz, $CDCl_3$) δ 159.50 (C_{quat}), 133.58 (2 CH), 115.22 (C_{quat}), 114.52 (2 CH), 105.52 ($C_{quat\text{-}alkyne}$), 92.38 ($C_{quat\text{-}alkyne}$), 66.53 (OCH_2), 39.40 (CH_2), 37.44 (CH_2), 36.28 (CH_2), 30.01 (C*H), 28.12 (CH), 24.80 (C*CH_2), 22.85 (CH_3), 22.75 (C*CH_3), 19.81 (CH_3), 0.24 ($CH_{3\text{-TMS}}$).

Synthesis of compound 34:

Compound **34** was prepared from 4-iodo-1,2-bis(*(R)*-2-(octyloxy)propoxy)benzene **22** and trimethysilylacetylene. The product was purified by silica gel chromatography (PE – PE:DCM (8:2) gradient in 30 minutes) to give **34** as a yellow pale solid (47 %).

^{1}H NMR (300 MHz, $CDCl_3$) δ 7.04 (dd, *J* = 8.3, 1.9 Hz, 1H, $CH_{aromatic}$), 6.98 (s, 1H, $CH_{aromatic}$), 6.78 (d, *J* = 8.3 Hz, 1H, $CH_{aromatic}$), 4.07 – 3.94 (m, 2H, C*H), 3.91 – 3.71 (m, 4H, C^*CH_2), 3.65 – 3.47 (m, 4H, OCH_2), 1.60 – 1.51 (m, 4H, CH_2), 1.35 – 1.25 (m, 26H, C^*CH_3, CH_2), 0.93 – 0.81 (m, 6H, CH_3), 0.24 (s, 9H, CH_{3-TMS}).
^{13}C NMR (75 MHz, $CDCl_3$) δ 149.89 (C_{quat}), 149.86 (C_{quat}), 148.63 (C_{quat}), 148.61 (C_{quat}), 125.86 (CH), 125.83 (CH), 117.60 (CH), 117.47 (CH), 115.85 (C_{quat}), 115.83 (C_{quat}), 113.68 (CH), 113.56 (CH), 105.41 ($C_{quat-alkyne}$), 92.44 ($C_{quat-alkyne}$), 73.96 (C*H), 73.12 (C^*CH_2), 73.06 (C^*CH_2), 73.03 (C^*CH_2), 72.96 (C^*CH_2), 69.97 (C^*CH_2), 69.95 (C^*CH_2), 69.91 (C^*CH_2), 69.88 (C^*CH_2), 31.99 (CH_2), 30.33 (CH_2), 29.62 (CH_2), 29.43 (CH_2), 26.32 (CH_2), 22.80 (CH_2), 17.72 (CH_2), 17.67 (C^*CH_3), 14.22 (CH_3), 0.19 (CH_{3-TMS}).

Synthesis of compound 35:
Compound **35** was prepared from *(R)*-1-iodo-4-(2-(octyloxy)propoxy)benzene **23** and trimethysilylacetylene. The product was purified by silica gel chromatography (PE – PE:DCM (5:5) gradient in 30 minutes) to give **35** as a yellow pale solid (81 %).
^{1}H NMR (300 MHz, $CDCl_3$) δ 7.41 – 7.36 (m, 2H, $CH_{aromatic}$), 6.84 – 6.79 (m, 2H, $CH_{aromatic}$), 4.00 – 3.95 (m, 1H, C^*CH_2), 3.87 – 3.82 (m, 1H, C^*CH_2), 3.82 – 3.71 (m, 1H, C*H), 3.59 – 3.47 (m, 1H, OCH_2), 1.61 – 1.52 (m, 2H, CH_2), 1.35 – 1.24 (m, 13H, CH_2, C^*CH_3), 0.93 – 0.83 (m, 3H, CH_3), 0.23 (s, 9H, CH_{3-TMS}).
^{13}C NMR (75 MHz, $CDCl_3$) δ 159.30 (C_{quat}), 133.58 (2 CH), 115.52 (C_{quat}), 114.65 (2 CH), 105.39 ($C_{quat-alkyne}$), 92.56 ($C_{quat-alkyne}$), 73.81 (C*H), 71.85 (OCH_2), 69.81 (C^*CH_2), 31.99 (CH_2), 30.25 (CH_2), 29.58 (CH_2), 29.43 (CH_2), 26.29 (CH_2), 22.81 (CH_2), 17.49 (C^*CH_3), 14.23 (CH_3), 0.22 (CH_{3-TMS}).

Synthesis of compound 36:
Compound **36** was prepared from (S)-1-(2,3-bis(octyloxy)propoxy)-4-iodobenzene **24** and trimethysilylacetylene. The product was purified by silica gel chromatography (PE – PE:DCM (5:5) gradient in 30 minutes) to give **36** as a yellow pale oil (97 %).
^{1}H NMR (300 MHz, $CDCl_3$) δ 7.41 – 7.36 (m, 2H, $CH_{aromatic}$), 6.86 – 6.80 (m, 2H, $CH_{aromatic}$), 4.12 – 4.07 (m, 1H, C^*CH_2), 4.03 – 3.98 (m, 1H, C^*CH_2), 3.81 – 3.71 (m, 1H, C*H), 3.61 (t, *J* = 6.7 Hz, 2H, OCH_2), 3.57 (d, *J* = 6.1 Hz, 2H, C^*CH_2), 3.45 (t, *J* = 6.6 Hz, 2H, OCH_2), 1.63 – 1.50 (m, 4H, CH_2), 1.26 (broad s, 20H, CH_2), 0.93 – 0.83 (m, 6H, CH_3), 0.23 (s, 9H, CH_{3-TMS}).
^{13}C NMR (75 MHz, $CDCl_3$) δ 159.29 (C_{quat}), 133.56 (2 CH), 115.54 (C_{quat}), 114.68 (2 CH), 105.40 ($C_{quat-alkyne}$), 92.54 ($C_{quat-alkyne}$), 71.94 (C^*CH_2), 71.04 (C^*CH_2), 70.35 (OCH_2), 68.41 (OCH_2), 31.99 (2 CH_2), 30.19 (2 CH_2), 29.79 (2 CH_2), 29.58 (CH_2), 29.43 (CH_2), 26.28 (CH_2), 26.21 (CH_2), 22.81 (2 CH_2), 14.23 (2 CH_3), 0.22 (CH_{3-TMS}).

Synthesis of compound 37:
Compound **37** was prepared from (S)-1-(2,3-bis(dodecyloxy)propoxy)-4-iodobenzene **25** and trimethysilylacetylene. The product was purified by silica gel chromatography (PE – PE:DCM (7:3) gradient in 30 minutes) to give **37** as a yellow pale oil (74 %).
^{1}H NMR (300 MHz, $CDCl_3$) δ 7.40 – 7.36 (m, 2H, $CH_{aromatic}$), 6.85 – 6.80 (m, 2H, $CH_{aromatic}$), 4.11 – 4.07 (m, 1H, C^*CH_2), 4.03 – 3.98 (m, 1H, C^*CH_2), 3.81 – 3.72 (m, 1H, C*CH), 3.61 (t, *J* = 6.7 Hz, 2H, OCH_2), 3.57 (d, *J* = 5.1 Hz, 2H, C^*CH_2), 3.45 (t, *J* = 6.6 Hz, 2H, OCH_2), 1.62 – 1.51 (m, 4H, CH_2), 1.26 (broad s, 36H, CH_2), 0.93 – 0.80 (m, 6H, CH_3), 0.23 (s, 9H, CH_{3-TMS}).

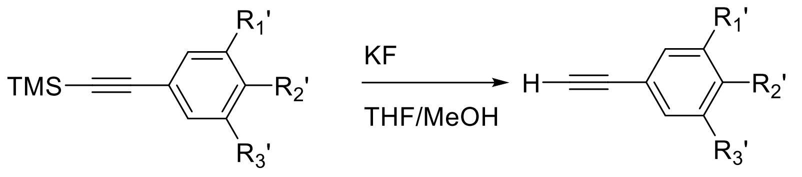

26-37 **38-49**

Position 3,4,5: R_1' = O-Alk, R_2' = O-Alk, R_3' = O-Alk, X = Br
Position 3,4: R_1' = O-Alk, R_2' = O-Alk, R_3' = H, X = Br
Position 4: R_1' = H, R_2' = O-Alk, R_3' = H, X = I

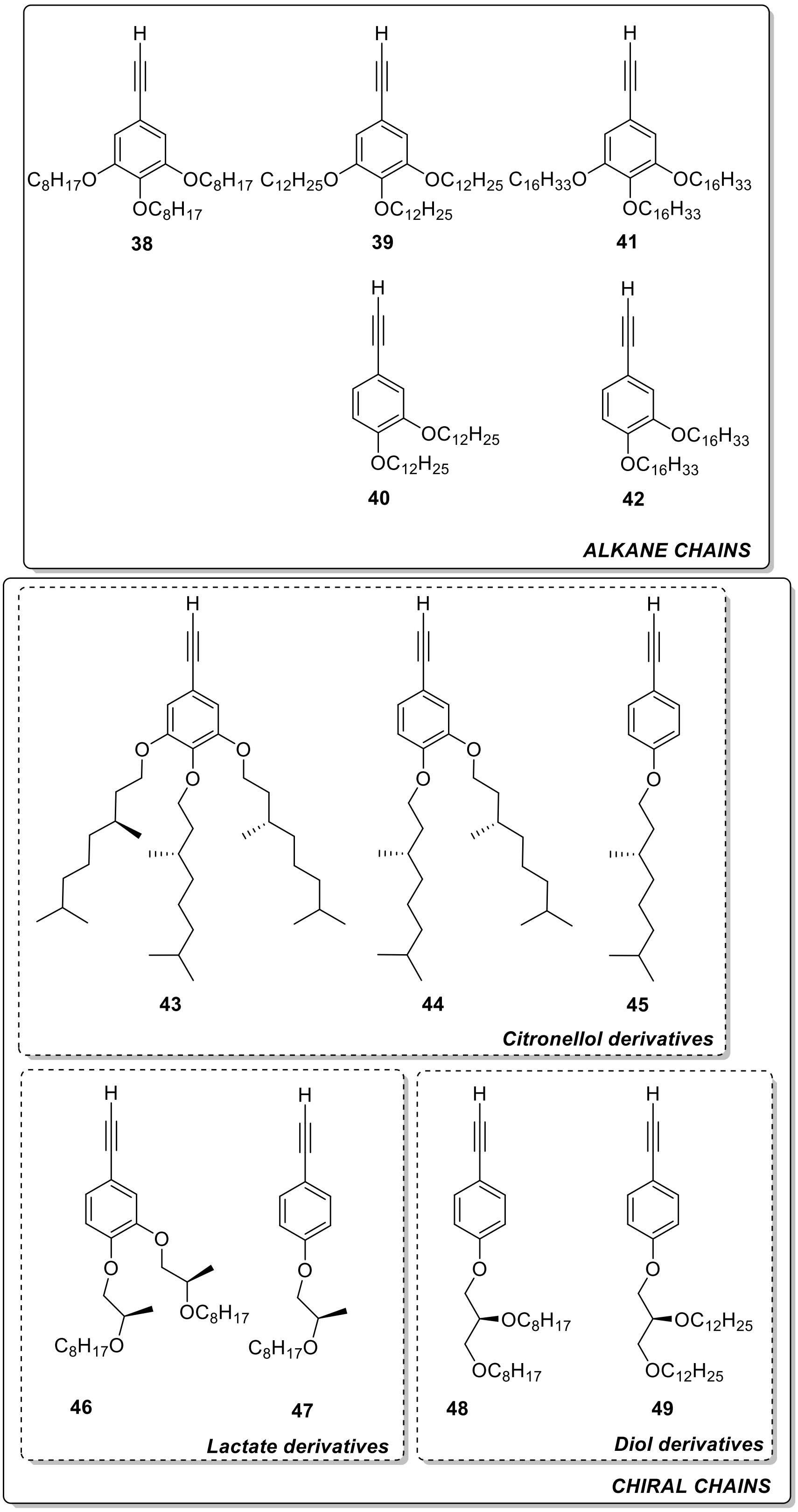

H
$C_8H_{17}O$
OC_8H_{17}
OC_8H_{17}
38
$C_{12}H_{25}O$
$OC_{12}H_{25}$
$OC_{12}H_{25}$
39
$C_{16}H_{33}O$
$OC_{16}H_{33}$
$OC_{16}H_{33}$
41
$OC_{12}H_{25}$
$OC_{12}H_{25}$
40
$OC_{16}H_{33}$
$OC_{16}H_{33}$
42
ALKANE CHAINS
O
43
44
45
Citronellol derivatives
$C_8H_{17}O$
OC_8H_{17}
46
47
Lactate derivatives
OC_8H_{17}
OC_8H_{17}
48
$OC_{12}H_{25}$
$OC_{12}H_{25}$
49
Diol derivatives
CHIRAL CHAINS

General procedure C:

Compound **38-49** were prepared according General Procedure C. Compound **38-49** and KF (5 eq.) were dissolved in a mixed of THF/MeOH (1/1). The mixture was stirred overnight at 40 °C. Solvents were removed under reduced pressure. The product obtained was dissolved in DCM, washed with water, dried over $MgSO_4$ and concentrated under reduced pressure. The product was purified by silica gel chromatography.

Synthesis of compound 38:[10]

Compound **38** was prepared from trimethyl((3,4,5-tris(octyloxy)phenyl)ethynyl)silane **26**. The product was purified by silica gel chromatography (PE – PE:DCM (5:5) gradient in 30 minutes) to give **38** as a colorless oil (96 %).

^{1}H NMR (300 MHz, $CDCl_3$) δ 6.69 (s, 2H, $CH_{aromatic}$), 4.02 – 3.90 (m, 6H, OCH_2), 2.99 (s, 1H, CH_{alkyne}), 1.87 – 1.65 (m, 6H, CH_2), 1.51 – 1.41 (m, 6H, CH_2), 1.36 – 1.23 (m, 24H, CH_2), 0.94 – 0.83 (m, 9H, CH_3). The analytical results are in good agreement with the data published in the literature. [10]

Synthesis of compound 39[10]

Compound **39** was prepared from trimethyl((3,4,5-tris(dodecyloxy)phenyl)ethynyl)silane **27**. The product was purified by silica gel chromatography (PE – PE:DCM (5:5) gradient in 30 minutes) and recrystallized by slow evaporation (CH_2Cl_2/MeOH) to give **39** as a white powder (97 %).

^{1}H NMR (300 MHz, THF) δ 6.69 (s, 2H, $CH_{aromatic}$), 3.95 (t, *J* = 6.3 Hz, 4H, OCH_2), 3.92 (t, *J* = 6.3 Hz, 2H, OCH_2), 3.38 (s, 1H, CH_{alkyne}), 1.85 – 1.62 (m, 6H, CH_2), 1.58 – 1.44 (m, 6H, CH_2), 1.30 (broad s, 48H, CH_2), 0.94 – 0.83 (m, 9H, CH_3). The analytical results are in good agreement with the data published in the literature.[10]

Synthesis of compound 40:[10]

Compound **40** was prepared from 3,4-bis(dodecyloxy)phenyl)ethynyl)trimethylsilane **28**. The product was purified by silica gel chromatography (PE – PE:DCM (5:5) gradient in 30 minutes) and recrystallized by slow evaporation (CH_2Cl_2/MeOH) to give **40** as a white powder (99 %).

^{1}H NMR (300 MHz, $CDCl_3$) δ 7.06 (dd, *J* = 8.2, 1.9 Hz, 1H, $CH_{aromatic}$), 6.99 (d, *J* = 1.9 Hz, 1H, $CH_{aromatic}$), 6.79 (d, *J* = 8.3 Hz, 1H, $CH_{aromatic}$), 3.98 (t, *J* = 6.6 Hz, 2H, OCH_2), 3.96 (t, *J* = 6.6 Hz, 2H, OCH_2), 2.98 (s, 1H, CH_{alkyne}), 1.89 – 1.73 (m, 4H, CH_2), 1.52 – 1.40 (m, 4H, CH_2), 1.27 (broad s, 32H, CH_2), 0.94 – 0.82 (m, 6H, CH_3). The analytical results are in good agreement with the data published in the literature.[10]

Synthesis of compound 41:[10]

Compound **41** was prepared from trimethyl((3,4,5-tris(hexadecyloxy)phenyl)ethynyl)silane **29**. The product was purified by silica gel chromatography (PE – PE:DCM (5:5) gradient in 30 minutes) and recrystallized by slow evaporation (CH_2Cl_2/MeOH) to give **41** as a white powder (99 %).

^{1}H NMR (300 MHz, $CDCl_3$) δ 7.06 (dd, *J* = 8.3, 1.9 Hz, 1H, $CH_{aromatic}$), 6.99 (d, *J* = 1.9 Hz, 1H, $CH_{aromatic}$), 6.79 (d, *J* = 8.3 Hz, 1H, $CH_{aromatic}$), 3.98 (, *J* = 6.6 Hz, 2H, OCH_2), 3.96 (, *J* = 6.6 Hz, 2H, OCH_2), 2.98 (s, 1H, CH_{alkyne}), 1.80 (dt, *J* = 8.3, 6.6 Hz, 4H, CH_2), 1.53 – 1.41 (m, 4H, CH_2), 1.27 (broad s, 32H, CH_2), 0.94 – 0.82 (m, 6H, CH_3). The analytical results are in good agreement with the data published in the literature.[10]

Synthesis of compound 42:[10]

Compound **42** was prepared from (3,4-bis(hexadecyloxy)phenyl)ethynyl)trimethylsilane **30**. The product was purified by silica gel chromatography (PE – PE:DCM (8:2) gradient in 30 minutes) and recrystallized by slow evaporation (CH_2Cl_2/MeOH) to give **42** as a white powder (91 %).

^{1}H NMR (300 MHz, $CDCl_3$) δ 7.06 (dd, *J* = 8.3, 1.9 Hz, 1H, $CH_{aromatic}$), 6.99 (d, *J* = 1.9 Hz, 1H, $CH_{aromatic}$), 6.79 (d, *J* = 8.3 Hz, 1H, $CH_{aromatic}$), 4.04 – 3.92 (m, 4H, OCH_2), 2.97 (s, 1H, CH_{alkyne}), 1.88 – 1.73 (m, 4H,

CH_2), 1.54 – 1.38 (m, 4H, CH_2), 1.26 (braod s, 48H, CH_2), 0.93 – 0.83 (m, 6H, CH_3). The analytical results are in good agreement with the data published in the literature.[10]

Synthesis of compound 43:
Compound **43** was prepared from trimethyl((3,4,5-tris(((S)-3,7-dimethyloctyl)oxy)phenyl)ethynyl) silane **31**. The product was purified by silica gel chromatography (PE – PE:DCM (8:2) gradient in 30 minutes) to give **43** as a colorless oil (0.320 g, 97 %).
^{1}H NMR (300 MHz, $CDCl_3$) δ 6.70 (s, 2H, $CH_{aromatic}$), 4.05 – 3.92 (m, 6H OCH_2), 2.99 (s, 1H, CH_{alkyne}), 1.93 – 1.74 (m, 3H, C*CH), 1.75 – 1.65 (m, 3H, CH), 1.64 – 1.47 (m, 6H, CH_2), 1.41 – 1.06 (m, 18H, CH_2), 0.95 – 0.91 (m, 9H, C*CH_3), 0.88 – 0.86 (m, 18H, CH_3).

Synthesis of compound 44:
Compound **44** was prepared from ((3,4-bis(((S)-3,7-dimethyloctyl)oxy)phenyl)ethynyl)trimethylsilane **32**. The product was purified by silica gel chromatography (PE – PE:DCM (8:2) gradient in 30 minutes) to give **44** as a colorless oil (88 %).
^{1}H NMR (300 MHz, $CDCl_3$) δ 7.00 (d, *J* = 1.9 Hz, 1H, $CH_{aromatic}$), 6.79 (d, *J* = 8.3 Hz, 1H, $CH_{aromatic}$), 4.12 – 3.92 (m, 4H, OCH_2), 2.98 (s, 1H, CH_{alkyne}), 1.93 – 1.78 (m, 2H, C*H), 1.75 – 1.46 (m, 6H, 2 CH, 2 CH_2), 1.38 – 1.09 (m, 12H, CH_2), 0.94 (d, *J* = 6.3 Hz, 6H, C*CH_3), 0.89 (d, *J* = 6.6 Hz, 12H, CH_3), 0.85 (d, *J* = 6.6 Hz, 12H, CH_3).

Synthesis of compound 45:
Compound **45** was prepared from (S)-((4-((3,7-dimethyloctyl)oxy)phenyl)ethynyl)trimethylsilane **33**. The product was purified by silica gel chromatography (PE – PE:DCM (8:2) gradient in 30 minutes) to give **45** as a colorless oil (84 %).
^{1}H NMR (300 MHz, $CDCl_3$)δ 7.42 (d, *J* = 9.0 Hz, 2H, $CH_{aromatic}$), 6.84 (d, *J* = 9.0 Hz, 2H, $CH_{aromatic}$), 4.05 – 3.94 (m, 2H, OCH_2), 2.99 (s, 1H, CH_{alkyne}), 1.91 – 1.75 (m, 1H, C*H), 1.74 – 1.46 (m, 3H, CH_3), 1.44 – 1.08 (m, 6H, CH_2), 0.95 (d, *J* = 6.4 Hz, 3H, C*CH_3), 0.89 (d, *J* = 6.6 Hz, 6H, CH_3).

Synthesis of compound 46:
Compound **46** was prepared from ((3,4-bis((R)-2-(octyloxy)propoxy)phenyl)ethynyl)trimethylsilane **34**. The product was purified by silica gel chromatography (PE – PE:DCM (5:5) gradient in 30 minutes) to give **46** as a colorless oil (78 %).
^{1}H NMR (300 MHz, $CDCl_3$)δ 7.06 (dd, *J* = 8.3, 1.9 Hz, 1H, $CH_{aromatic}$), 7.01 – 6.99 (m, 1H, $CH_{aromatic}$), 6.80 (dd, *J* = 8.3, 0.9 Hz, 1H, $CH_{aromatic}$), 4.08 – 3.94 (m, 2H, C*H), 3.90 – 3.72 (m, 4H, C*CH_2), 3.65 – 3.48 (m, 4H, OCH_2), 2.98 (s, 1H, CH_{alkyne}), 1.60 – 1.51 (m, 4H, CH_2), 1.40 – 1.18 (m, 26H, CH_2, C*CH_3), 0.93 – 0.80 (m, 6H, CH_3).
^{13}C NMR (75 MHz, $CDCl_3$) δ 150.05 (C_{quat}), 150.02 (C_{quat}), 148.66 (C_{quat}), 148.64 (C_{quat}), 125.96 (CH), 125.93 (CH), 117.67 (CH), 117.54 (CH), 114.66 (C_{quat}), 114.63 (C_{quat}), 113.63 (CH), 113.50 (CH), 83.89 ($C_{quat\text{-}alkyne}$), 75.75 (CH_{alkyne}), 73.94 (C*H), 73.91 (C*H), 73.09 (OCH_2), 73.03 (OCH_2), 72.96 (OCH_2), 72.89 (OCH_2), 70.00 (C*CH_2), 69.98 (C*CH_2), 69.93 (C*CH_2), 69.91 (C*CH_2), 31.99 (CH_2), 30.32 (CH_2), 29.63 (CH_2), 29.44 (CH_2), 26.32 (CH_2), 22.81 (CH_2), 17.68 (C*CH_3), 14.24 (CH_3).

Synthesis of compound 47:
Compound **47** was prepared from (R)-trimethyl((4-(2(octyloxy)propoxy)phenyl)ethynyl)silane **35**. The product was purified by silica gel chromatography (PE – PE:DCM (5:5) gradient in 30 minutes) to give **47** as a colorless oil (98 %).
^{1}H NMR (300 MHz, THF) δ 7.37 – 7.32 (m, 2H, $CH_{aromatic}$), 6.89 – 6.84 (m, 2H, $CH_{aromatic}$), 4.00 – 3.95 (m, 1H, C*CH_2), 3.86 – 3.81 (m, 1H, C*CH_2), 3.77 – 3.68 (m, 1H, C*H), 3.57 – 3.45 (m, 2H, OCH_2), 3.33 (s, 1H, CH_{alkyne}), 1.60 – 1.45 (m, 2H, CH_2), 1.30 (broad s, 10H, CH_2), 1.20 (d, *J* = 6.2 Hz, 3H, C*CH_3), 0.93 – 0.83 (m, 3H, CH_3).

^{13}C NMR (75 MHz, $CDCl_3$) δ 159.46 (C_{quat}), 133.66 (2 CH), 114.73 (2 CH), 114.40 (C_{quat}), 83.80 (C_{quat}), 75.88 (CH_{alkyne}), 73.76 (C*H), 71.83 (OCH_2), 69.76 ($C*CH_2$), 31.96 (CH_2), 30.22 (CH_2), 29.56 (CH_2), 29.40 (CH_2), 26.26 (CH_2), 22.78 (CH_2), 17.44 ($C*CH_3$), 14.20 (CH_3).

Synthesis of compound 48:
Compound **48** was prepared from (S)-((4-(2,3-bis(octyloxy)propoxy)phenyl)ethynyl)trimethylsilane **36**. The product was purified by silica gel chromatography (PE – PE:DCM (5:5) gradient in 30 minutes) to give **48** as a colorless oil (99 %).
^1H NMR (300 MHz, $CDCl_3$) δ 7.46 – 7.36 (m, 2H, $CH_{aromatic}$), 6.94 – 6.81 (m, 2H, $CH_{aromatic}$), 4.13 – 4.08 (m, 1H, $C*CH_2$), 4.04 – 3.99 (m, 1H, $C*CH_2$), 3.82 – 3.72 (m, 1H, C*H), 3.61 (t, J = 6.6 Hz, 2H, OCH_2), 3.57 (d, J = 5.1 Hz, 2H, $C*CH_2$), 3.46 (t, J = 6.6 Hz, 2H, OCH_2), 2.98 (s, 1H, CH_{alkyne}), 1.62 – 1.51 (m, 4H, CH_2), 1.26 (broad s, 20H, CH_2), 0.93 – 0.81 (m, 6H, CH_3).

Synthesis of compound 49:
Compound **49** was prepared from (S)-((4-(2,3-bis(dodecyloxy)propoxy)phenyl)ethynyl)triméthylsilane ***37***. The product was purified by silica gel chromatography (PE – PE:DCM (5:5) gradient in 30 minutes) to give **49** as a colorless oil (73 %).
^1H NMR (300 MHz, $CDCl_3$) δ 7.44 – 7.38 (m, 2H, $CH_{aromatic}$), 6.88 – 6.83 (m, 2H, $CH_{aromatic}$), 4.13 – 4.08 (m, 1H, $C*CH_2$), 4.04 – 3.99 (m, 1H, $C*CH_2$), 3.80 – 3.73 (m, 1H, C*H), 3.61 (t, J = 6.6 Hz, 2H, OCH_2), 3.57 (d, J = 6.1 Hz, 2H, $C*CH_2$), 3.45 (t, J = 6.6 Hz, 2H, OCH_2), 2.98 (s, 1H, CH_{alkyne}), 1.62 – 1.51 (m, 4H, CH_2), 1.26 (broad s, 36H, CH_2), 0.93 – 0.83 (m, 6H, CH_3).

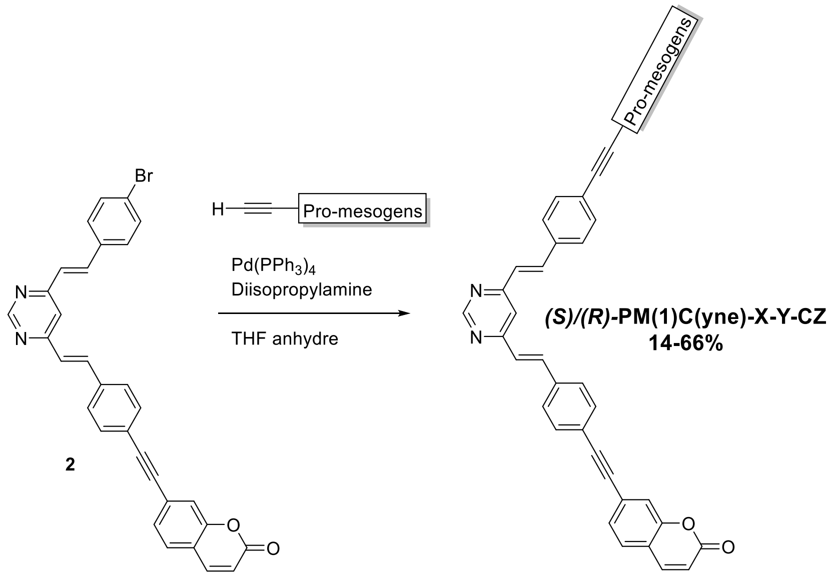

General procedure D:

In a Schlenk under inert atmosphere, compound **2** (1 eq.) and alkyne **38-49** (1.2 equiv) were dissolved in dry THF (20 mL/100 mg) and diisopropylamine (10 mL/100 mg). The solution was degassed with nitrogen for 30 minutes. $Pd(PPh_3)_4$ (0.1 eq.) was added and the mixture was heated to 60 °C for 72 h. After cooling to room temperature, the solvents were removed under reduced pressure and the product was solubilized in DCM, washed with water, dried over $MgSO_4$ and concentrated under reduced pressure. The product was purified by silica gel chromatography. The product obtained was recrystallized by slow evaporation of a mixture DCM/MeOH affording the targeted compound as yellow powder.

Synthesis of compound 10 (3,4,5-C8):

Compound **10** was prepared from 5-ethynyl-1,2,3-tris(octyloxy)benzene **38** and 7- ((4-((E)-2-(6-((E)-4-bromostyryl)pyrimidin-4-yl)vinyl)phenyl)ethynyl)-2H-chromen-2-one **2**. The product was purified by silica gel chromatography (DCM – DCM: AcOEt (95:5) gradient in 30 minutes) to give compound **10** (54 %).

^{1}H NMR (300 MHz, $CDCl_3$) δ 9.12 (d, *J* = 1.2 Hz, 1H, CH_{PM}), 7.92 (d, *J* = 15.9 Hz, 1H, $CH_{ethylenic}$), 7.91 (d, *J* = 15.9 Hz, 1H, $CH_{ethylenic}$), 7.69 (dd, *J* = 9.5, 0.7 Hz, 1H, $CH_{aromatic}$), 7.65 – 7.40 (m, 11H, $CH_{aromatic}$), 7.29 (d, *J* = 1.3 Hz, 1H, CH_{PM}), 7.12 (d, *J* = 15.9 Hz, 1H, $CH_{ethylenic}$), 7.09 (d, *J* = 15.9 Hz, 1H, $CH_{ethylenic}$), 6.75 (s, 2H, $CH_{aromatic}$), 6.43 (d, *J* = 9.5 Hz, 1H, $CH_{aromatic}$), 3.99 (t, *J* = 6.6, 2.0 Hz, 6H, OCH_2), 1.88 – 1.68 (m, 6H, CH_2), 1.54 – 1.42 (m, 6H, CH_2), 1.28 (broad s, 24H, CH_2), 0.95 – 0.81 (m, 9H, CH_3).

^{13}C NMR (75 MHz, $CDCl_3$) δ 162.87 (C_{quat}), 162.61 (C_{quat}), 160.45 (C_{quat}), 158.94 (CH), 154.03 (C_{quat}), 153.23 (C_{quat}), 142.84 (CH), 139.61 (C_{quat}), 136.56 (C_{quat}), 136.44 (CH), 136.21 (CH), 135.49 (C_{quat}), 132.46 (CH), 132.14 (CH), 127.92 (CH), 127.84 (CH), 127.81 (CH), 127.77 (CH), 127.12 (CH), 126.95 (C_{quat}), 126.50 (CH), 124.61 (C_{quat}), 123.41 (C_{quat}), 119.74 (CH), 118.99 (C_{quat}), 117.53 (C_{quat}), 117.18 (CH), 116.94 (CH), 110.47 (CH), 92.90 ($C_{quat-alkyne}$), 91.99 ($C_{quat-alkyne}$), 89.97 ($C_{quat-alkyne}$), 88.15 ($C_{quat-alkyne}$), 73.74 (OCH_2), 69.39 (OCH_2), 32.06 (CH_2), 31.99 (CH_2), 30.48 (CH_2), 29.69 (CH_2), 29.51 (CH_2), 29.43 (CH_2), 26.24 (CH_2), 22.84 (CH_2), 22.82 (CH_2), 14.23 (CH_3). **Anal. calcd** for [$C_{63}H_{72}N_2O_5$]: C, 80.69; H, 7.43; N, 2.80 found: C, 80.73; H, 7.74; N, 2.99.

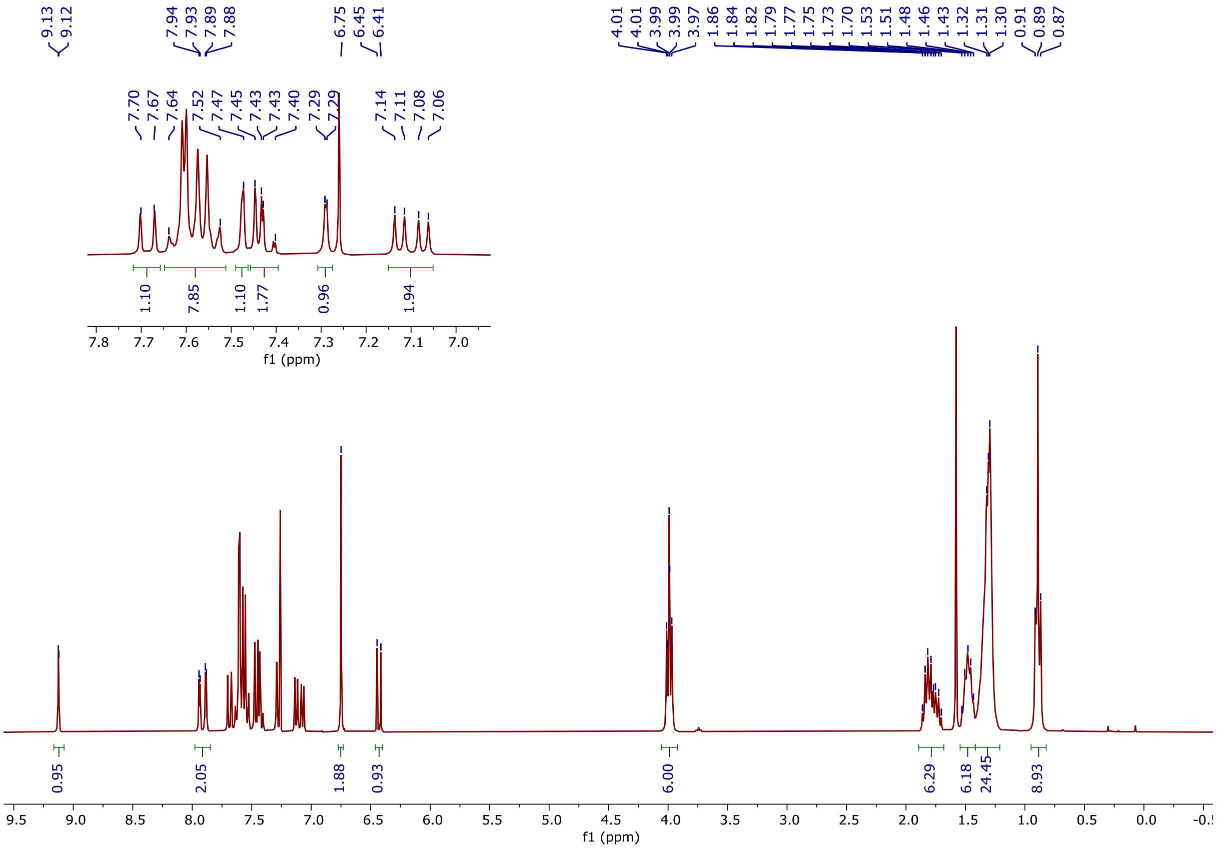

^{1}H NMR (300 MHz, $CDCl_3$) spectrum of compound **10**.

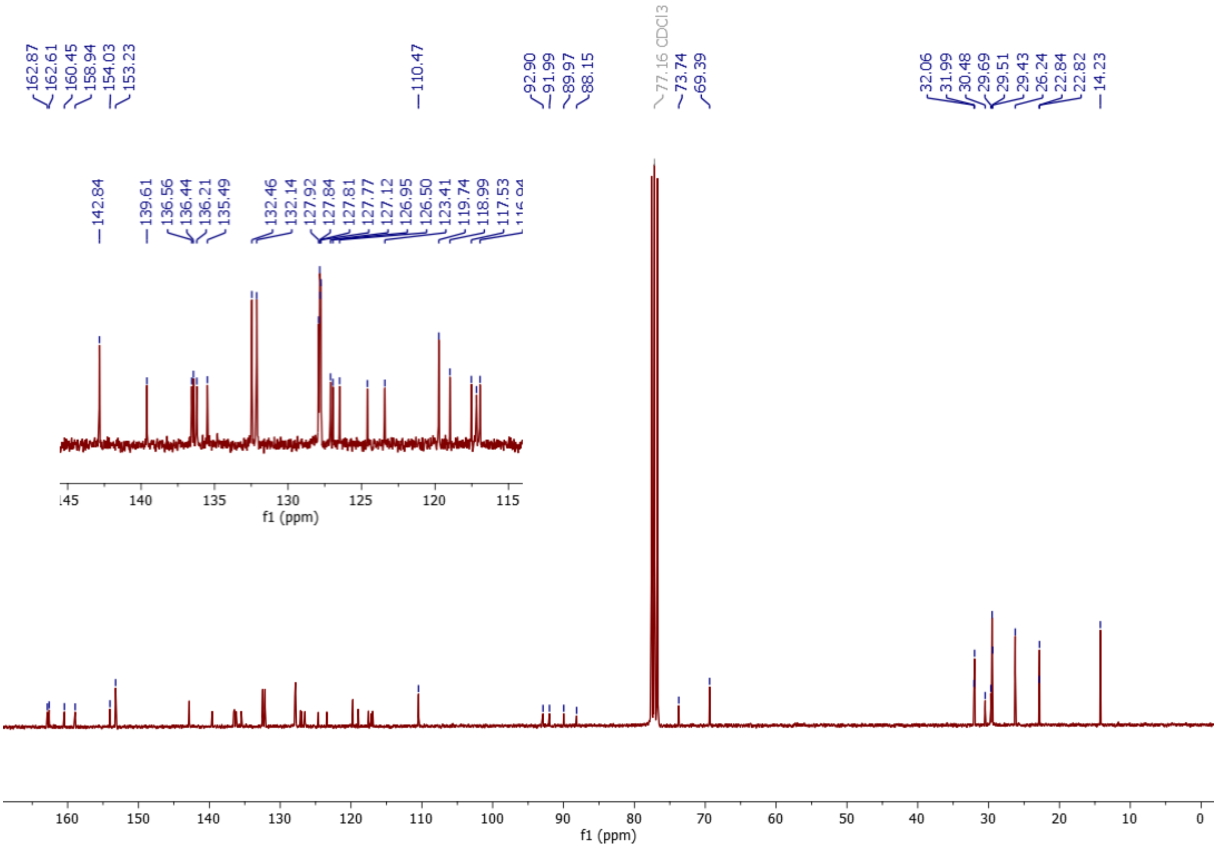

^{13}C NMR (75 MHz, $CDCl_3$) spectrum of compound **10**.

Synthesis of compound 11 (3,4,5-C12):

Compound **11** was prepared from 1,2,3-tris(dodecyloxy)-5-ethynylbenzene **39** and 7-((4-((E)-2-(6-((E)-4-bromostyryl)pyrimidin-4-yl)vinyl)phenyl)ethynyl)-2H-chromen-2-one **2**. The product was purified by silica gel chromatography (DCM – DCM: AcOEt (95:5) gradient in 30 minutes) to give compound **11** (62 %).

^{1}H NMR (300 MHz, $CDCl_3$) δ 9.14 (d, *J* = 1.2 Hz, 1H, CH_{PM}), 7.93 (d, *J* = 16.0, 1H, $CH_{ethylenic}$), 7.92 (d, *J* = 16.0, 1H, $CH_{ethylenic}$), 7.70 (dd, *J* = 9.5, 0.6 Hz, 1H, $CH_{aromatic}$), 7.66 – 7.41 (m, 11H, $CH_{aromatic}$), 7.30 (d, *J* = 1.3 Hz, 1H, CH_{PM}), 7.12 (d, *J* = 15.9, 1H, $CH_{ethylenic}$), 7.10 (d, *J* = 15.9, 1H, $CH_{ethylenic}$), 6.77 (s, 2H, $CH_{aromatic}$), 6.44 (d, *J* = 9.5 Hz, 1H, $CH_{aromatic}$), 4.01 (t, *J* = 6.5 Hz, 6H, OCH_2), 1.91 – 1.70 (m, 6H, CH_2), 1.56 – 1.44 (m, 6H, CH_2), 1.28 (broad s, 48H, CH_2), 0.97 – 0.82 (m, 9H, CH_3).

^{13}C NMR (75 MHz, $CDCl_3$) δ 162.87 (C_{quat}), 162.62 (C_{quat}), 160.45 (C_{quat}), 158.94 (CH), 154.03 (C_{quat}), 153.23 (C_{quat}), 142.84 (CH), 139.61 (C_{quat}), 136.56 (C_{quat}), 136.44 (CH), 136.21 (CH), 135.49 (C_{quat}), 132.46 (CH), 132.14 (CH), 127.92 (CH), 127.85 (CH), 127.82 (CH), 127.77 (CH), 127.12 (CH), 126.95 (C_{quat}), 126.50 (CH), 124.61 (C_{quat}), 123.42 (C_{quat}), 119.75 (CH), 118.99 (C_{quat}), 117.53 (C_{quat}), 117.18 (CH), 116.94 (CH), 110.48 (CH), 92.90 ($C_{quat-alkyne}$), 91.99 ($C_{quat-alkyne}$), 89.97 ($C_{quat-alkyne}$), 88.15 ($C_{quat-alkyne}$), 73.73 (OCH_2), 69.39 (OCH_2), 32.10 (CH_2), 32.08 (CH_2), 30.49 (CH_2), 29.90 (CH_2), 29.85 (CH_2), 29.81 (CH_2), 29.80 (CH_2), 29.75 (CH_2), 29.55 (CH_2), 29.52 (CH_2), 26.25 (CH_2), 22.84 (CH_2), 14.25 (CH_3).

Anal. calcd for [$C_{75}H_{96}N_2O_5$]: 81.48; H, 8.75; N, 2.53 found: C, 81.93; H, 8.46; N, 2.42.

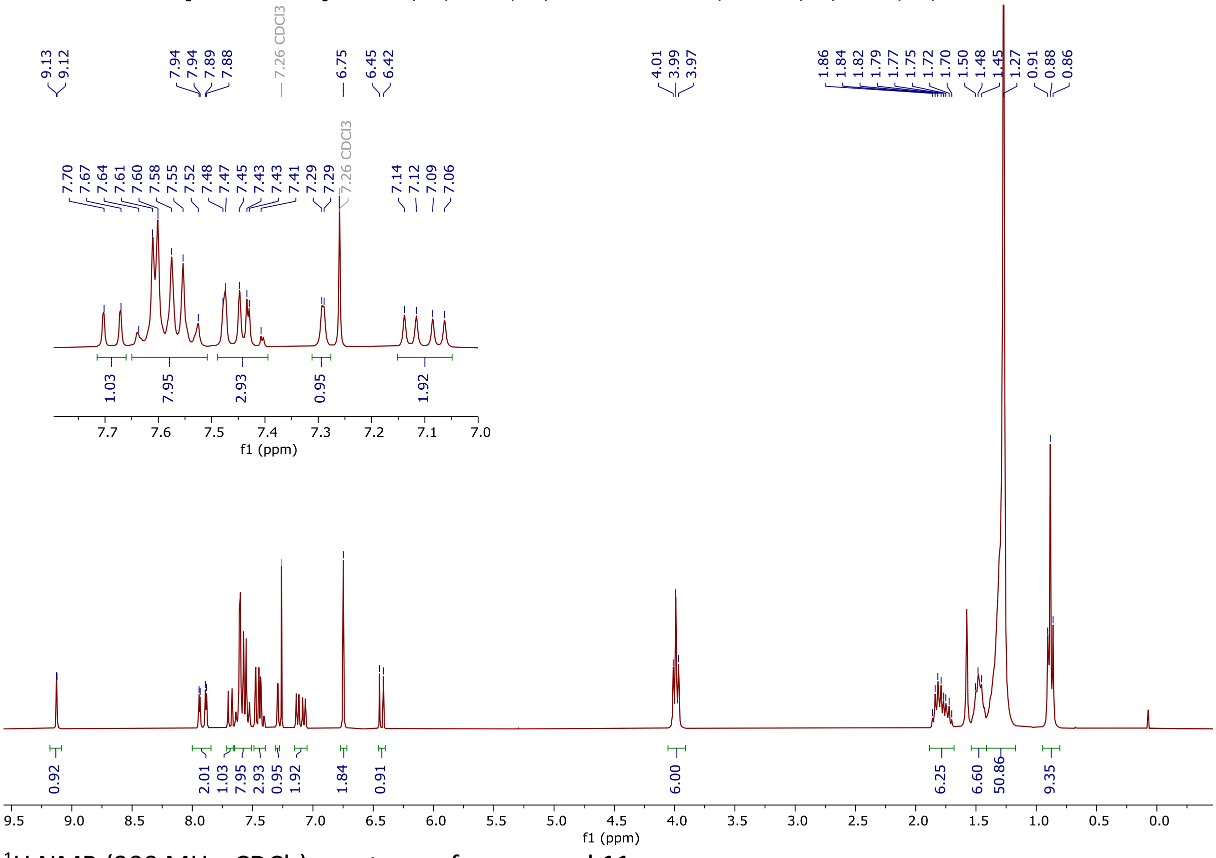

^{1}H NMR (300 MHz, $CDCl_3$) spectrum of compound **11**.

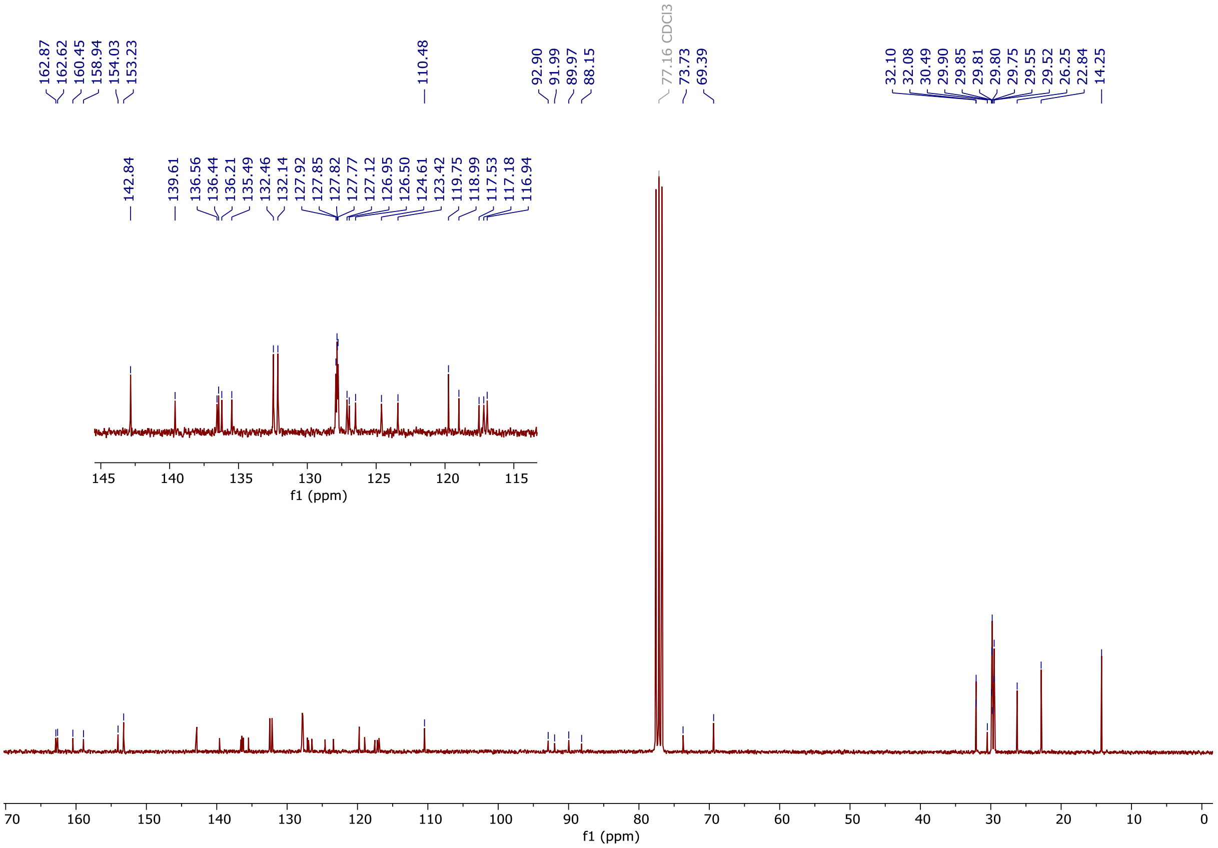

^{13}C NMR (75 MHz, $CDCl_3$) spectrum of compound **11**.

Synthesis of compound 12 (3,4-C12):

Compound **PM(1)C(yne)-3,4-C12** was prepared from 1,2-bis(dodecyloxy)-4-ethynylbenzene **40** and 7-((4-((E)-2-(6-((E)-4-bromostyryl)pyrimidin-4-yl)vinyl)phenyl)ethynyl)-2H-chromen-2-one **2**. The product was purified by silica gel chromatography (DCM – DCM: AcOEt (95:5) gradient in 30 minutes) and recrystallized by slow evaporation (CH_2Cl_2/MeOH) to give compound **12** (41 %).

^{1}H NMR (300 MHz, $CDCl_3$) δ 9.12 (d, *J* = 1.2 Hz, 1H, CH_{PM}), 7.91 (d, *J* = 16.0, 1H, $CH_{ethylenic}$), 7.90 (d, *J* = 16.0, 1H, $CH_{ethylenic}$), 7.68 (dd, *J* = 9.5, 0.7 Hz, 1H, $CH_{aromatic}$), 7.64 – 7.39 (m, 11H, $CH_{aromatic}$), 7.28 (d, *J* = 1.3 Hz, 1H, CH_{PM}), 7.15 – 7.02 (m, 4H, 2 $CH_{ethylenic}$, 2 $CH_{aromatic}$), 6.84 (d, *J* = 8.4 Hz, 1H, $CH_{aromatic}$), 6.42 (d, *J* = 9.5 Hz, 1H, $CH_{aromatic}$), 4.01 (t, *J* = 6.6 Hz, 4H, OCH2), 1.92 – 1.76 (m, 4H, CH2), 1.55 – 1.40 (m, 4H, CH_2), 1.27 (broad s, 32H, CH_2), 0.95 – 0.80 (m, 6H, CH_3).

^{13}C NMR (75 MHz, $CDCl_3$) δ 162.91 (C_{quat}), 162.59 (C_{quat}), 160.49 (C_{quat}), 158.94 (CH), 154.02 (C_{quat}), 150.14 (C_{quat}), 148.96 (C_{quat}), 142.88 (CH), 136.63 (CH), 136.44 (C_{quat}), 136.20 (CH), 135.28 (CH), 132.47 (CH), 132.06 (CH), 127.93 (CH), 127.85 (CH), 127.83 (CH), 127.77 (CH), 127.12 (CH), 126.96 (C_{quat}), 126.35 (CH), 125.26 (CH), 124.87 (C_{quat}), 123.40 (C_{quat}), 119.76 (CH), 118.99 (C_{quat}), 117.19 (CH), 116.95 (CH), 116.89 (CH), 115.21 (C_{quat}), 113.45 (CH), 92.90 ($C_{quat-alkyne}$), 91.99 ($C_{quat-alkyne}$), 89.96 ($C_{quat-alkyne}$), 88.11 ($C_{quat-alkyne}$), 69.48 (OCH_2), 69.34 (OCH_2), 32.09 (CH_2), 29.86 (CH_2), 29.82 (CH_2), 29.79 (CH_2), 29.78 (CH_2), 29.57 (CH_2), 29.52 (CH_2), 29.40 (CH_2), 29.36 (CH_2), 26.19 (CH_2), 26.17 (CH_2), 22.85 (CH_2), 14.27 (CH_3).

Anal. calcd for [$C_{63}H_{72}N_2O_4$.1/2 CH_2Cl_2]: C, 79.14; H, 7.64; N, 3.68 found: C, 80.37; H, 7.93; N, 2.71.

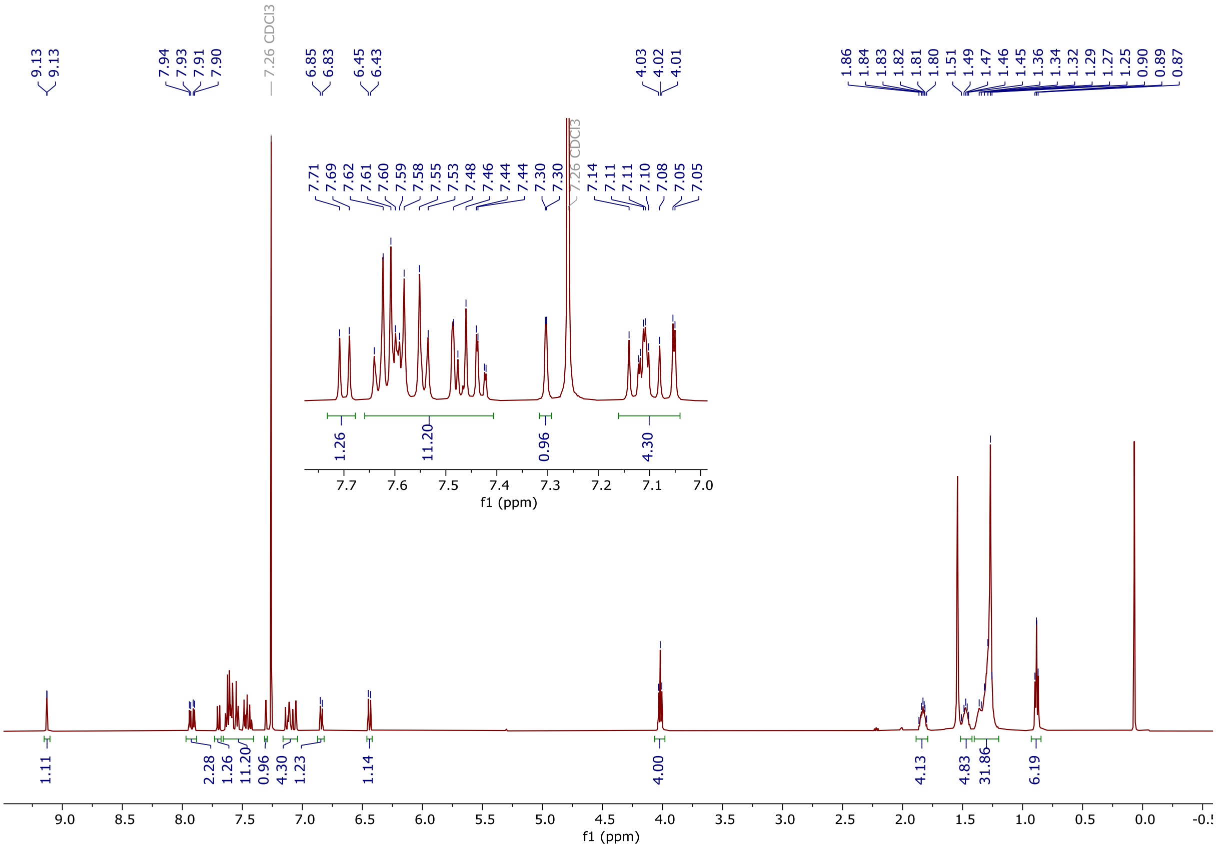

^{1}H NMR (300 MHz, $CDCl_3$) spectrum of compound **12**.

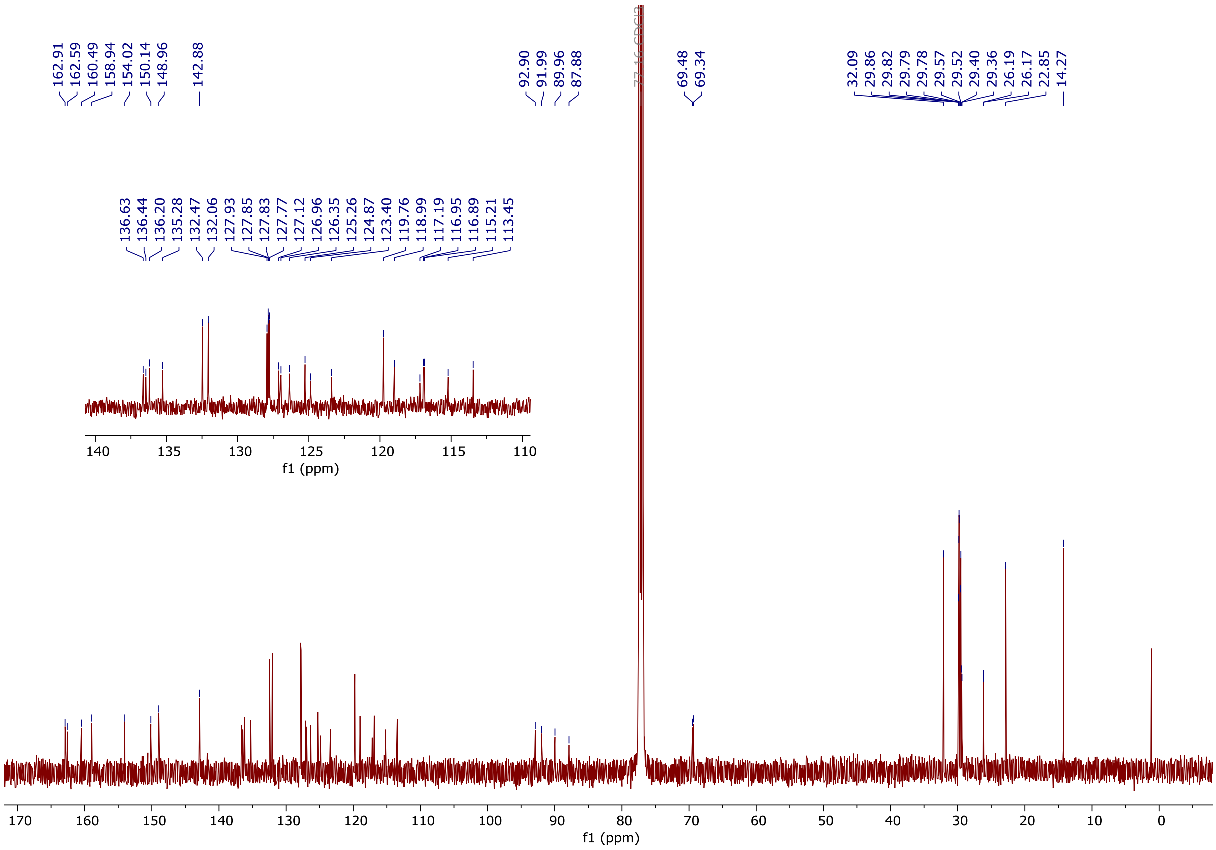

^{13}C NMR (75 MHz, $CDCl_3$) spectrum of compound **12**.

Synthesis of compound* PMC-3,4,5-C16 *(3,4,5-C16):

Compound **PM(1)C(yne)-3,4,5-C16** was prepared from 5-ethynyl-1,2,3-tris(hexadecyloxy)benzene **41** and 7-((4-((E)-2-(6-((E)-4-bromostyryl)pyrimidin-4-yl)vinyl)phenyl)ethynyl)-2H-chromen-2-one **2**. The product was purified by silica gel chromatography (DCM – DCM: AcOEt (95:5) gradient in 30 minutes) and recrystallized by slow evaporation (CH_2Cl_2/MeOH) to give compound **PMC-3,4,5-C16** (55 %).

^{1}H NMR (300 MHz, CD_2Cl_2) δ 9.09 (d, *J* = 1.2 Hz, 1H, CH_{PM}), 7.95 (d, *J* = 15.9 Hz, 1H, $CH_{ethylenic}$), 7.94 (d, *J* = 15.9 Hz, 1H, $CH_{ethylenic}$), 7.73 (d, *J* = 9.5 Hz, 1H, $CH_{ethylenic}$), 7.67 (d, *J* = 8.4 Hz, 2H, $CH_{aromatic}$), 7.64 (d, *J* = 8.4 Hz, 2H, $CH_{aromatic}$), 7.62 (d, *J* = 8.4 Hz, 2H, $CH_{aromatic}$), 7.56 (d, *J* = 8.4 Hz, 2H, $CH_{aromatic}$), 7.52 – 7.48 (m, 2H, $CH_{aromatic}$), 7.45 (dd, *J* = 7.9, 1.5 Hz, 1H, $CH_{aromatic}$), 7.35 (d, *J* = 1.3 Hz, 1H, CH_{PM}), 7.17 (d, *J* = 15.9 Hz, 1H, $CH_{ethylenic}$), 7.14 (d, *J* = 15.9 Hz, 1H, $CH_{ethylenic}$), 6.76 (s, 2H, $CH_{aromatic}$), 6.41 (d, *J* = 9.6 Hz, 1H, $CH_{ethylenic}$), 3.99 (t, *J* = 13.7, 6.5 Hz, 4H, OCH_2), 3.96 (t, *J* = 13.7, 6.5 Hz, 2H, OCH_2), 1.82 (p, *J* = 6.6 Hz, 4H, CH_2), 1.72 (p, *J* = 6.8 Hz, 2H, CH_2), 1.51 – 1.44 (m, 6H, CH_2), 1.28 (broad s, 72H, CH_2), 0.89 (t, *J* = 7.0, 1.3 Hz, 6H, CH_3), 0.88 (t, *J* = 7.0 Hz, 3H, CH_3).

^{13}C NMR (75 MHz, $CDCl_3$) δ 162.70 (C_{quat}), 162.44 (C_{quat}), 160.28 (C_{quat}), 158.77 (CH), 153.87 (C_{quat}), 153.07 (C_{quat}), 142.67 (CH), 139.44 (C_{quat}), 136.38 (CH), 136.28 (C_{quat}), 136.03 (C_{quat}), 135.32 (C_{quat}), 132.30 (CH), 131.98 (CH), 127.76 (CH), 127.68 (CH), 127.65 (CH), 127.61 (CH), 126.95 (CH), 126.79 (C_{quat}), 126.33 (CH), 124.45 (C_{quat}), 123.25 (C_{quat}), 119.58 (CH), 118.82 (C_{quat}), 117.38 (C_{quat}), 117.02 (CH), 116.79 (CH), 110.31 (CH), 92.90 ($C_{quat\text{-}alkyne}$), 91.99 ($C_{quat\text{-}alkyne}$), 89.97 ($C_{quat\text{-}alkyne}$), 88.15 ($C_{quat\text{-}alkyne}$), 73.72 (OCH_2), 69.37 (OCH_2), 32.08 (CH_2), 30.49 (CH_2), 29.89 (CH_2), 29.86 (CH_2), 29.82 (CH_2), 29.80 (CH_2), 29.75 (CH_2), 29.56 (CH_2), 29.51 (CH_2), 26.25 (CH_2), 22.83 (CH_2), 14.24 (CH_3).

Anal. calcd for [$C_{87}H_{120}N_2O_5$]: C, 82.03; H, 9.50; N, 2.20 found: C, 81.91; H, 9.37; N, 2.01.

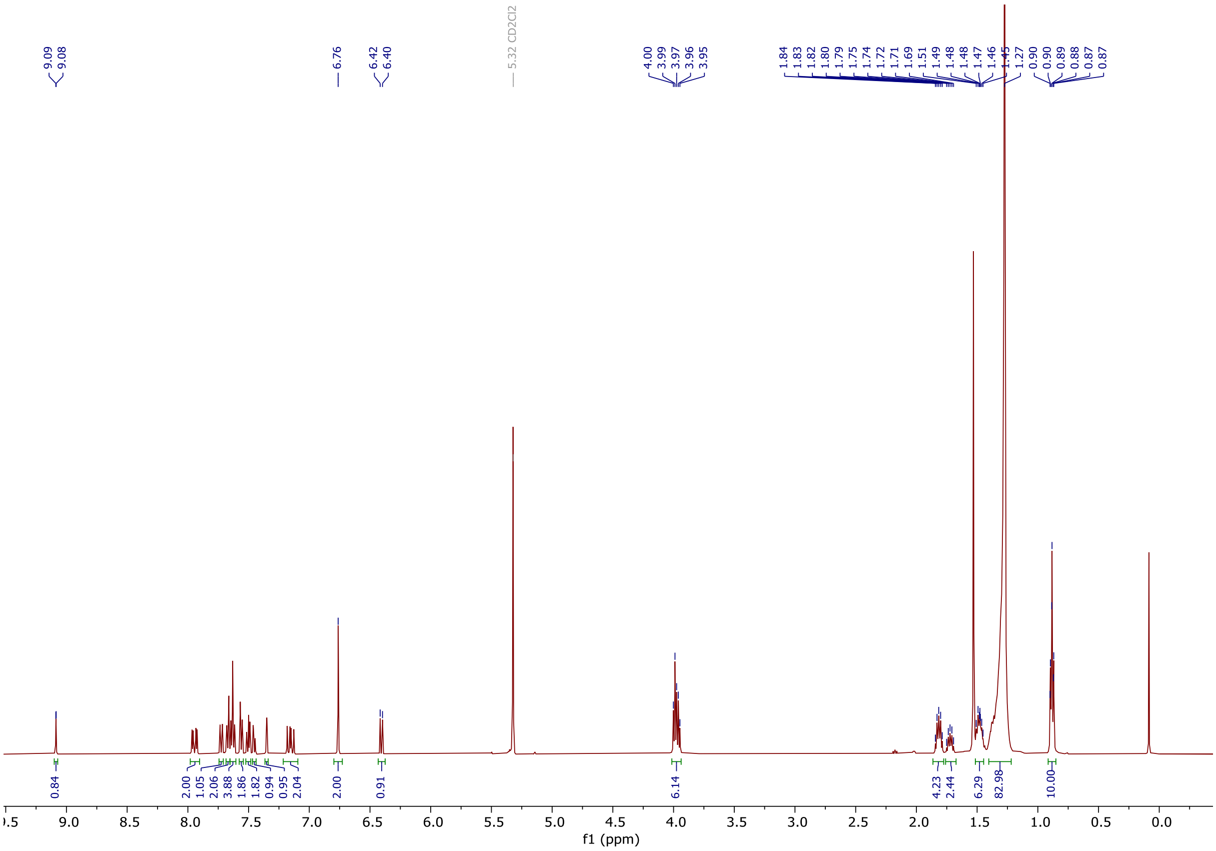

^{1}H NMR (300 MHz, CD_2Cl_2) spectrum of compound **PMC-3,4,5-C16**.

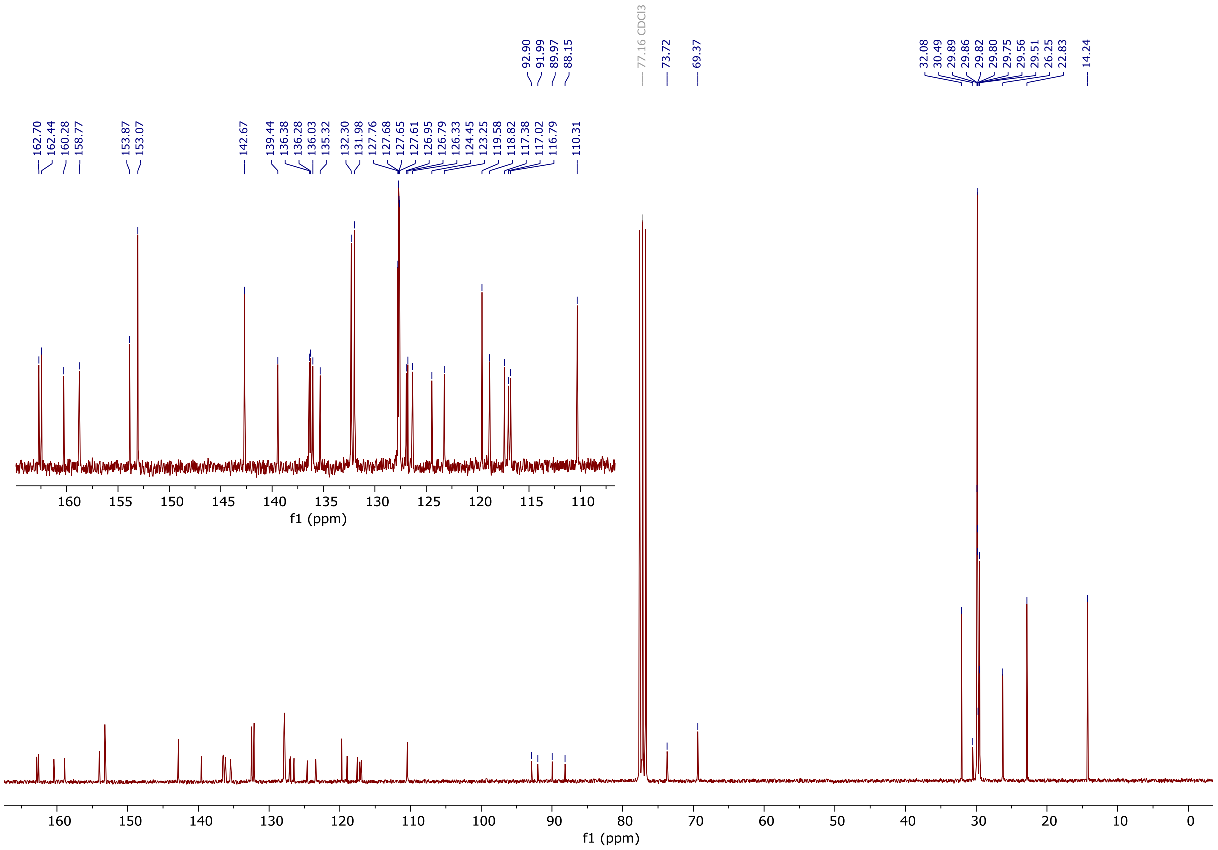

^{13}C NMR (75 MHz, $CDCl_3$) spectrum of compound **PMC-3,4,5-C16**.

Synthesis of compound 13 (3,4-C16):

Compound **13** was prepared from 4-ethynyl-1,2-bis(hexadecyloxy)benzene **42** and 7-((4-((E)-2-(6-((E)-4-bromostyryl)pyrimidin-4-yl)vinyl)phenyl)ethynyl)-2H-chromen-2-one **2**. The product was purified by silica gel chromatography (DCM – DCM: AcOEt (95:5) gradient in 30 minutes) and recrystallized by slow evaporation (CH_2Cl_2/MeOH) to give compound **13** (14 %).

^{1}H NMR (300 MHz, $CDCl_3$) δ 9.13 (d, *J* = 1.2 Hz, 1H, CH_{PM}), 7.92 (dd, *J* = 15.9, 3.2 Hz, 2H, $CH_{ethylenic}$), 7.69 (dd, *J* = 9.5, 0.7 Hz, 1H, $CH_{ethylenic}$), 7.66 – 7.39 (m, 11H, $CH_{aromatic}$), 7.30 (d, *J* = 1.3 Hz, 1H, CH_{PM}), 7.18 – 7.02 (m, 2H, $CH_{ethylenic}$, $CH_{aromatic}$), 6.84 (d, *J* = 8.3 Hz, 1H, $CH_{aromatic}$), 6.44 (d, *J* = 9.6 Hz, 1H, $CH_{ethylenic}$), 4.02 (t, *J* = 6.6 Hz, 4H, OCH_2), 1.91 – 1.75 (m, 4H, CH_2), 1.51 – 1.41 (s, 4H, CH_2), 1.26 (broad s, 48H, CH_2), 0.93 – 0.83 (m, 6H, CH_3).

^{13}C NMR (75 MHz, $CDCl_3$) δ 162.92 (C_{quat}), 162.60 (C_{quat}), 160.47 (C_{quat}), 158.95 (CH), 154.04 (C_{quat}), 150.18 (C_{quat}), 149.01 (C_{quat}), 142.85 (CH), 136.65 (CH), 136.46 (C_{quat}), 136.21 (CH), 135.30 (C_{quat}), 132.47 (CH), 132.07 (CH), 127.93 (CH), 127.85 (CH), 127.84 (CH), 127.77 (CH), 127.14 (CH), 126.97 (C_{quat}), 126.37 (CH), 125.29 (CH), 124.89 (C_{quat}), 123.41 (C_{quat}), 119.76 (CH), 119.00 (C_{quat}), 117.18 (CH) 116.98 (CH), 116.91 (CH), 115.27 (C_{quat}), 113.54 (CH), 92.91 ($C_{quat\text{-}alkyne}$), 92.00 ($C_{quat\text{-}alkyne}$), 89.97 (2 $C_{quat\text{-}alkyne}$), 69.52 (OCH2), 69.38 (OCH2), 32.09 (CH_2), 29.87 (CH_2), 29.82 (CH_2), 29.80 (CH_2), 29.79 (CH_2), 29.58 (CH_2), 29.52 (CH_2), 29.42 (CH_2), 29.38 (CH_2), 26.20 (CH_2), 26.17 (CH_2), 22.85 (CH_2), 14.26 (CH_3).

Anal. calcd for [$C_{71}H_{88}N_2O_4$.MeOH]: C, 81.16; H, 8.70; N, 2.63 found: C, 81.21; H, 8.15; N, 2.35.

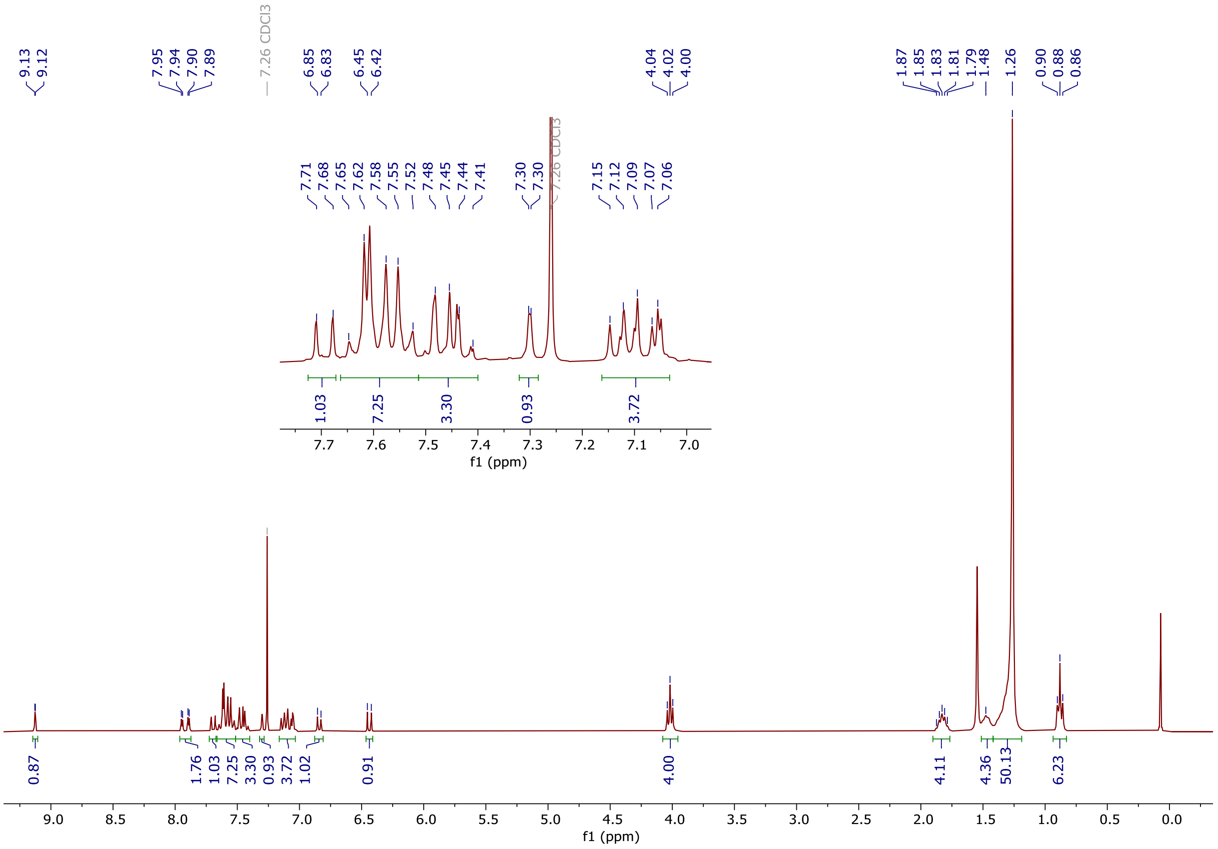

^{1}H NMR (300 MHz, $CDCl_3$) spectrum of compound **13**.

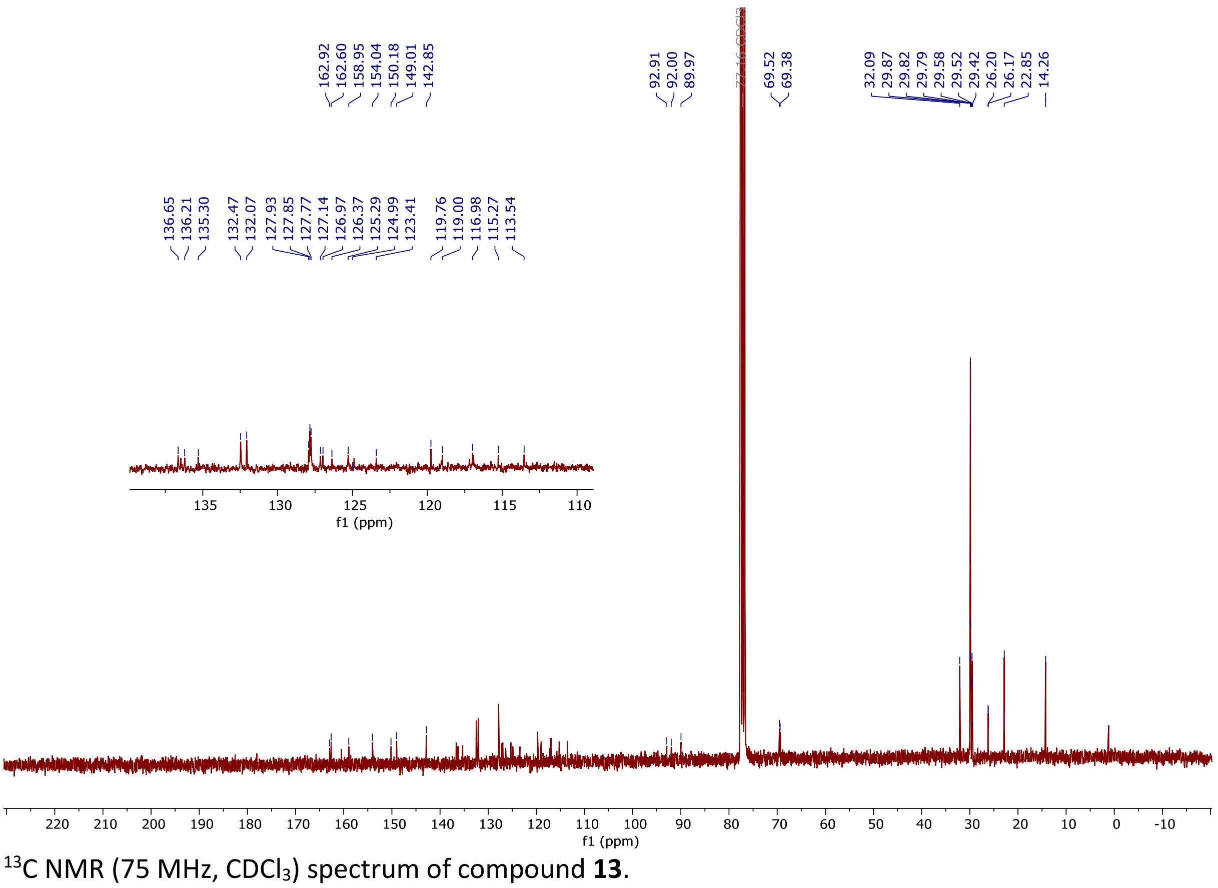

^{13}C NMR (75 MHz, $CDCl_3$) spectrum of compound **13**.

Synthesis of compound 3 ((S)-3,4,5-citro-C8(2)):

Compound ***3*** was prepared from 1,2,3-tris(((S)-3,7-dimethyloctyl)oxy)-5-ethynylbenzene **43** and 7-((4-((E)-2-(6-((E)-4-bromostyryl)pyrimidin-4-yl)vinyl)phenyl)ethynyl)-2H-chromen-2-one **2**. The product was purified by silica gel chromatography (DCM – DCM: AcOEt (95:5) gradient in 30 minutes) and recrystallized by slow evaporation (CH_2Cl_2/MeOH) to give compound **3** (43 %).

^{1}H NMR (300 MHz, $CDCl_3$) δ 9.12 (d, *J* = 1.2 Hz, 1H, CH_{PM}), 7.91 (d, *J* = 16.0 Hz, 1H, $CH_{aromatic}$), 7.90 (d, *J* = 16.0 Hz, 1H, $Ch_{aromatic}$), 7.67 (d, *J* = 9.5 Hz, 1H), 7.64 – 7.38 (m, 11H, $CH_{aromatic}$), 7.28 (d, *J* = 1.3 Hz, 1H, CH_{PM}), 7.1 (d, *J* = 15.9 Hz, 1H, $CH_{aromatic}$), 7.08 (d, *J* = 15.9 Hz, 1H, $CH_{aromatic}$), 6.76 (s, 2H, $CH_{aromatic}$), 6.42 (d, *J* = 9.5 Hz, 1H, $CH_{aromatic}$), 4.08 – 3.93 (m, 6H, OCH_2), 1.94 – 1.77 (m, 3H, C*H), 1.76 – 1.45 (m, 9H, CH, CH_2), 1.27 (broad s, 18H, CH_2), 0.94 (d, *J* = 6.4 Hz, 9H, C*CH_3), 0.87 (d, *J* = 6.6 Hz, 18H, CH_3).

^{13}C NMR (75 MHz, $CDCl_3$) δ 162.88 (C_{quat}), 162.63 (C_{quat}), 160.46 (C_{quat}), 158.95 (CH), 154.04 (C_{quat}), 153.25 (C_{quat}), 142.84 (CH), 139.58 (C_{quat}), 136.57 (CH), 136.45 (C_{quat}), 136.23 (CH), 135.51 (C_{quat}), 132.47 (CH), 132.15 (CH), 128.21 (CH), 127.93 (CH), 127.85 (CH), 127.82 (CH), 127.78 (CH), 127.13 (CH), 126.96 (C_{quat}), 126.51 (CH), 124.61 (C_{quat}), 123.43 (C_{quat}), 119.76 (CH), 119.00 (C_{quat}), 117.55 (C_{quat}), 117.20 (CH), 116.93 (CH), 110.41 (CH), 92.90 ($C_{quat-alkyne}$), 91.98 ($C_{quat-alkyne}$), 89.98 ($C_{quat-alkyne}$), 88.18 ($C_{quat-alkyne}$), 71.97 (OCH_2), 67.70 (2 OCH_2), 39.54 (CH_2), 39.45 (2 CH_2), 37.69 (CH_2), 37.51 (2 CH_2), 36.53 (3 CH_2), 30.03 (2 C*H), 29.86 (C*H), 28.15 (3 CH), 24.89(C*CH_2), 22.86 (2 C*CH), 22.77 (3 CH_3), 22.75 (3 C*CH_3), 19.75 (3 CH_3).

Anal. calcd for [$C_{69}H_{84}N_2O_5.CH_2Cl_2$]: C, 75.99; H, 7.84; N, 2.53 found: C, 75.48; H, 7.03; N, 2.43.

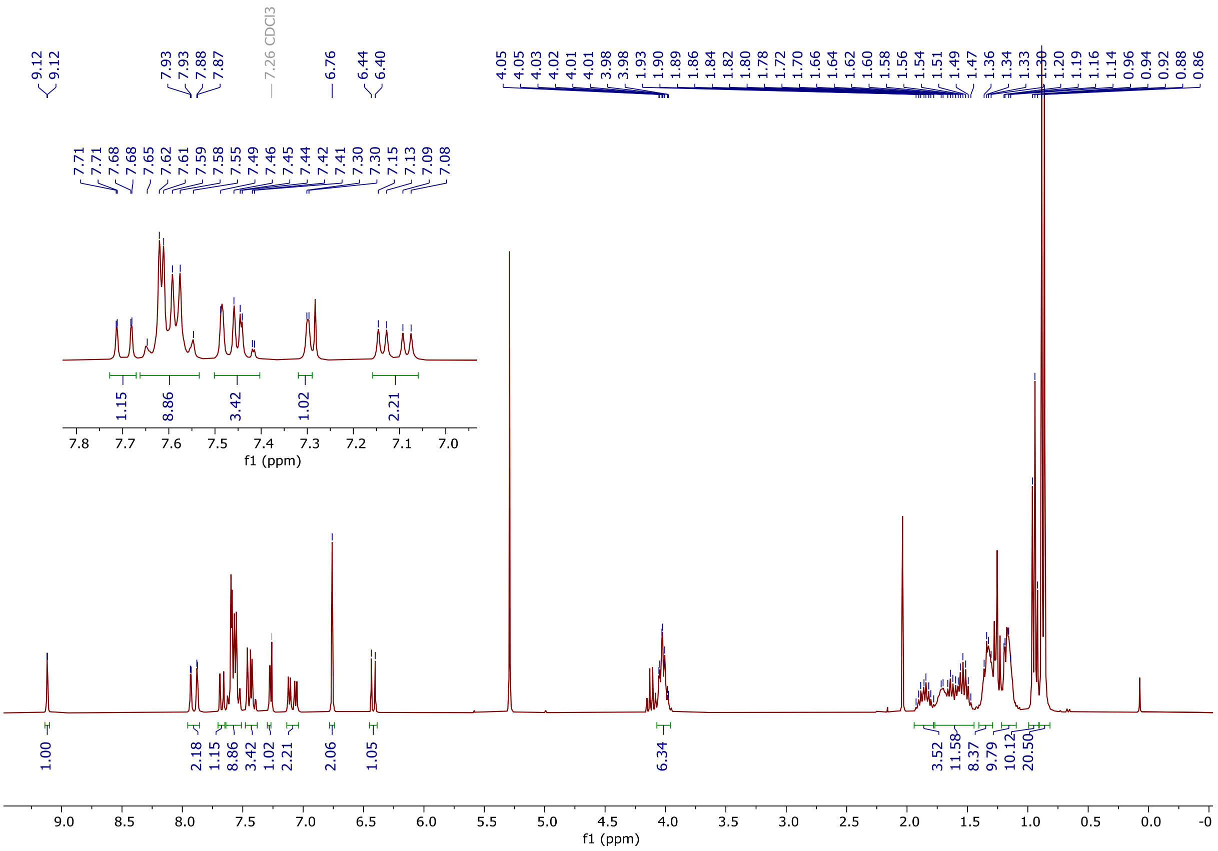

^{1}H NMR (300 MHz, $CDCl_3$) spectrum of compound **3**.

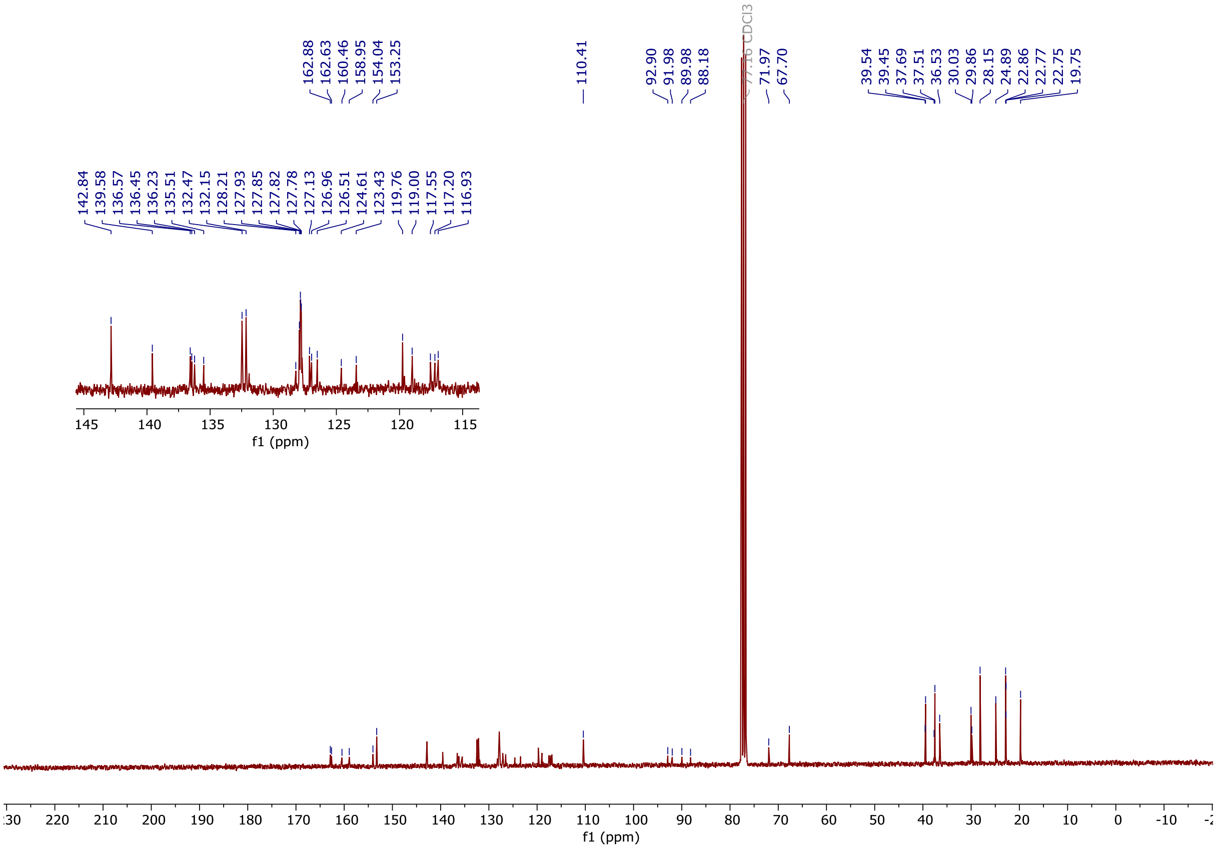

^{13}C NMR (75 MHz, $CDCl_3$) spectrum of compound **3**.

Synthesis of compound 4 ((S)- 3,4-citro-C8(2)):

Compound ***4*** was prepared from 1,2-bis(((S)-3,7-dimethyloctyl)oxy)-4-ethynylbenzene **44** and 7-((4-((E)-2-(6-((E)-4-bromostyryl)pyrimidin-4-yl)vinyl)phenyl)ethynyl)-2H-chromen-2-one **2**. The product was purified by silica gel chromatography (DCM – DCM: AcOEt (95:5) gradient in 30 minutes) and recrystallized by slow evaporation (CH_2Cl_2/MeOH) to give compound **4** (60 %).

^{1}H NMR (300 MHz, $CDCl_3$) δ 9.12 (d, *J* = 1.2 Hz, 1H, CH_{PM}), 7.91 (d, *J* = 15.9 Hz, 1H, $CH_{ethylenic}$), 7.90 (d, *J* = 15.9 Hz, 1H, $CH_{ethylenic}$), 7.67 (d, *J* = 9.5 Hz, 1H, $CH_{aromatic}$), 7.64 – 7.36 (m, 12H, $CH_{aromatic}$), 7.30 (d, *J* = 1.3 Hz, 1H, CH_{PM}), 7.16 – 7.00 (m, 4H, $CH_{ethylenic}$, $CH_{aromatic}$), 6.84 (d, *J* = 8.3 Hz, 1H, $CH_{aromatic}$), 6.42 (d, *J* = 9.5 Hz, 1H, $CH_{aromatic}$), 4.10 – 3.99 (m, 4H, OCH_2), 1.94 – 1.81 (m, 2H, CH), 1.78 – 1.59 (m, 2H, C*H), 1.58 – 1.45 (m, 8H, CH_2), 1.27 (broad s, 12H, CH_2), 0.96 (d, *J* = 6.4 Hz, 3H, C*CH_3), 0.95 (d, *J* = 6.4 Hz, 3H, C*CH_3), 0.87 (d, *J* = 6.6 Hz, 12H, CH_3).

^{13}C NMR (75 MHz, $CDCl_3$) δ 162.93 (C_{quat}), 162.61 (C_{quat}), 160.47 (C_{quat}), 158.96 (CH), 154.05 (C_{quat}), 150.15 (C_{quat}), 149.01 (C_{quat}), 142.86 (CH), 136.47 (CH), 136.21 (CH), 135.31 (C_{quat}), 133.06 (C_{quat}), 132.47 (CH), 132.07 (CH), 127.93 (CH), 127.85 (CH), 127.83 (CH), 127.77 (CH), 127.16 (CH), 126.96 (C_{quat}), 126.39 (CH), 125.26 (CH), 124.88 (C_{quat}), 123.42 (C_{quat}), 119.77 (CH), 119.00 (C_{quat}), 117.19 (CH), 116.92 (CH), 116.80 (CH), 115.23 (C_{quat}), 113.40 (CH), 92.91 (2 $C_{quat\text{-}alkyne}$), 92.00 ($C_{quat\text{-}alkyne}$), 87.90 ($C_{quat\text{-}alkyne}$), 67.84 (OCH_2), 67.72 (OCH_2), 39.43 (2CH_2) , 37.51 (2 CH_2), 36.37 (CH_2), 36.31 (CH_2), 30.13 (2 C*H), 28.15 (2 CH), 24.88 (2 C*CH_2), 22.85 (2 CH_3), 22.76 (2 C*CH_3), 19.87 (2 CH_3).

Anal. calcd for [$C_{59}H_{64}N_2O_4$]: C, 81.91; H, 7.46; N, 3.24 found: C, 81.68; H, 7.29; N, 3.01.

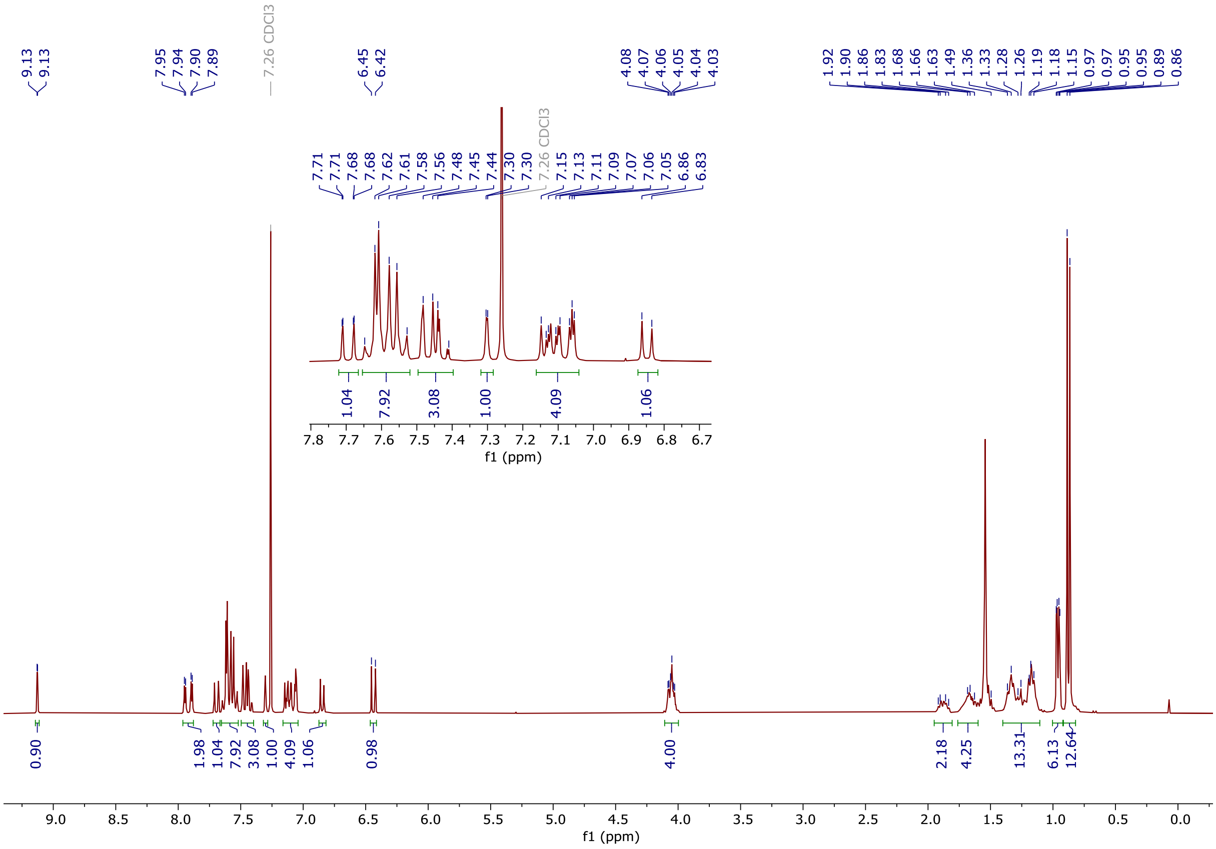

^{1}H NMR (300 MHz, $CDCl_3$) spectrum of compound **4**.

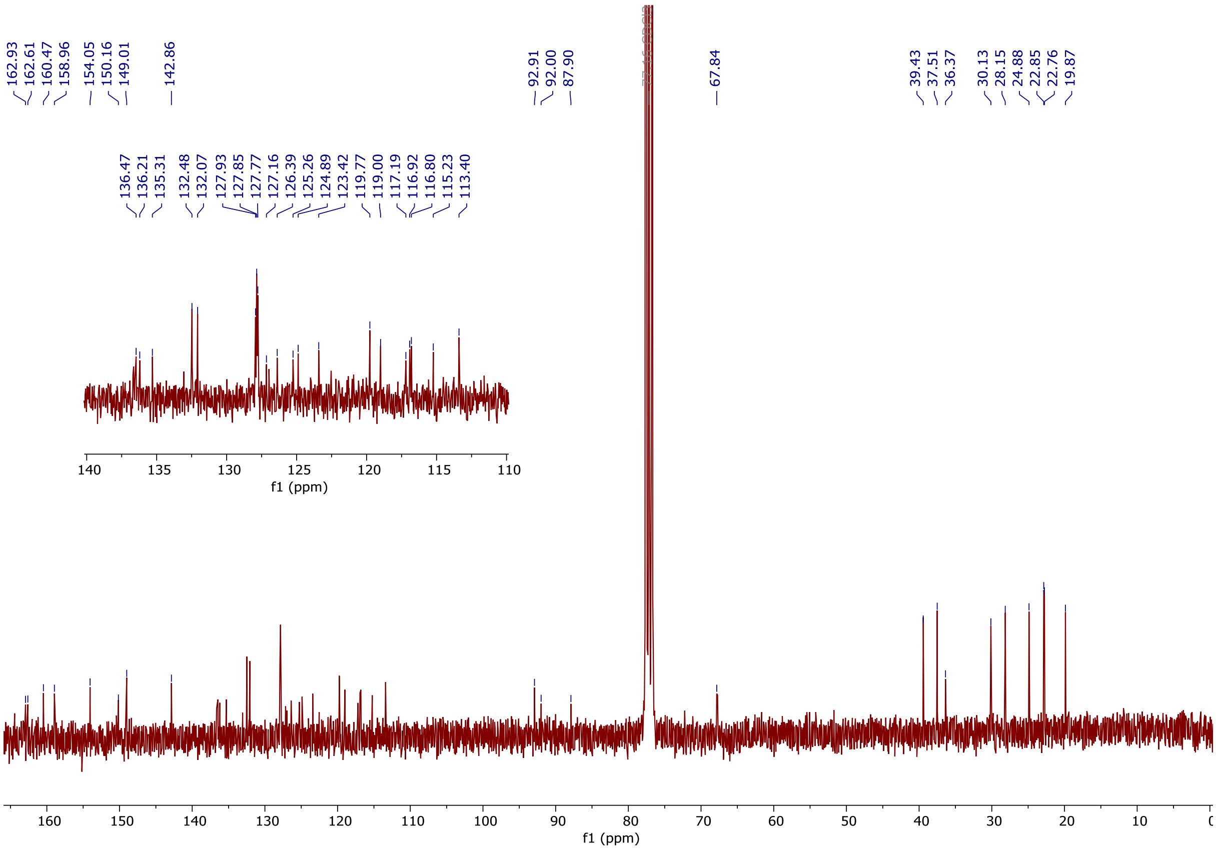

^{13}C NMR (75 MHz, $CDCl_3$) spectrum of compound **4**.

Synthesis of compound 5 ((S)-4-citro-C8(2)):

Compound ***5*** was prepared from (S)-1-((3,7-dimethyloctyl)oxy)-4-ethynylbenzene **45** and 7-((4-((E)-2-(6-((E)-4-bromostyryl)pyrimidin-4-yl)vinyl)phenyl)ethynyl)-2H-chromen-2-one **2**. The product was purified by silica gel chromatography (DCM – DCM: AcOEt (95:5) gradient in 30 minutes) and recrystallized by slow evaporation (CH_2Cl_2/MeOH) to give compound **5** (32 %).

^{1}H NMR (300 MHz, $CDCl_3$) δ 9.13 (s, 1H, CH_{PM}), 7.93 (d, *J* = 15.9 Hz, 1H, $CH_{ethylenic}$), 7.91 (d, *J* = 15.9 Hz, 1H, $CH_{ethylenic}$), 7.70 (d, *J* = 9.5 Hz, 1H, $CH_{aromatic}$), 7.66 – 7.40 (m, 13H, $CH_{aromatic}$), 7.31 (s, 1H, CH_{PM}), 7.12 (d, *J* = 15.9 Hz, 1H, $CH_{ethylenic}$), 7.10 (d, *J* = 15.9 Hz, 1H, $CH_{ethylenic}$), 6.91 – 6.85 (m, 2H, $CH_{aromatic}$), 6.44 (d, *J* = 9.5 Hz, 1H, $CH_{aromatic}$), 4.08 – 3.95 (m, 2H, OCH_2), 1.90 – 1.77 (m, 1H, C*H), 1.67 – 1.47 (m, 3H, CH, CH_2), 1.36 – 1.12 (m, 6H, CH_2), 0.95 (d, *J* = 6.4 Hz, 3H, C*CH_3), 0.87 (d, *J* = 6.9 Hz, 6H, CH_3).

^{13}C NMR**:** not enough soluble

Anal. calcd for [$C_{49}H_{44}N_2O_3.CH_2Cl_2$]: C, 75.65; H, 5.84; N, 3.53 found: C, 76.44; H, 6.61; N, 2.97.

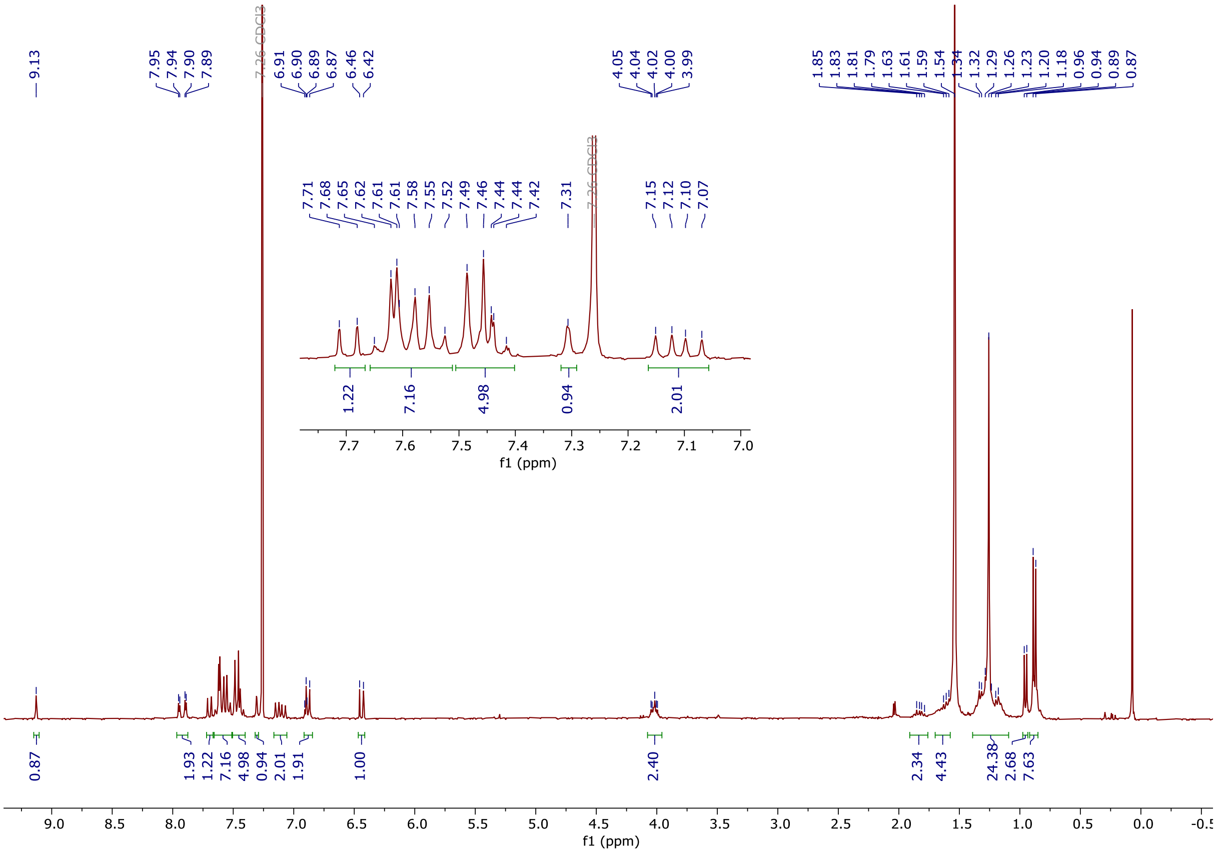

^{1}H NMR (300 MHz, $CDCl_3$) spectrum of compound **5**.

Synthesis of compound 6 ((R)-3,4-lact-C8):

Compound ***6*** was prepared from 4-ethynyl-1,2-bis((R)-2-(octyloxy)propoxy)benzene **46** and 7-((4-((E)-2-(6-((E)-4-bromostyryl)pyrimidin-4-yl)vinyl)phenyl)ethynyl)-2H-chromen-2-one **2**. The product was purified by silica gel chromatography (DCM – DCM: AcOEt (95:5) gradient in 30 minutes) and recrystallized by slow evaporation (CH_2Cl_2/MeOH) to give compound **6** (66 %).

^{1}H NMR (300 MHz, $CDCl_3$) δ 9.12 (d, *J* = 1.2 Hz, 1H, CH_{PM}), 7.91 (dd, *J* = 15.9, 2.5 Hz, 2H, $CH_{aromatic}$), 7.70 (d, *J* = 9.5 Hz, 1H, $CH_{ethylenic}$), 7.69 (d, *J* = 9.5 Hz, 1H, $CH_{ethylenic}$), 7.65 – 7.39 (m, 11H, $CH_{aromatic}$), 7.29 (d, *J* = 1.3 Hz, 1H, CH_{PM}), 7.15 – 7.04 (m, 4H, 2 $CH_{ethylenic}$, 2 $CH_{aromatic}$), 6.89 – 6.82 (m, 1H, $CH_{aromatic}$), 6.43 (d, *J* = 9.5 Hz, 1H, $CH_{aromatic}$), 4.05 (m, 2H, C*CH_2), 3.93 – 3.76 (m, 4H, C*CH_2 and C*H), 3.64 – 3.51 (m, 4H, OCH_2), 1.59 – 1.50 (m, 4H, CH_2), 1.28 (broad s, 20H, CH_2), 0.89 (t, *J* = 4.4 Hz, 3H, CH_3), 0.88 (t, *J* = 4.4 Hz, 3H, CH_3).

^{13}C NMR (75 MHz, $CDCl_3$) δ 162.88 (C_{quat}), 162.57 (C_{quat}), 160.50 (C_{quat}), 158.91 (CH), 153.99 (C_{quat}), 149.90 (C_{quat}), 148.79 (C_{quat}), 142.88 (CH), 136.60 (CH), 136.41 (C_{quat}), 136.17 (CH), 135.30 (C_{quat}), 132.46 (CH), 132.05 (CH), 127.93 (CH), 127.82 (CH), 127.76 (CH), 127.09 (CH), 126.93 (C_{quat}), 126.35 (CH), 125.50 (CH), 124.76 (C_{quat}), 123.38 (C_{quat}), 119.73 (CH), 118.97 (C_{quat}), 117.16 (CH), 117.07 (CH), 116.97 (CH), 115.63 (C_{quat}), 113.75 (CH), 113.63 (CH), 92.90 ($C_{quat\text{-}alkyne}$), 91.76 ($C_{quat\text{-}alkyne}$), 89.96 ($C_{quat\text{-}alkyne}$), 88.04 ($C_{quat\text{-}alkyne}$), 73.99 (C*H), 73.96 (C*H), 73.14 (OCH2), 73.00 (OCH2), 70.01 (OCH2), 69.94 (OCH2), 32.00 (CH2), 30.33 (CH2), 29.63 (CH2), 29.45 (CH2), 26.32 (CH2), 22.81 (CH2), 17.73 (C*CH3), 17.69 (C*CH3), 14.25 (CH3).

Anal. calcd for [$C_{61}H_{68}N_2O_6$]: C, 79.19; H, 7.41; N, 3.03 found: C, 79.20; H, 7.15; N, 2.97.

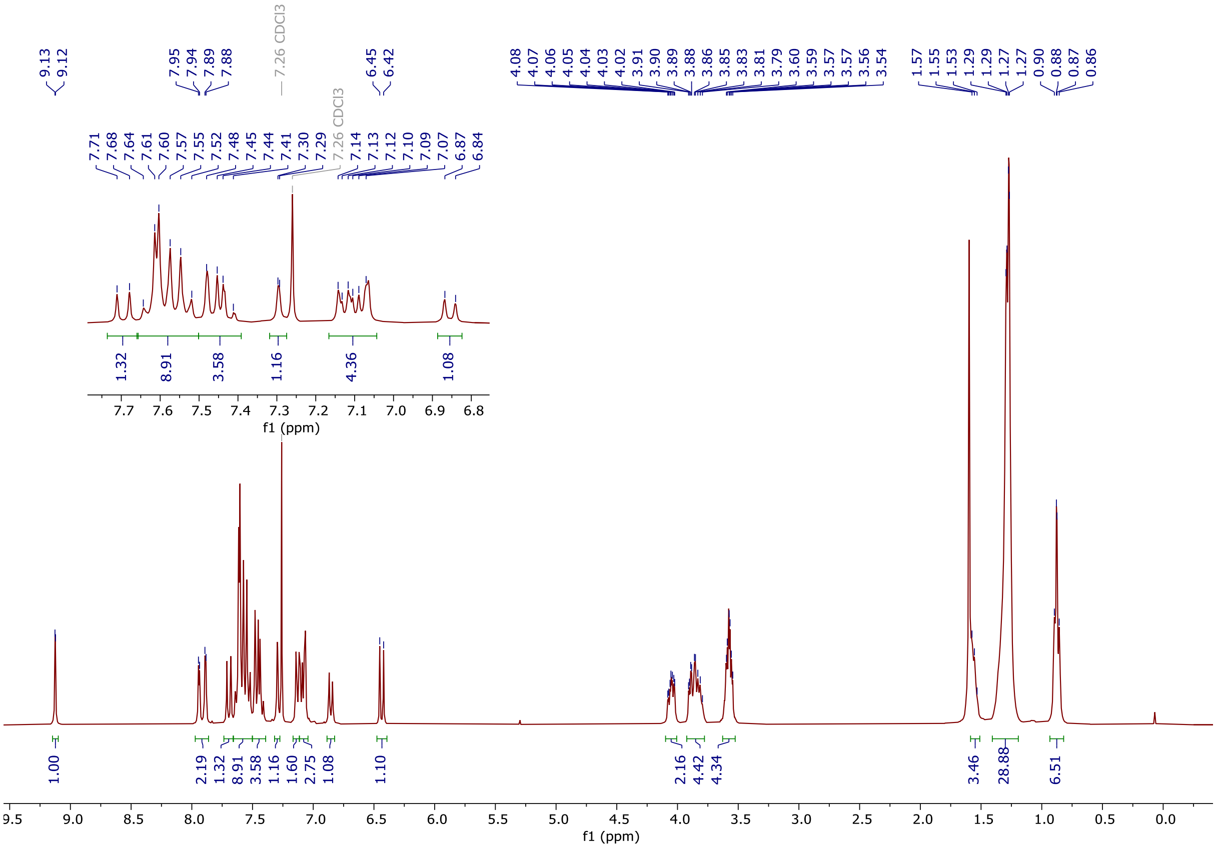

^{1}H NMR (300 MHz, $CDCl_3$) spectrum of compound **6**.

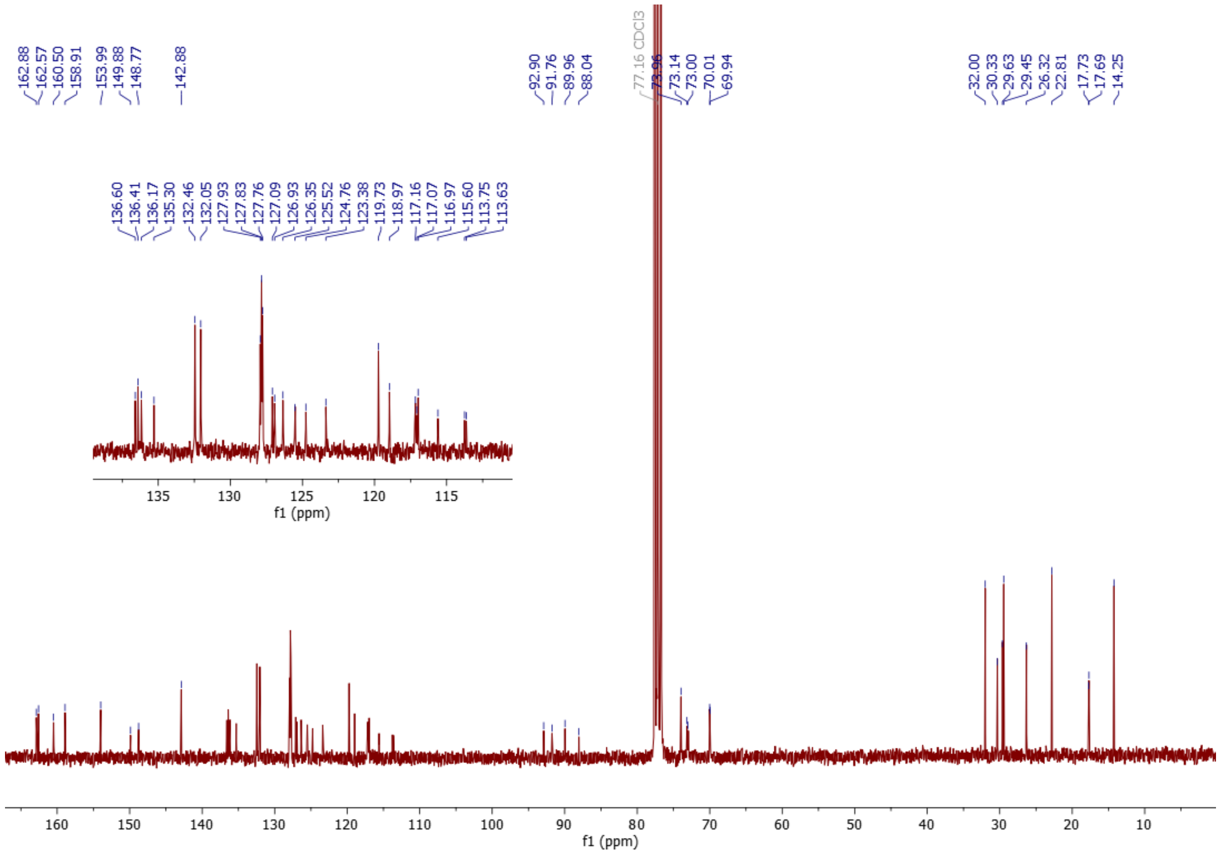

^{13}C NMR (75 MHz, $CDCl_3$) spectrum of compound **6**.

Synthesis of compound 7 ((R)-4-lact-C8):

Compound ***7*** was prepared from (R)-1-ethynyl-4-(2-(octyloxy)propoxy)benzene **47** and 7-((4-((E)-2-(6-((E)-4-bromostyryl)pyrimidin-4-yl)vinyl)phenyl)ethynyl)-2H-chromen-2-one **2**. The product was purified by silica gel chromatography (DCM – DCM: AcOEt (95:5) gradient in 30 minutes) and recrystallized by slow evaporation (CH_2Cl_2/MeOH) to give compound **7** (48 %).

^{1}H NMR (300 MHz, $CDCl_3$) δ 9.13 (d, *J* = 1.2 Hz, 1H, CH_{PM}), 7.93 (d, *J* = 15.9 Hz, 1H, $CH_{ethylenic}$), 7.92 (d, *J* = 15.9 Hz, 1H, $CH_{ethylenic}$), 7.69 (dd, *J* = 9.6, 0.7 Hz, 1H, $CH_{aromatic}$), 7.66 – 7.40 (m, 13H, $CH_{aromatic}$), 7.30 (d, *J* = 1.3 Hz, 1H, CH_{PM}), 7.12 (d, *J* = 15.9 Hz, 1H, $CH_{ethylenic}$), 7.10 (d, *J* = 15.9 Hz, 1H, $CH_{ethylenic}$), 6.94 – 6.85 (m, 2H, $CH_{aromatic}$), 6.44 (d, *J* = 9.5 Hz, 1H, $CH_{aromatic}$), 4.01 (dd, *J* = 9.6, 5.8 Hz, 1H, C*CH_2), 3.89 (dd, *J* = 9.6, 4.8 Hz, 1H, C*CH_2), 3.84 – 3.74 (m, 1H, C*H), 3.63 – 3.45 (m, 2H, OCH_2), 1.66 – 1.40 (m, 4H, CH_2), 1.33 – 1.23 (m, 11H, CH_2, C*CH3), 0.90 (m, 3H, CH_3).

^{13}C NMR (75 MHz, $CDCl_3$) δ 162.92 (C_{quat}), 162.59 (C_{quat}), 160.50 (C_{quat}), 159.38 (C_{quat}), 158.94 (CH), 154.03 (C_{quat}), 142.88 (CH), 136.65 (CH), 136.44 (C_{quat}), 136.19 (CH), 135.27 (C_{quat}), 133.26 (CH), 132.47 (CH), 132.05 (CH), 127.94 (CH), 127.86 (CH), 127.84 (CH), 127.77 (CH), 127.13 (CH), 126.96 (C_{quat}), 126.35 (CH), 124.90 (C_{quat}), 123.40 (C_{quat}), 119.76 (CH), 118.99 (C_{quat}), 117.19 (CH), 116.95 (CH), 115.33 (C_{quat}), 114.90 (CH), 92.91 ($C_{quat\text{-}alkyne}$), 91.68 ($C_{quat\text{-}alkyne}$), 89.96 ($C_{quat\text{-}alkyne}$), 88.18 ($C_{quat\text{-}alkyne}$), 73.81 (C*H), 71.87 (OCH_2), 69.83 (OCH_2), 31.99 (CH_2), 30.25 (CH_2), 29.59 (CH_2), 29.44 (CH_2), 26.29 (CH_2), 22.82 (CH_2), 17.50 (C*CH_3), 14.25 (CH_3).

Anal. calcd for [$C_{50}H_{46}N_2O_4.CH_2Cl_2$]: C, 74.35; H, 5.87; N, 3.40 found: C, 74.25; H, 5.96; N, 2.86.

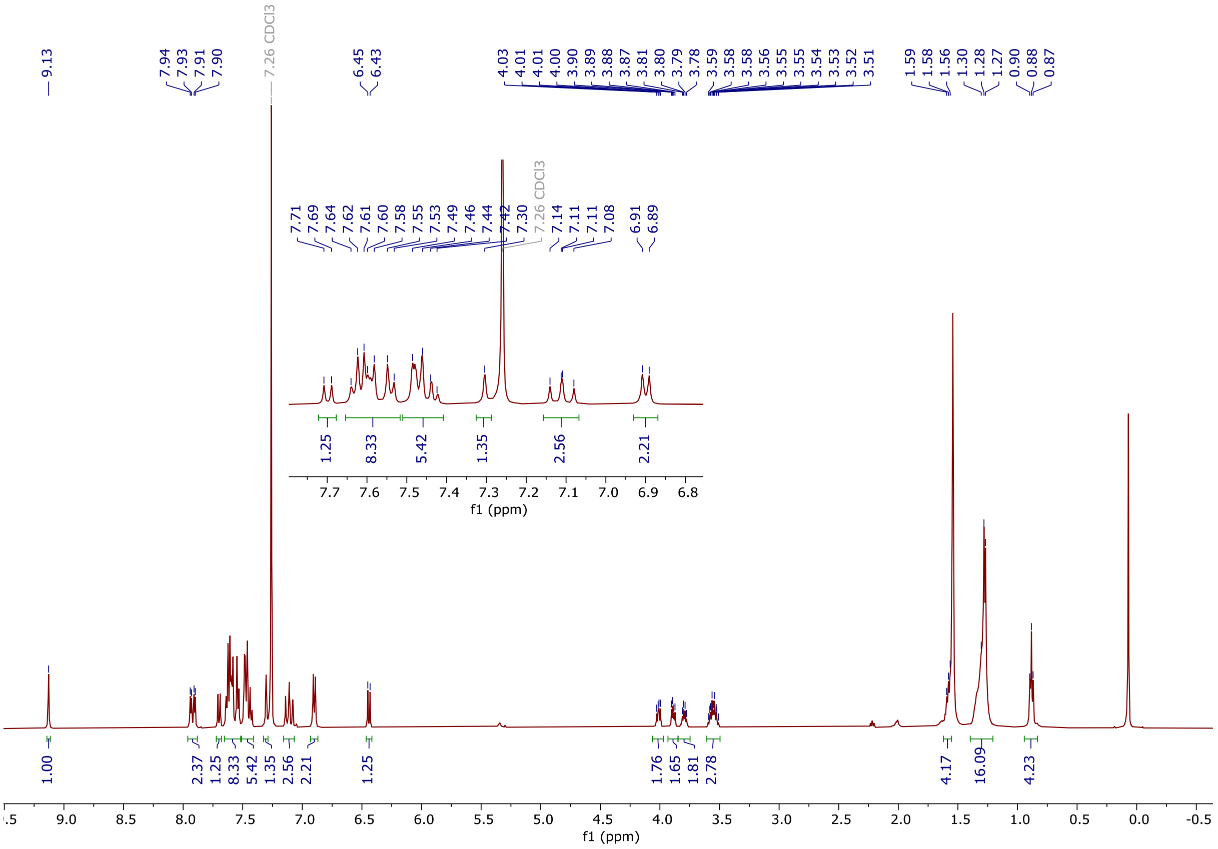

^{1}H NMR (300 MHz, $CDCl_3$) spectrum of compound **7**.

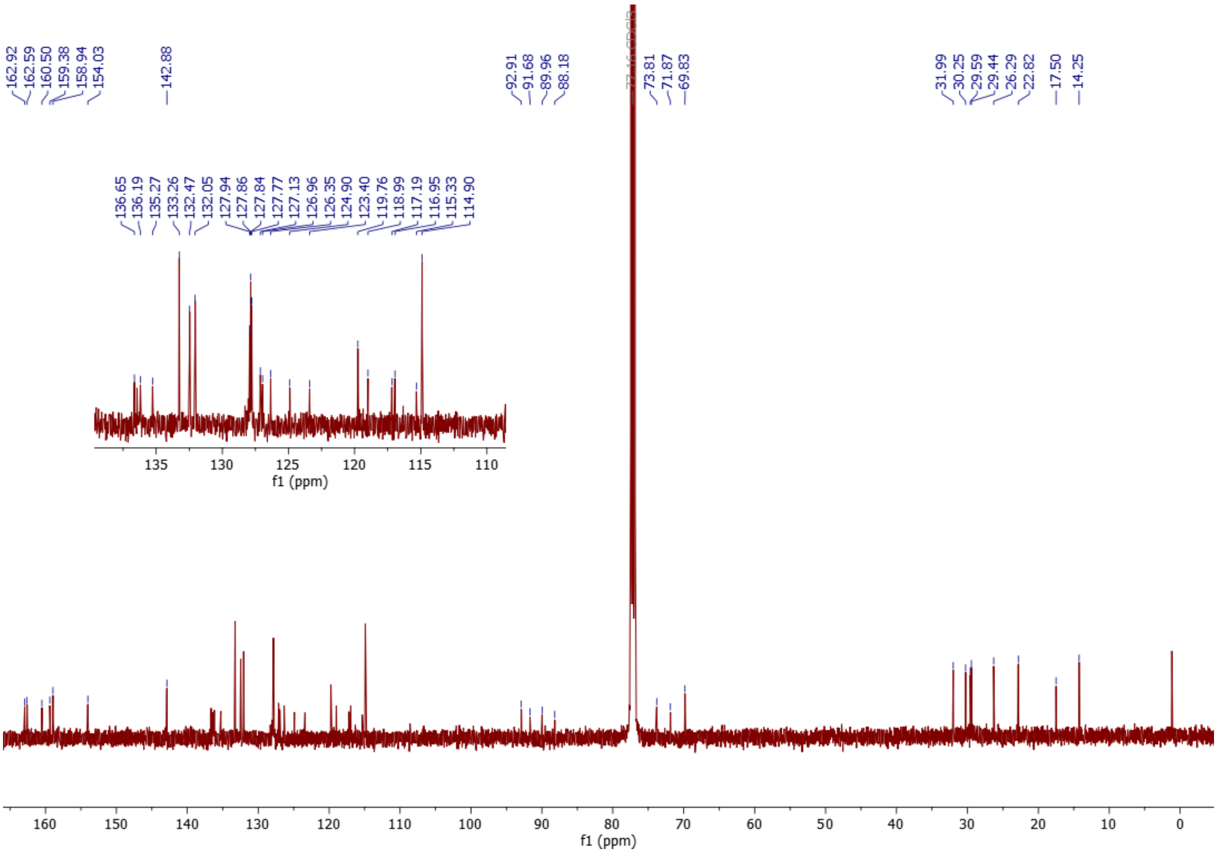

^{13}C NMR (75 MHz, $CDCl_3$) spectrum of compound **7**.

Synthesis of compound 8 ((S)-4-diol-C8):

Compound ***8*** was prepared from (S)-1-(2,3-bis(octyloxy)propoxy)-4-ethynylbenzene **48** and 7-((4-((E)-2-(6-((E)-4-bromostyryl)pyrimidin-4-yl)vinyl)phenyl)ethynyl)-2H-chromen-2-one **2**. The product was purified by silica gel chromatography (DCM – DCM: AcOEt (95:5) gradient in 30 minutes) and recrystallized by slow evaporation (CH_2Cl_2/MeOH) to give compound **8** (28 %).

^{1}H NMR (300 MHz, $CDCl_3$) δ 9.13 (s, 1H, CH_{PM}), 7.92 (d, *J* = 16.0 Hz, 1H, $CH_{ethylenic}$), 7.91 (d, *J* = 16.0 Hz, 1H, $CH_{ethylenic}$), 7.72 (d, J = 9.6 Hz, 1H, $CH_{aromatic}$), 7.68 – 7.42 (m, 13H, $CH_{aromatic}$), 7.31 (s, 1H, CH_{PM}), 7.11 (d, *J* = 16.0 Hz, 1H, $CH_{ethylenic}$), 7.10 (d, *J* = 16.0 Hz, 1H, $CH_{ethylenic}$), 6.94 – 6.88 (m, 2H, $CH_{aromatic}$), 6.44 (d, *J* = 9.5 Hz, 1H, $CH_{aromatic}$), 4.17 – 4.00 (m, 2H, C*CH_2), 3.82 – 3.75 (m, 1H, C*H), 3.66 – 3.56 (m, 4H, OCH_2), 3.47 (t, *J* = 6.6 Hz, 2H, OCH_2), 1.62 – 1.51 (m, 4H, CH_2), 1.27 (broad s, 20H, CH_2), 0.94 – 0.81 (m, 6H, CH_3).

^{13}C NMR (75 MHz, $CDCl_3$) δ 162.90 (C_{quat}), 162.57 (C_{quat}), 160.45 (C_{quat}), 159.38 (C_{quat}), 158.92 (CH), 154.02 (C_{quat}), 142.84 (CH), 136.63 (CH), 136.44 (C_{quat}), 136.16 (CH), 135.26 (C_{quat}), 133.23 (CH), 132.45 (CH), 132.04 (CH), 129.22 (CH), 127.92 (CH), 127.83 (CH), 127.82 (CH), 127.75 (CH), 127.12 (CH), 126.95 (C_{quat}), 126.34 (CH), 124.91 (C_{quat}), 123.38 (C_{quat}), 119.73 (CH), 118.97 (C_{quat}), 117.16 (CH), 116.91 (CH), 115.37 (C_{quat}), 114.94 (CH), 92.91 ($C_{quat-alkyne}$), 91.70 ($C_{quat-alkyne}$), 89.96 ($C_{quat-alkyne}$), 88.18 ($C_{quat-alkyne}$), 71.95 (OCH_2), 71.04 (OCH_2), 70.32 (OCH_2), 68.46 (OCH_2), 31.98 (CH_2), 30.19 (CH_2), 29.78 (CH_2), 29.57 (CH_2), 29.42 (CH_2), 26.28 (CH_2), 26.20 (CH_2), 22.80 (CH_2), 14.23 (CH_3).

Anal. calcd for [$C_{58}H_{62}N_2O_5$]: C, 80.34; H, 7.21; N, 3.23 found: C, 79.42; H, 6.71; N, 3.33.

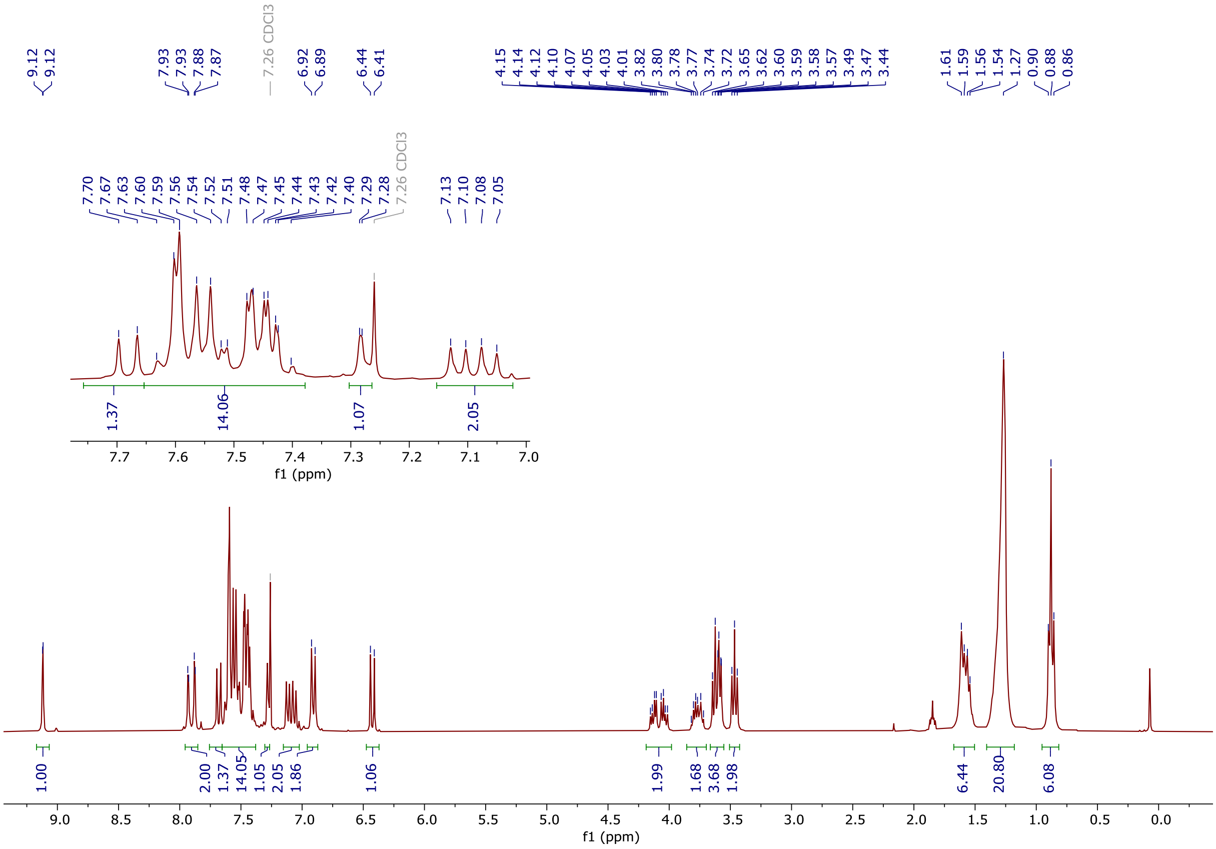

^{1}H NMR (300 MHz, $CDCl_3$) spectrum of compound **8**.

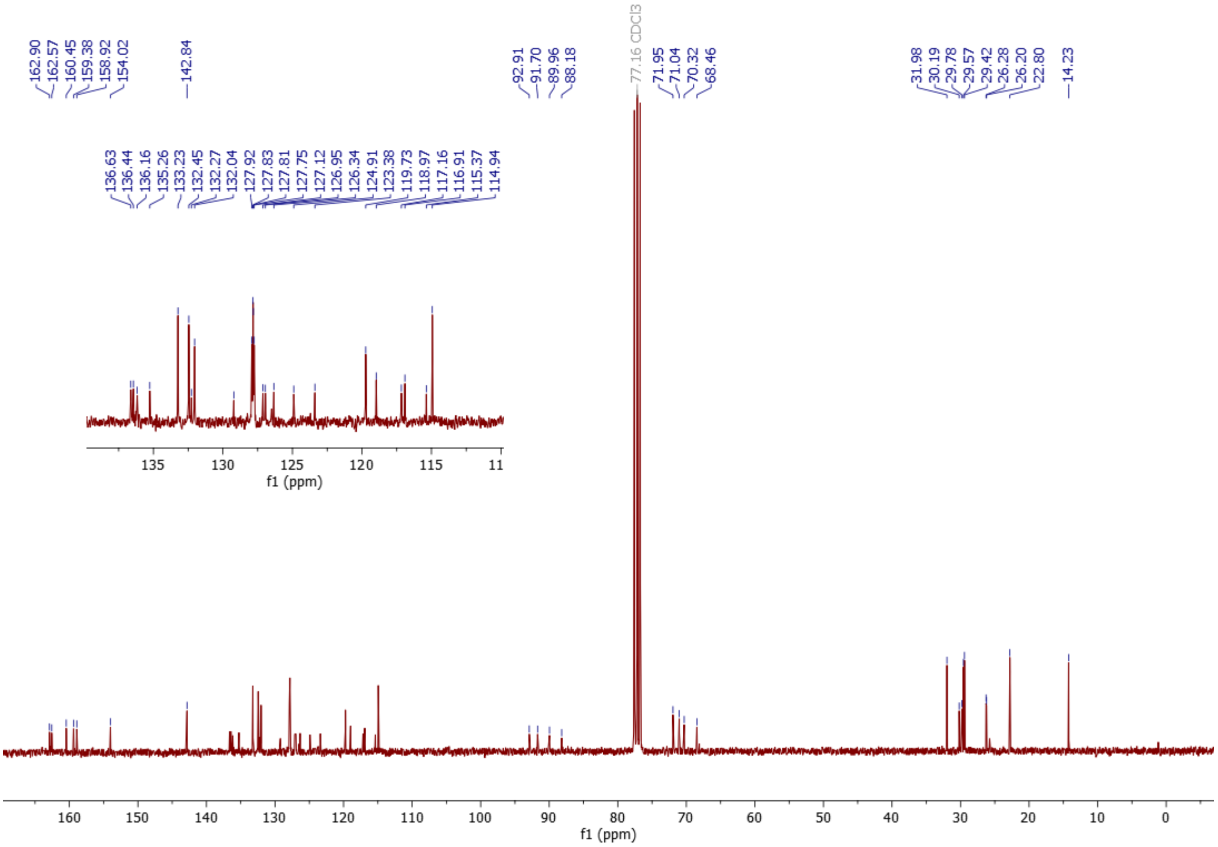

^{13}C NMR (75 MHz, $CDCl_3$) spectrum of compound **8**.

Synthesis of compound 9 ((S)-4-diol-C12:

Compound ***9*** was prepared from (S)-1-(2,3-bis(dodecyloxy)propoxy)-4-ethynylbenzene **49** and 7-((4-((E)-2-(6-((E)-4-bromostyryl)pyrimidin-4-yl)vinyl)phenyl)ethynyl)-2H-chromen-2-one **25**. The product was purified by silica gel chromatography (DCM – DCM: AcOEt (95:5) gradient in 30 minutes) and recrystallized by slow evaporation (CH_2Cl_2/MeOH) to give compound **9** (49 %).

^{1}H NMR (300 MHz, $CDCl_3$) δ 9.12 (d, *J* = 1.2 Hz, 1H, CH_{PM}), 7.91 (d, *J* = 15.9 Hz, 1H, $CH_{ethylenic}$), 7.90 (d, *J* = 15.9 Hz, 1H, $CH_{ethylenic}$), 7.68 (d, *J* = 9.5 Hz, 1H, $CH_{aromatic}$), 7.62J – 7.41 (m, 13H, $CH_{aromatic}$), 7.30 (d, *J* = 1.3 Hz, 1H, CH_{PM}), 7.12 (d, *J* = 15.9 Hz, 1H, $CH_{ethylenic}$), 7.09 (d, *J* = 15.9 Hz, 1H, $CH_{aromatic}$), 6.93 – 6.90 (m, 2H, $CH_{aromatic}$), 6.43 (d, *J* = 9.5 Hz, 1H, $CH_{aromatic}$), 4.16 – 4.02 (m, 2H, C*CH_2), 3.82 – 3.72 (m, 1H, C*H), 3.65 – 3.58 (m, 4H, OCH_2), 3.49 – 3.44 (m, 2H, OCH_2), 1.62 – 1.50 (m, 4H, CH_2), 1.26 (broad s, 36H, CH_2), 0.90 – 0.85 (m, 6H, CH_3).

^{13}C NMR (75 MHz, $CDCl_3$) δ 162.94 (C_{quat}), 162.61 (C_{quat}), 160.48 (C_{quat}), 159.40 (C_{quat}), 158.95 (CH), 154.04 (C_{quat}), 142.86 (CH), 136.68 (CH), 136.47 (C_{quat}), 136.21 (CH), 135.28 (C_{quat}), 133.25 (CH), 132.47 (CH), 132.06 (CH), 127.93 (CH), 127.85 (CH), 127.83 (CH), 127.77 (CH), 127.15 (CH), 126.98 (C_{quat}), 126.36 (CH), 124.94 (C_{quat}), 123.41 (C_{quat}), 119.76 (CH), 119.00 (C_{quat}), 117.20 (CH), 116.91 (CH), 115.39 (C_{quat}), 114.95 (CH), 92.92 (2 $C_{quat\text{-}alkyne}$), 91.70 ($C_{quat\text{-}alkyne}$), 89.97 ($C_{quat\text{-}alkyne}$), 71.96 (OCH_2), 71.05 (OCH_2), 70.33 (OCH_2), 68.48 (OCH_2), 32.08 (CH_2), 30.21 (CH_2), 29.85 (CH_2), 29.80 (CH_2), 29.63 (CH_2), 29.52 (CH_2), 26.29 (CH_2), 26.22 (CH_2), 22.84 (CH_2), 14.25 (CH_3).

Anal. calcd for [$C_{66}H_{78}N_2O_5$]: C, 80.94; H, 8.03; N, 2.86 found: C, 80.87; H, 7.79; N, 2.68.

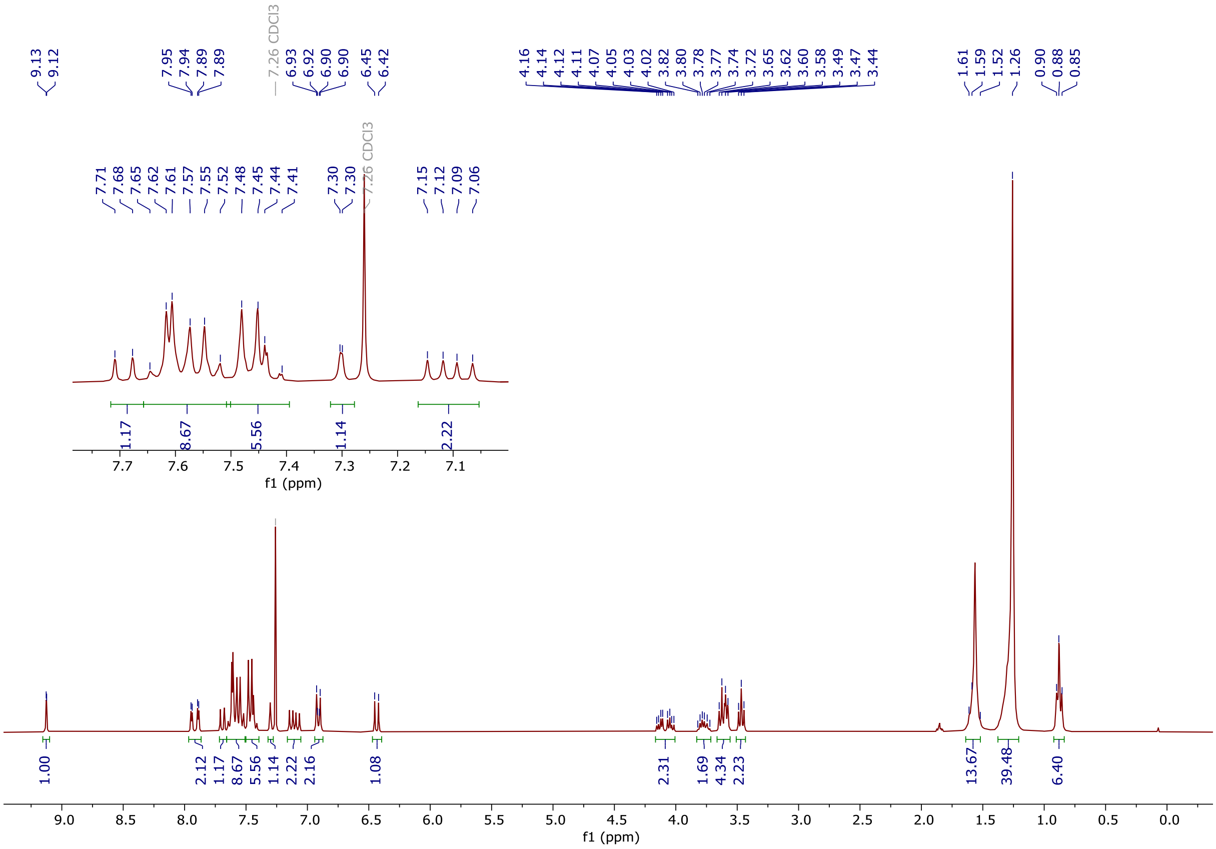

^{1}H NMR (300 MHz, $CDCl_3$) spectrum of compound **9**.

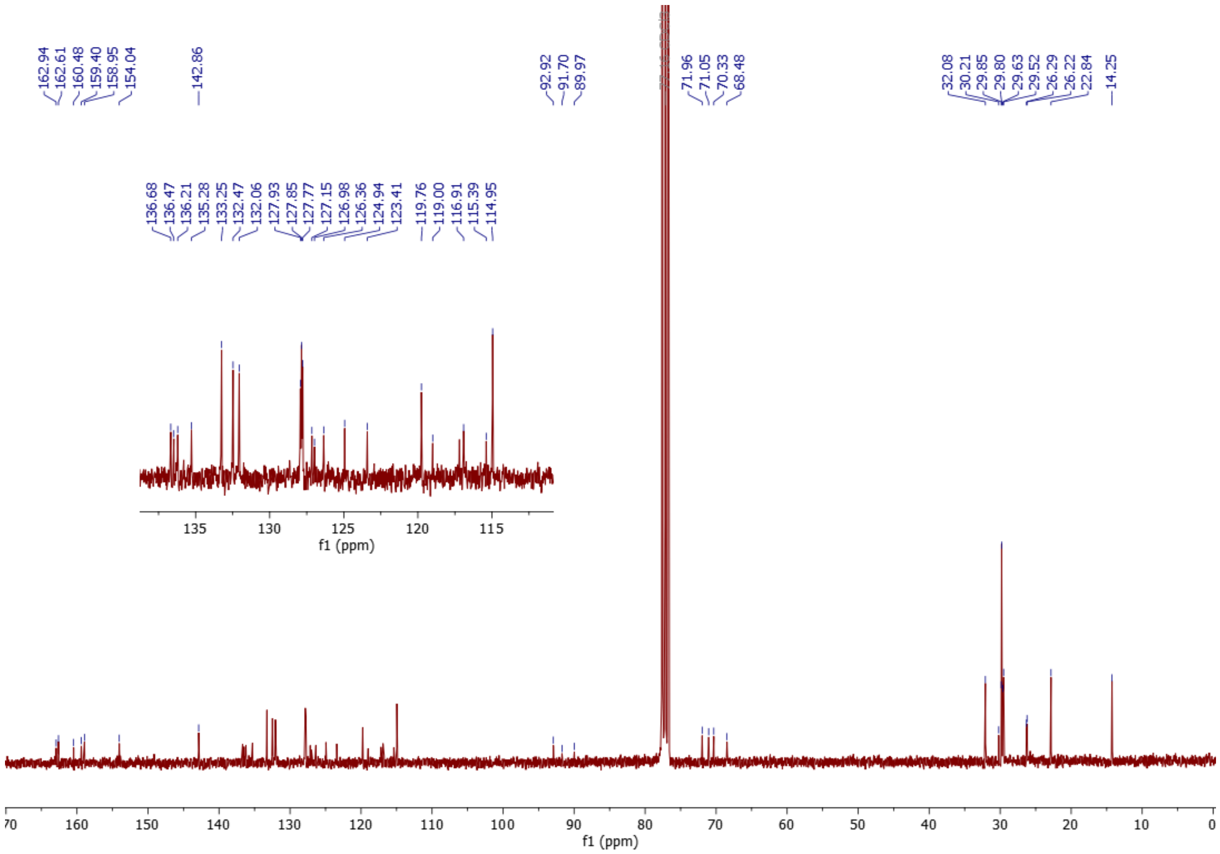

^{13}C NMR (75 MHz, $CDCl_3$) spectrum of compound **9**.

Thermal analyses.

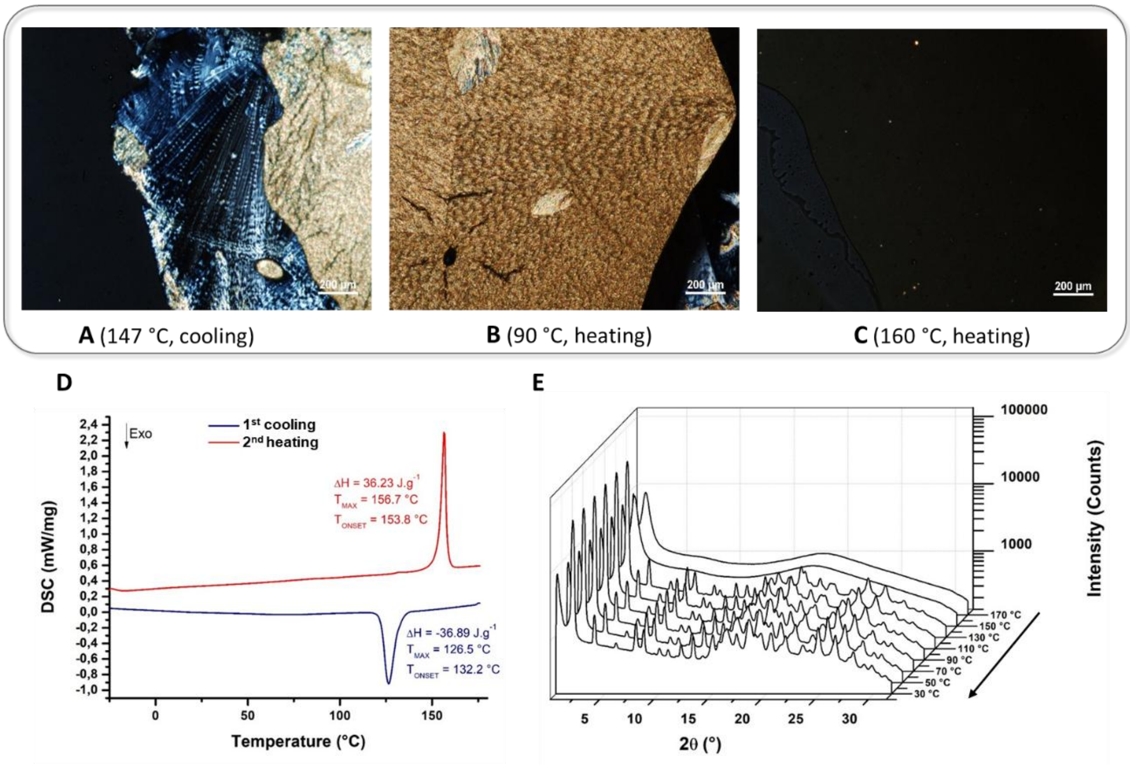

Figure S1. POM images of compound **3 ((S)-3,4,5-citro-C8(2))** (A) at 147 °C during the first cooling, (B) at 90 °C during the second heating, and (C) at 160 °C during the first heating. (D) DSC curve of compound **3** (scanning rate: 10 °C/min), (E) SAXS diffraction patterns recorded on compound **3** during cooling between 170 °C and 30 °C.

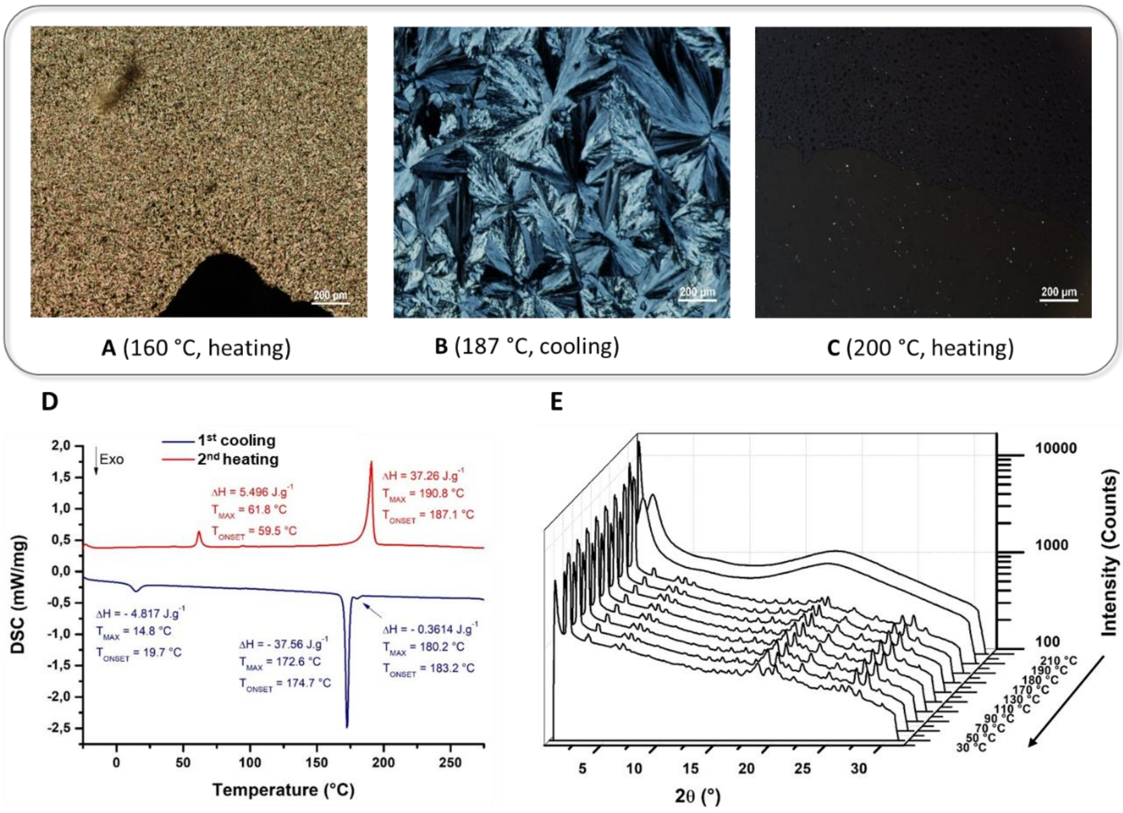

Figure S2. POM images of compound **4 ((S)-3,4-citro-C8(2))** (A) at 160 °C during the first cooling (B) at 187 °C during the first cooling and (C) at 200 °C during the first heating. (D) DSC curve of compound **4** compound (scanning rate: 10 °C/min), (E) SAXS diffraction diagram recorded on the compound **4** during cooling between 210 °C and 30 °C.

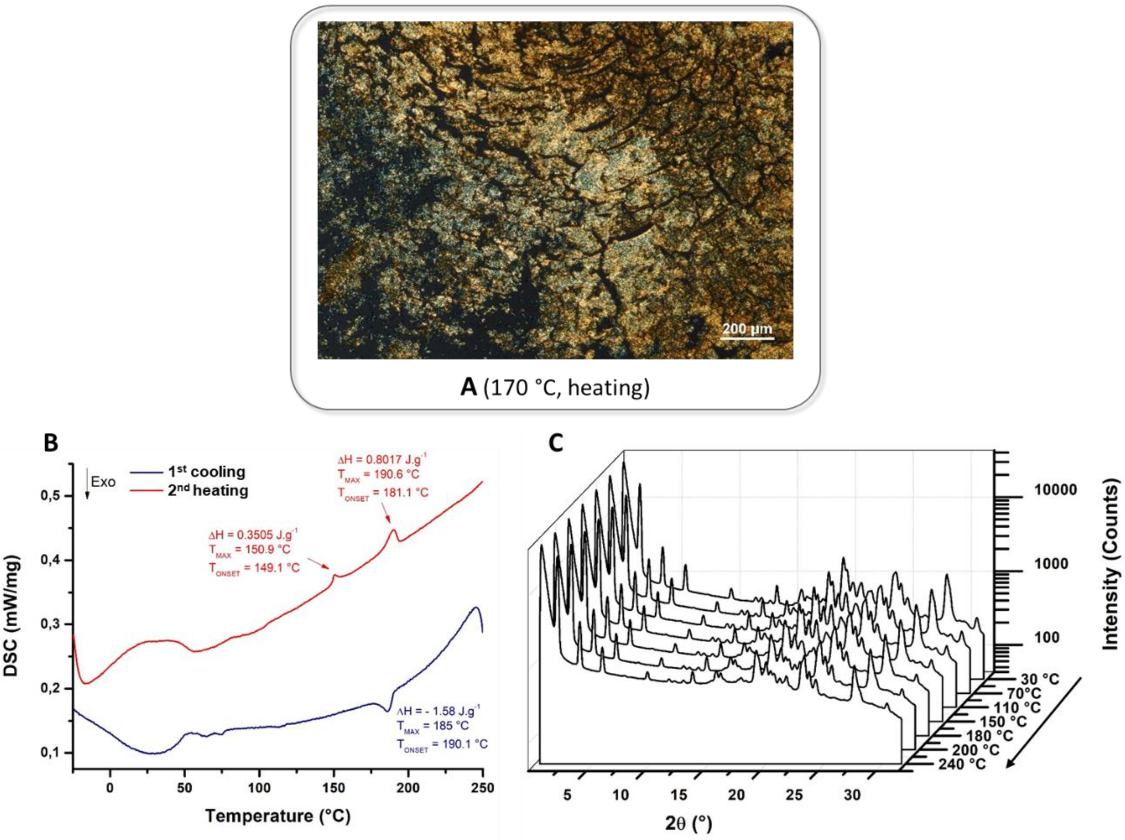

Figure S3. POM image of the compound **5 ((*S*)-4-citro-C8(2))** (A) at 170 °C during heating (B) DSC curve of the compound **5** (scanning rate: 10 °C/min), (C) SAXS diffraction diagrams recorded on the compound **5** during heating between 30 °C and 240 °C.

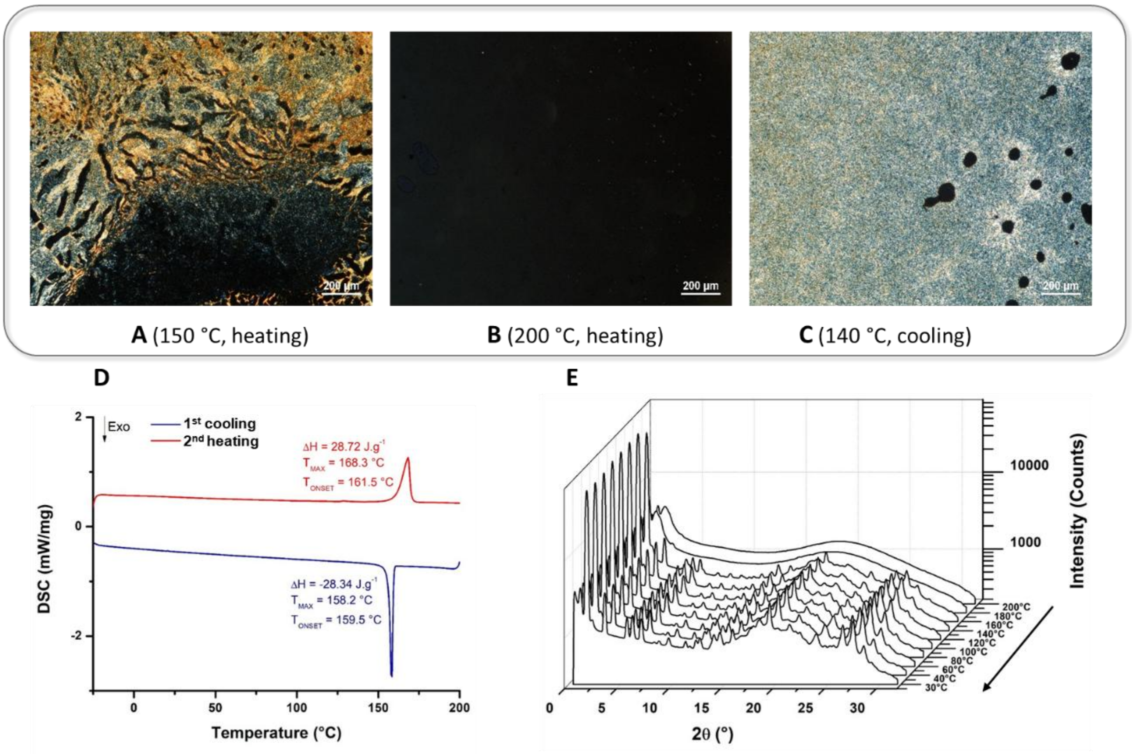

Figure S4. POM images of compound **6 ((R)-3,4-lact-C8**) (A) at 150 °C during the first heating (B) at 200 °C during the first heating and (C) at 140 °C during the first cooling. (D) DSC curve of compound **6** (scanning rate: 10 °C/min), (E) SAXS diffraction patterns recorded on the compound **6** during cooling between 200 °C and 30 °C.

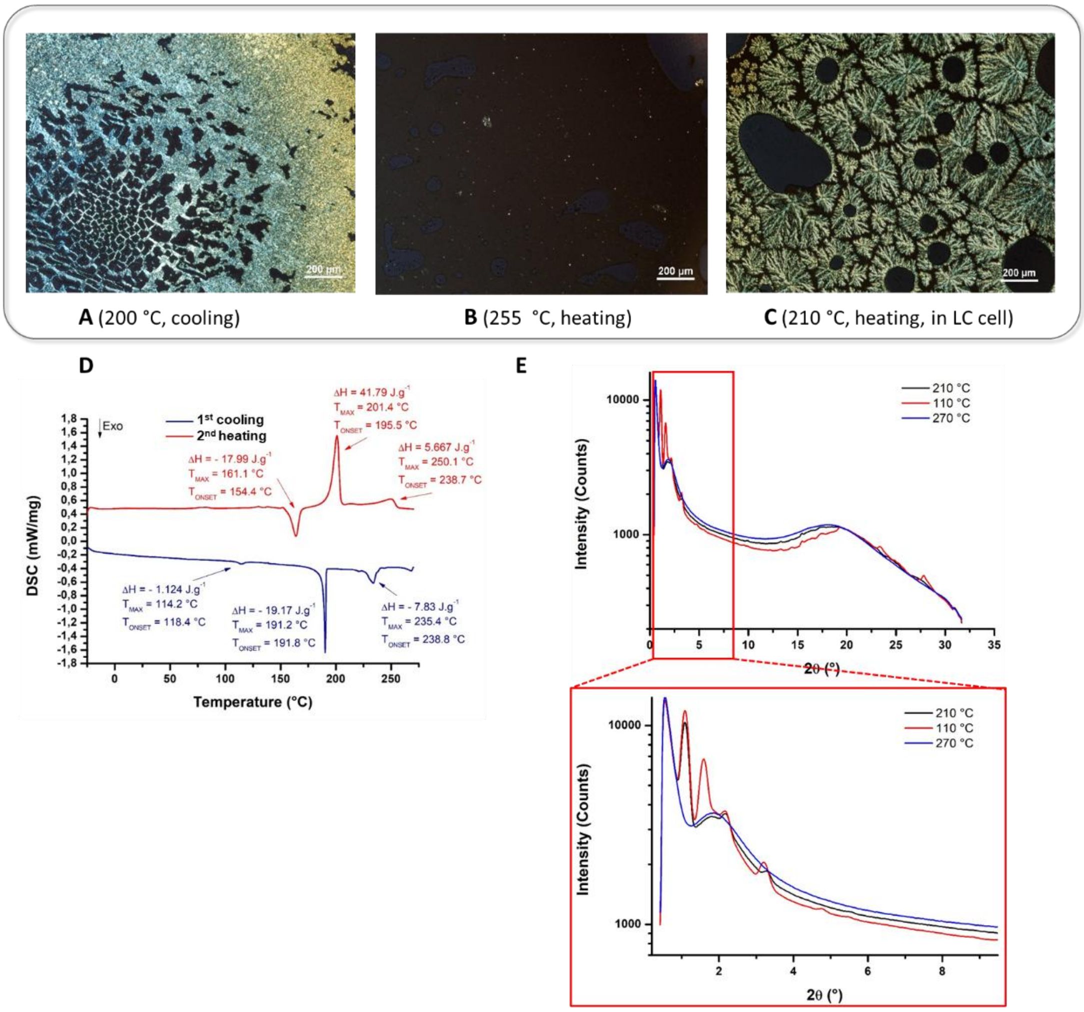

Figure S5. POM images of compound **8 ((S)-4-diol-C8)** (A) at 100 °C during the first cooling (B) at 255 °C during the first heating and (C) at 210 °C during the first heating in a 4 µm thick liquid crystal cell. (D) DSC curve of compound **8** (scanning rate: 10 °C/min), (E) SAXS diffraction diagrams recorded on compound **8** at 210 °C (black), 110 °C (red) and 270 °C (blue) during cooling.

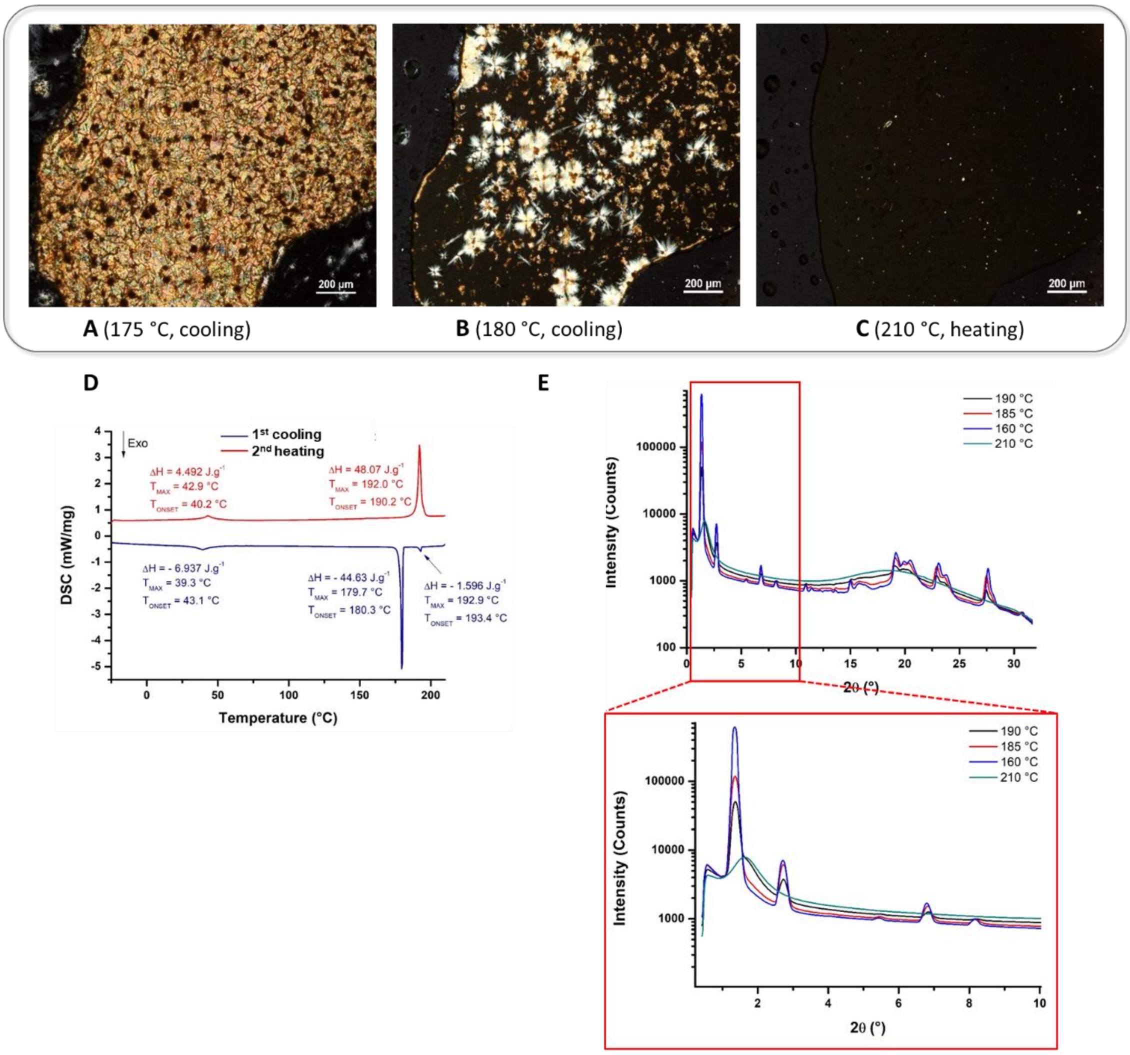

Figure S6. POM images of compound **9 ((S)-4-diol-C12)** (A) at 175 °C during the first cooling (B) at 180 °C during the first cooling and (C) at 210 °C during the first heating (D) DSC curve of compound **9** (scanning rate: 10 °C/min), (E) SAXS diffraction patterns recorded on compound **9** at 190 °C (in black), at 185 °C (in red), at 160 °C (in blue) and 210 °C (in green) during cooling.

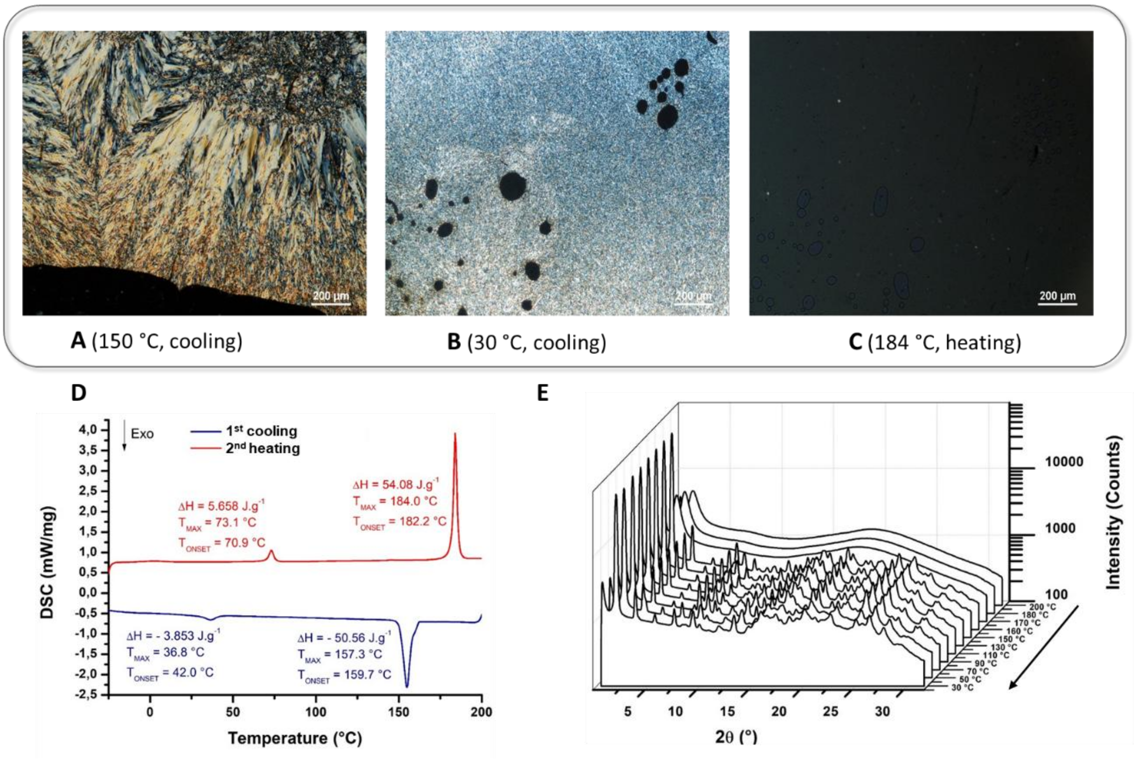

Figure S7. POM images of compound **10 (3,4,5-C8)** (A) at 150 °C, (B) at 30 °C during the first cooling and (C) at 184 °C during the first heating (D) DSC curve of compound **10** (scanning rate: 10 °C/min), (E) SAXS diffraction patterns recorded on compound **10** during cooling between 200 °C and 30 °C.

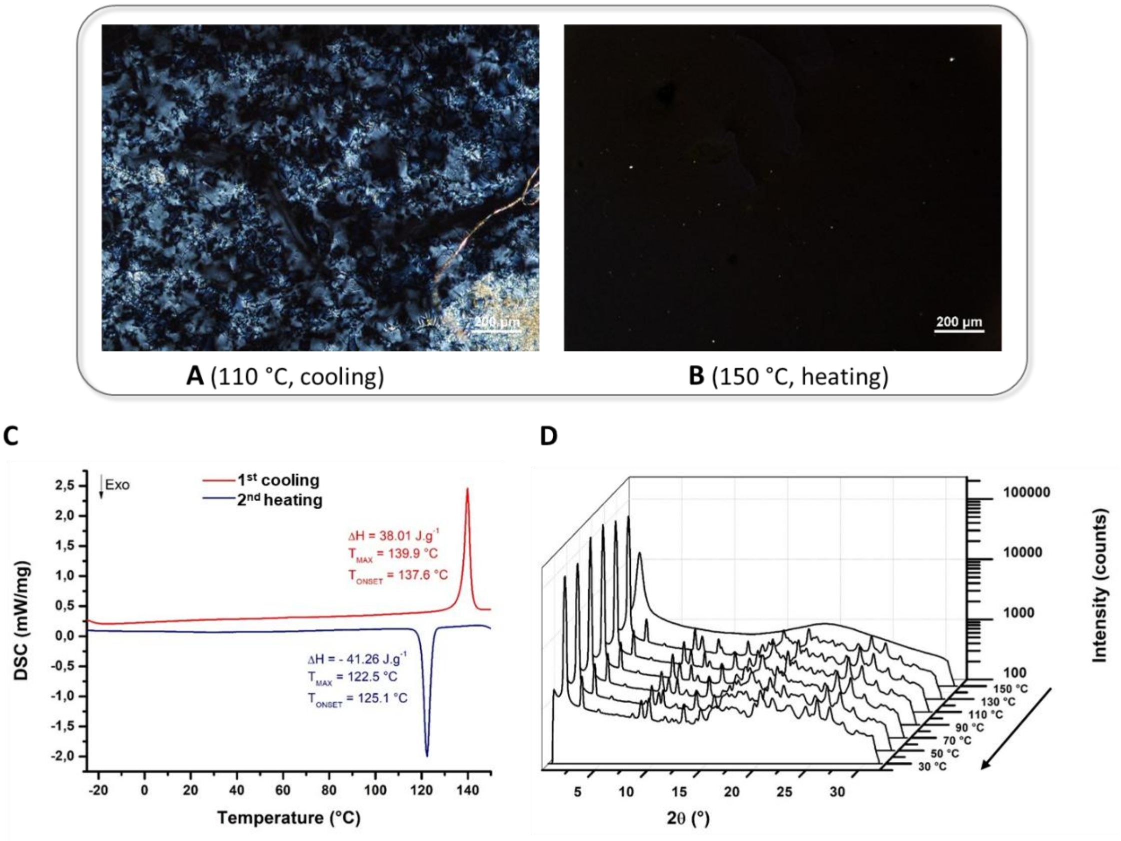

Figure S8. POM images of compound **11 (3,4,5-C12)** (A) at 110 °C during the first cooling and (B) at 150 °C during the first heating (a) DSC curve of compound **11** (scanning rate: 10 °C/min), (b) SAXS diffraction patterns recorded on compound **11** during cooling between 150 °C and 30 °C.

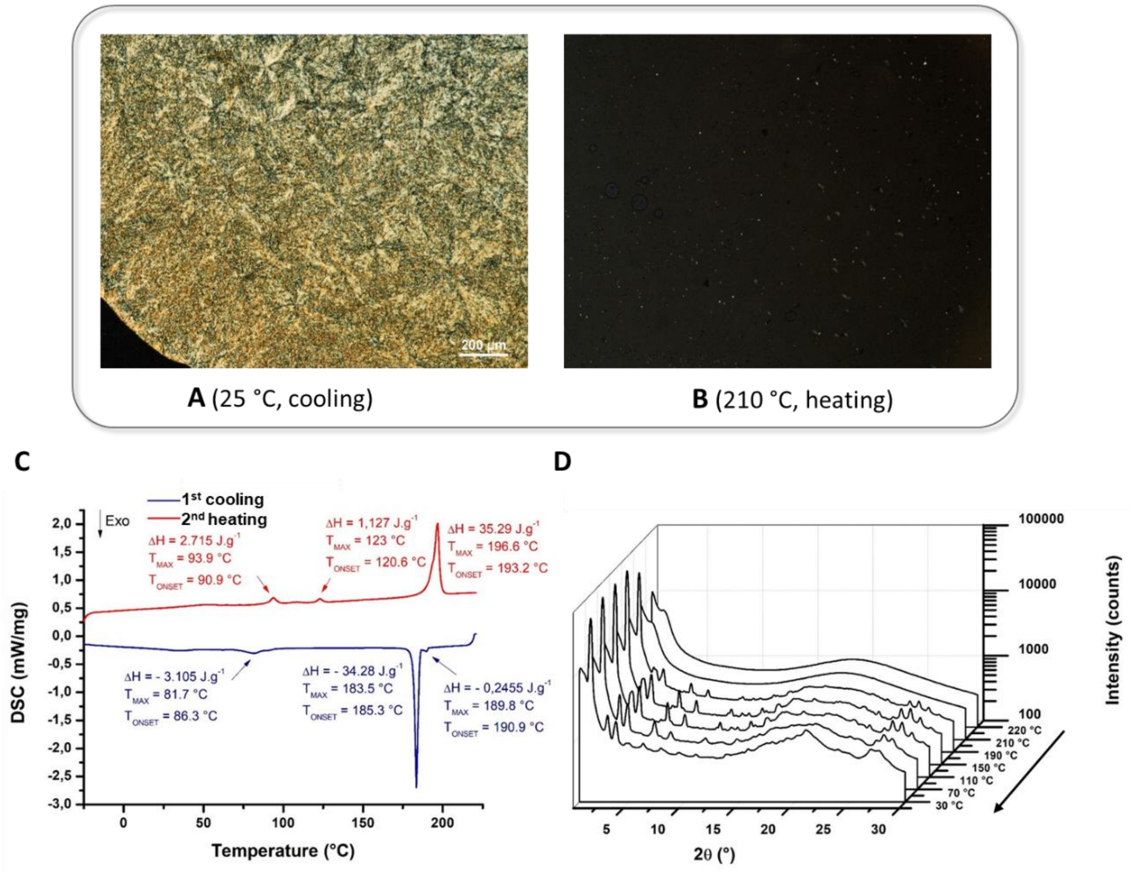

Figure S9. POM images of compound **12 (3,4-C12)** (A) at 25 °C during the first cooling and (B) at 210 °C during the first heating (C) DSC curve of compound **12** (scanning rate: 10 °C/min), (D) SAXS diffraction patterns recorded on compound **12** during cooling between 220 °C and 30 °C.

---

[1] T. Yasuda, T. Shimizu, F. Liu, G. Ungar, T. Kato, *J. Am. Chem. Soc.* **2011**, *133*, 13437–13444.

[2] Y. Chen, J. He, H. Lin, H. F. Wang, P. Hu, B. Q. Wang, K. Q. Zhao, B. Donnio, *Beilstein J. Org. Chem.* **2024**, *20*, 3263–3273.

[3] F. Camerel, G. Ulrich, P. Retailleau, R. Ziessel, *Angew. Chemie Int. Ed.* **2008**, *47*, 8876–8880.

[4] A. Jankowiak, D. Pociecha, J. Szczytko, P. Kaszyński, *Liq. Cryst.* **2014**, *41*, 1653–1660.

[5] S. Sankaranarayanan, A. Sharma, S. Chattopadhyay, *Tetrahedron: Asymmetry* **2002**, *13*, 1373–1378.

[6] A. Schreivogel, U. Dawin, A. Baro, F. Giesselmann, S. Laschat, J. Phys. Org. Chem. **2009**, 22, 484–494.

[7] R. R. Fayzullin, O. A. Antonovich, D. V. Zakharychev, Z. A. Bredikhina, A. V. Kurenkov, A. A. Bredikhin, *Russ. J. Org. Chem.* **2015**, *51*, 202–209.

[8] S. Glang, T. Rieth, D. Borchmann, I. Fortunati, R. Signorini, H. Detert, *European J. Org. Chem.* **2014**, 3116–3126.

[9] T. Cardolaccia, Y. Li, K. S. Schanze, *J. Am. Chem. Soc.* **2008**, *130*, 2535–2545.

[10] R. R. Parker, D. Liu, X. Yu, A. C. Whitwood, W. Zhu, J. A. G. Williams, Y. Wang, J. M. Lynam, D. W. Bruce, *J. Mater. Chem. C* **2021**, *9*, 1287–1302.
